# Evolution in metazoans of the TRPM channel family involves multiple gains and losses of genes and domains

**DOI:** 10.1093/molbev/msag098

**Published:** 2026-04-11

**Authors:** Marina Morini, Christina Bergqvist, Juan F Asturiano, Sylvie Dufour, Dan Larhammar

**Affiliations:** Laboratory Biology of Aquatic Organisms and Ecosystems (BOREA), National Museum of Natural History (MNHN), National Center for Scientific Research (CNRS), Institute of Research for Development (IRD), Sorbonne University, Paris, France; Grupo de Acuicultura y Biodiversidad, Instituto de Ciencia y Tecnologia Animal, Universitat Politècnica de València, Valencia, Spain; Department of Medical Cell Biology, Science for Life Laboratory, Uppsala University, Uppsala, Sweden; Grupo de Acuicultura y Biodiversidad, Instituto de Ciencia y Tecnologia Animal, Universitat Politècnica de València, Valencia, Spain; Laboratory Biology of Aquatic Organisms and Ecosystems (BOREA), National Museum of Natural History (MNHN), National Center for Scientific Research (CNRS), Institute of Research for Development (IRD), Sorbonne University, Paris, France; Department of Medical Cell Biology, Science for Life Laboratory, Uppsala University, Uppsala, Sweden; Guangdong Institute of Intelligence Science and Technology, Hengqin, Zhuhai, China

**Keywords:** transient receptor potential channel, TRPM, TRPS, melastatin, kinase domain, NUDIX domain, metazoans, vertebrates, evolution, phylogeny, synteny, tetraploidization

## Abstract

Transient receptor potential (TRP) ion channels of the melastatin family (TRPM) have eight members in mammals with a broad spectrum of functions. We investigated the evolution of this complex gene family across metazoans. The characteristic aminoterminal melastatin domain and the carboxyterminal NUDT9 homology domain with similarity to ADP-ribose pyrophosphatase were added to the common ancestor of TRPM and its sister channel TRPS. Gene duplications before the origin of bilaterians resulted in four TRPM genes: α, β, βlike, and γ. The two latter were discovered in this study. All four and TRPS are present in extant mollusks, while differential losses occurred in the other animal lineages. TRPS, TRPMβlike, and TRPMγ were lost in early chordates, meaning that the vertebrate ancestor started with TRPMα and β, both of which were duplicated before the first vertebrate tetraploidization 1R. The ancestor of the micro-RNA genes mir-211 and mir-204 was inserted in an intron of the ancestor of TRPM1/TRPM3. The TRPM6/TRPM7 ancestor acquired a kinase domain, probably a copy of the syntenic alpha protein kinase ALPK2/3 ancestor gene. Vertebrate 1R and gnathostome 2R together with local gene duplication and losses resulted in eight TRPM (TRPM1 to 8) in the gnathostome ancestor. In cyclostomes, extensive gene losses after the hexaploidization led to four TRPM. The teleost-specific tetraploidization 3R generated further TRPM ohnologs. The NUDT9 homology domain is retained in TRPM2 and TRPS but was lost repeatedly during TRPM evolution. Thus, the TRPM family displays considerable evolutionary variation with regard to gene and domain gains and losses.

## Introduction

**Figure 1:**
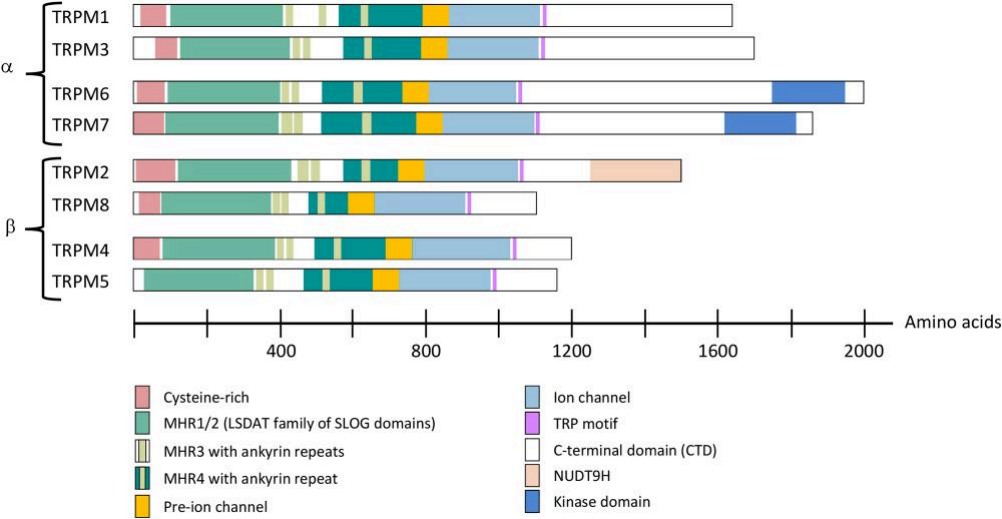
Schematic outline of the eight TRPM proteins in human. All members share the aminoterminal melastatin homology region (MHR). The MHR1-2 region is homologous to the SLOG (Smf-LOG) domain in bacteria and was named the LSDAT (LOG-Smf/DprA in TRPM) family of SLOG domains (Burroughs et al. 2015). LOG stands for the plant enzyme LONELY GUY (the mutant lacked a pistil and had a solitary stamen); the Smf protein in *Bacillus subtilis* is orthologous to DprA, DNA-processing protein A, in *E. coli*. All members also share the ion channel domain forming the ion pore, followed by a short motif of approximately 30 residues called the TRP domain. The extreme aminoterminus has a region with a number of cysteine residues, except that these have been lost in TRPM5 in human (but are present in TRPM5 in many other species). At the end of the carboxyterminal region TRPM2 has a NUDT9 homology domain and TRPM6 and 7 have a kinase domain.

The transient receptor potential (TRP) ion channels form a large superfamily with 28 members in eutherian mammals (Clapham 2003; Fleig and Penner 2004), except that TRPC2 has become a pseudogene in Old World monkeys including humans and other apes (Liman and Innan 2003). Eight of the members share a characteristic aminoterminal cytoplasmic region that was initially reported in 1998 for a protein found to be expressed in murine and human melanoma cells (Duncan et al. 1998). As this protein had a higher expression level in the less aggressive melanomas, it was hypothesized to limit melanoma growth and hence was named melastatin (Duncan et al. 1998). After observation of its ion channel similarity with previously described TRP proteins (Hunter et al. 1998) it was later named TRPM1. The same year another human TRP-related protein was reported (Nagamine et al. 1998) and as it was also found to possess the typical aminoterminal melastatin region it was subsequently named TRPM2. The other six mammalian TRP channels with the melastatin homology region were rapidly identified (Fleig and Penner 2004), see Fig. 1. It has been later suggested that melanoma suppression is not due to TRPM1 itself but to the microRNA gene miR-211 located in intron 6 of the human TRPM1 gene (Levy et al. 2010). However, miR-211 has been reported to have opposing effects on melanomas as well as several other tumors (for review see Ray et al. 2021). Furthermore, the TRPM1 protein has been found to promote, rather than inhibit, progression of acral melanoma, an aggressive form of melanoma (Hsieh et al. 2023). Thus, the name melastatin appears to be a misnomer.

The TRPM proteins have a broad range of functions that have been mostly studied in mammals. At least three TRPM members have roles as temperature sensors with TRPM8 reacting to cool temperatures which was highlighted by the Nobel Prize awarded to David Julius and Ardem Patapoutian in physiology or medicine in 2021. TRPM2 and TRPM3 are activated by warm temperatures, reviewed in Kashio and Tominaga (2022). Furthermore, TRPM4 and TRPM5 are sensitive to temperature (Talavera et al. 2005). As examples of the many functional roles of TRPM proteins (see Nilius and Owsianik 2011; Huang et al. 2020; Chubanov et al. 2024 for reviews) it can be mentioned that TRPM1 is involved in vision (Shen et al. 2009) and TRPM3 is also expressed in the retina (Behrendt 2022) as well as in many other parts of the nervous system (see Zhao and MacKinnon 2023). TRPM2 is highly expressed in the brain and may act as redox sensor (Sakaguchi and Mori 2020). Its closest relative TRPM8, in addition to its cold sensitivity, helps maintain core body temperature (Reimúndez et al. 2018) and is expressed in sensory neurons (Wang et al. 2023). TRPM8 is also expressed in human sperm where it may be involved in thermotaxis and chemotaxis (De Blas et al. 2009). TRPM4 is widely expressed too and participates for instance in heart functions, see (Winkler et al. 2017), while its closest relative TRPM5 has a more restricted distribution and has a key role in taste signaling (Aroke et al. 2020). TRPM6 contributes to Mg^2+^ homeostasis by reabsorption in the kidneys (Ehrenpreis et al. 2022) and the ubiquitously expressed TRPM7 is also involved in Mg^2+^ homeostasis (Schmitz et al. 2003) as well as embryonic development (Krapivinsky et al. 2014). An example of a relatively novel role in evolution is the implication of TRPM2 (Ahn et al. 2014) and TRPM4 (Fouchet et al. 2023) in uterus contractions in rodents.

Different TRPM members may be expressed in the same cell as shown by the observation of TRPM2, TRPM3, TRPM4, TRPM5, and TRPM7 in human and mouse pancreatic β cells (Benner et al. 2014). TRPM2 acts as a stimulator of insulin secretion by sensing nicotinic acid adenine dinucleotide phosphate in response to glucose (Yosida et al. 2014) and TRPM4 and TRPM5 are involved in the response to GLP-1, glucagon-like peptide 1 (Shigeto et al. 2015).

Mutations in TRPM genes cause a variety of diseases in humans (Jimenez et al. 2020; Chubanov et al. 2024) including night blindness for TRPM1 (van Genderen et al. 2009), epilepsy for TRPM3, heart disease for TRPM4, magnesium deficits for TRPM6 and TRPM7 and trigeminal neuralgia for TRPM7. Global knock-out of either TRPM6 (Woudenberg-Vrenken et al. 2011) or TRPM7 (Bates-Withers et al. 2011) in mouse is embryonically lethal, which is not the case for the other TRPM (TRPM1: Takeuchi et al. 2018; Gandini and Zamponi 2024; TRPM2: Gandini et al. 2024; TRPM3: Webster et al. 2020; TRPM4: Chen et al. 2020; TRPM5: Damak et al. 2006; TRPM8: Dhaka et al. 2007).

Beyond mammals, less is known about TRPM functions. The studies that have been done in the most common experimental non-mammalian vertebrates agree largely with TRPM roles in mammals. For instance, in chicken, TRPM5 is involved in taste detection (Yoshida et al. 2018) and TRPM8 mediates sensing of cold (Yamamoto et al. 2016), as it does in a poikilotherm species, the frog *Xenopus laevis* (Myers et al. 2009). In *Xenopus tropicalis* TRPM6 and TRPM7 are involved in neurogenesis during early development (Komiya et al. 2017). In zebrafish an extensive study of the tissue distribution of transcripts for its 11 TRPM genes reported expression during development, in tissues involved in ion homeostasis, and in sensory organs (Kastenhuber et al. 2013).

The mammalian TRPM members consist of up to ten protein domains or regions (Fig. 1). As we wished to analyze TRPM/TRPS evolution across metazoans, we describe here briefly these domains.

The aminoterminal melastatin homology region that distinguishes the TRPM family from other TRP families is approximately 700 amino acids. This region has been subdivided into four parts (Clapham 2003; Perraud et al. 2004) called melastatin homology region 1-4 (MHR1-4) (Fleig and Penner 2004) (Winkler et al. 2017). These four regions are homologous across the TRPM family members, but are not homologous to each other. MHR1 and MHR2 (Fig. 1) encompass approximately 250 amino acids and show similarity to many proteins including some that exist in bacteria (Burroughs et al. 2015) where the domain was named SLOG. One role of at least some SLOG domains seems to be to bind nucleotides. Indeed, the MHR1-MHR2 domains of TRPM proteins have been reported to form a binding site for ADP-ribose (ADPR) which leads to channel opening of TRPM2 in zebrafish (Huang et al. 2018). The MHR3 region consists of approximately 200 amino acid residues and has been reported to contain two ankyrin repeats, albeit divergent, as is the one in MHR4 (Burroughs et al. 2015). Ankyrin repeats are also present in TRPA, TRPC, TRPN, and TRPV. The MHR4 region is approximately 200 amino acids and is adjacent to the ion channel domain where it may interact with the channel's cytoplasmic loops and helices (Wang et al. 2018; Huang et al. 2020). The melastatin domain is preceded by a cysteine-rich domain (Burroughs et al. 2015) (Fig. 1).

The ion channel domain encompasses approximately 250 amino acids and is related to the large superfamily of potassium channels. It is preceded by a stretch of approximately 100 amino acids. TRPM1, TRPM3, TRPM6, and TRPM7 are permeable to the divalent cations Ca^2+^ and Mg^2+^ (Li et al. 2007). TRPM1 and TRPM3 have been reported to be constitutively active (open). TRPM6 and TRPM7 are important for uptake of Mg^2+^ as well as Zn^2+^ as demonstrated in mice (Chubanov et al. 2016; Mittermeier et al. 2019). Two members are permeable for both Ca^2+^ and Na^+^, namely TRPM2 and TRPM8 which are most closely related to each other. The remaining two, TRPM4 and TRPM5, are impermeable to Ca^2+^ but instead allow influx of Na^+^ or outflux of K^+^ depending on the voltage across the cell membrane (Launay et al. 2002; Ullrich et al. 2005). For these channels, opening is stimulated by Ca^2+^ binding on the cytoplasmic side.

The ion channel domain is followed by a motif of approximately 30 amino acid residues named the TRP domain (García-Sanz et al. 2004). It is followed by a rather unstructured stretch that varies considerably in size between the TRPM subtypes. Two alpha helices called C-terminal domain 1 and 2 have been observed. They interact with the aminoterminal melastatin domain, see for instance cryo-electron microscopy studies of TRPM4 (Winkler et al. 2017; Autzen et al. 2018) and TRPM8 (Palchevskyi et al. 2023). This interaction between N-terminal and C-terminal cytoplasmic domains of TRPM has been reported to be unique in the TRP superfamily. At the end, TRPM2 has a 300-amino-acid domain with homology to the mitochondrial protein NUDT9 (Perraud et al. 2001) while TRPM6 and TRPM7 have a serine/threonine kinase domain (Nadler et al. 2001; Runnels et al. 2001) (Fig. 1). The three TRPM members with catalytic domains, alpha kinase for TRPM6 and 7, or NUDT9H for TRPM2, have been named chanzymes for channel enzymes (Montell 2003), albeit TRPM2 was later found to have lost catalytic activity as mentioned above.

Previous evolutionary studies of the vertebrate TRPM family found that the eight mammalian members form two clades with four members in each: the α clade consists of the two pairs TRPM1-TRPM3 and TRPM6-TRPM7 and the β clade contains the two pairs TRPM2-TRPM8 and TRPM4-TRPM5 (Himmel et al. 2020). It has been suggested that a gene encoding the kinase domain was fused to the ancestor of TRPM6-TRPM7 and that the NUDT9H domain was ancestral in the β clade (Mederos and Schnitzler et al. 2008). It has also been reported that the TRPS gene, retrieved only in non-vertebrate metazoans such as the nematode *Caenorhabditis elegans* (where it is called ced-11), is the closest relative of the TRPM family (S stands for soromelastatin, ie a sister to the melastatin family) (Himmel et al. 2020) and interestingly also possesses not only a melastatin domain but also a NUDT9H domain. Thus, the NUDT9H domain is probably an ancient feature of the common ancestor of the TRPM-TRPS clade.

Phylogenetic studies in metazoans also revealed the ancient origin of the TRPMα and β clades as they both encompass sequences from cnidarian and various bilaterian species (Himmel et al. 2020). A few studies have addressed the functional roles of TRPM in non-vertebrates. In *Drosophila melanogaster* the single TRPM protein was reported to secrete Mg^2+^ and knockout of this gene is lethal (Hofmann et al. 2010), thereby the only TRP superfamily member out of 13 (Fowler and Montell 2013) to be essential for survival in drosophila. The single TRPM in drosophila is also involved in cold-sensing (Turner et al. 2016) and in chemosensation (terpene, like menthol sensitivity, Himmel et al. 2019). The nematode *Caenorhabditis elegans* has three TRPM genes (gtl1, gtl2, and gon2) which are involved in Mg^2+^ uptake and excretion (Kwan et al. 2008; Teramoto et al. 2010). In a cnidarian, *Hydra vulgaris*, a TRPM was found to mediate heat-nociceptive stimuli, like TRPM3 in mammals (Malafoglia et al. 2016).

We have previously investigated the evolution of the TRPV family and its gene duplications and losses with special focus on vertebrates (Morini et al. 2022). The unusual properties of the TRPM channels such as the melastatin homology region, the previously catalytic NUDT9H domain in TRPM2 and the active kinase domain in TRPM6-TRPM7, along with versatile functions and broad tissue distribution, make the TRPM family the most diverse within the TRP superfamily. We describe here a detailed analysis of the evolution of this family in metazoans, including a large set of representative species of various non-bilaterians, and bilaterian protostomes and deuterostomes. We had a special focus on the impact of whole genome doublings in vertebrates leading to ohnologs (ie paralogs resulting from genome duplications), namely the first and second round of tetraploidization (1R and 2R) in the early evolution of vertebrates and gnathostomes, respectively, as well as the third tetraploidization 3R at the origin of teleost fishes and the hexaploidization that followed the 1R in cyclostomes (Nakatani et al. 2021; Marlétaz et al. 2024; Yu et al. 2024). By analyzing adjacent genes on the chromosomes harboring the TRPM genes, we have been able to deduce with high precision how and when gene duplications took place in the vertebrate lineage. We also highlight the recruitment of various domains during TRPM gene evolution. Finally, we provide new insight on the evolution of mir211/204 linked to vertebrate TRPM1/TRPM3, a unique feature among the TRP family. We report early complexity of the TRPM-TRPS family in a metazoan ancestor and reveal more TRPM types than previously proposed, as well as a surprisingly high number of losses of genes and domains in various evolutionary lineages.

## Results and discussion

### Identification of new TRPM/TRPS sequences

The eight human TRPM proteins range in size from 1,100 amino acids for TRPM8 to over 2,000 amino acids for TRPM6 (Fig. 1). The human genes encompass 25 to 39 exons and the overall gene size from the first protein-coding exon to the last ranges from approximately 18 kb (TRPM5) to 585 kb (TRPM3). All of the TRPM members have been reported to display alternative splicing (Vázquez and Valverde 2006). The complexity and large sizes of the genes explain why database annotations are sometimes incomplete. To ensure as good coverage as possible of the major animal lineages, we have investigated a large number of genomes.

The TBLASTN results and phylogenetic analyses revealed the existence of many non-annotated TRPM sequences, including from several vertebrate species. We also identified and manually assembled several sequences. The global and detailed phylogenies also allowed us to rename some TRPS and some TRPM as described in the following sections. Species Latin names, gene accession numbers, current gene names in databases and our proposed phylogenetically based names are provided in Table S1.

Furthermore, our sequence alignment and phylogenetic analyses revealed that some sequences previously annotated as TRPM are in fact not related to the TRPM family. This is the case for some previously annotated cnidarian “TRPM6” or “TRPM6-like” sequences, which contain in fact only a kinase domain and none of the other TRPM domains. They had been wrongly annotated as TRPM, likely due to the homology with the kinase domain of vertebrate TRPM6 and 7 (for gene references see Table S1) (see also section “TRPM6,7 kinase domain”).

### Global phylogeny of TRPM/TRPS family in metazoans

**Figure 2:**
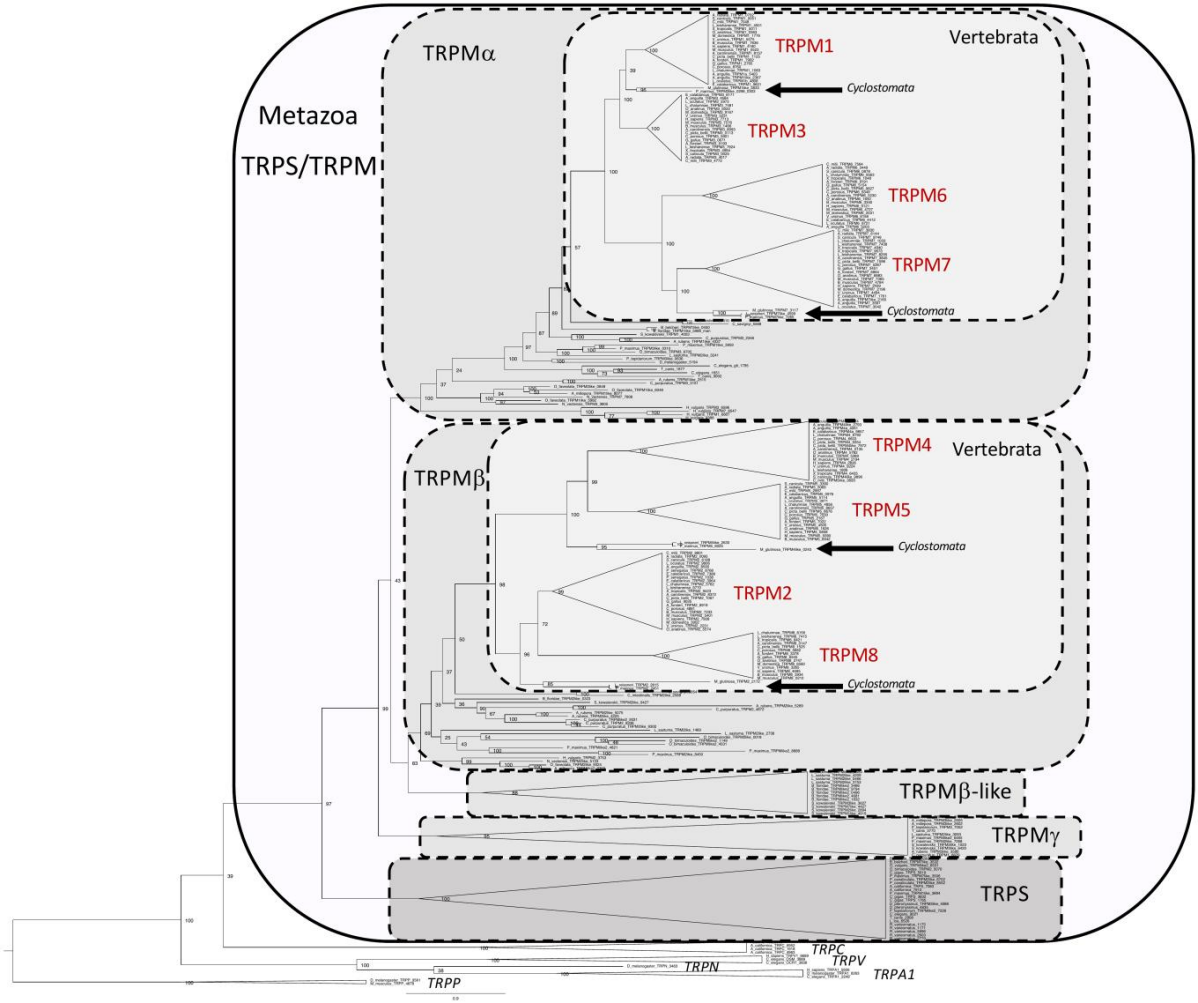
Global phylogenetic relationships of metazoan TRPM/TRPS sequences. Tree topology inferred with the phylogenetic maximum likelihood method from an alignment of 236 TRPM/TRPS amino acid sequences including 94 sequences from non-vertebrates and 142 sequences from vertebrates; a few sequences from more distantly related TRP families were included, namely TRPA, TRPM, TRPN, and TRPP from vertebrates and non-vertebrates. The tree was rooted with fruit fly and mouse TRPP sequences. Boostrap values over 1,000 replicates (%) are indicated. This global phylogenetic analysis clusters metazoan TRPM/TRPS sequences into two early clades, TRPM and its sister clade TRPS. TRPM is divided into four major clades, TRPMα, TRPMβ, and its sister clade TRPMβlike, and the clade TRPMγ. TRPMβlike and TRPMγ are novel TRPM types revealed by this study. Vertebrate TRPM sequences belong to the clades TRPMα and TRPMβ. Triangles highlight gnathostome TRPM1 to 8. See Figure S1a with all individual sequences represented. See Table S1 for sequence accession numbers.

To analyze the TRPM sequences in metazoans, we performed phylogenetic analyses using both maximum likelihood (ML) and Bayesian inference of 267 TRPM-TRPS amino acid sequences including 101 sequences from non-vertebrates and 166 sequences from vertebrates. We aimed at including species representative of major non bilaterian (cnidarian) and bilaterian taxa: protostome lophotrochozoa (mollusks and annelids); protostome ecdysozoa (arthropods and nematodes); deuterostome ambulacrarians (hemichordates and echinoderms); deuterostome chordates (cephalochordates, urochordates, vertebrates) focusing as much as possible on species with high quality genomes available (species are listed in the Table 1). The trees also included some (13) non-TRPM (TRPA, TRPC, TRPN, TRPP, and TRPV) sequences from vertebrates and non-vertebrates as outgroups and was rooted with fruit fly and mouse TRPP sequences. Figure 2 displays the tree resulting from ML analysis, with gnathostome sequences highlighted by triangles (gnathostome individual sequences are shown in the corresponding Figure S1a). The tree resulting from Bayesian inference is shown in Figure S1b. TRPA, TRPC, TRPN, and TRPV sequences were at the base of the phylogenetic trees. Both ML and Bayesian trees consistently display two main sister clades, one encompassing TRPS sequences and the other one the TRPM sequences.

**Table 1:** Taxonomic ranks of representative metazoan species investigated for TRPS/TRPM sequences.

**Cnidarians**	Scleractinians	*Orbicella faveolata*
		*Acropora millepora*
	Actiniarians	*Nematostella vectensis*
	Medusozoans	*Hydra vulgaris*
**Protostomes** **Lophotrochozoans**	Mollusks	*Pecten maximus*
		*Octopus bimaculoides*
	Annelids	*Lamellibranchia satsuma*
**Protostomes** **Ecdysozoans**	Nematodes	*Caenorhabditis elegans*
		*Toxocara canis*
	Arthropods	*Drosophila melanogaster*
		*Parasteatoda tepidariorum*
**Deuterostomes** **non-chordates** **Ambulacrarians**	Hemichordates	*Saccoglossus kowalevskii*
	Echinoderms	*Asterias rubens*
		*Strongylocentrotus purpurea*
**Deuterostomes chordates** **non-vertebrates**	Cephalochordates	*Branchiostoma floridae*
		*Branchiostoma belcheri*
	Urochordates	*Ciona intestinalis*
		*Ciona savignyi*
**Deuterostomes vertebrates**		
**Cyclostomes**	Lampreys	*Petromyzon marinus*
		*Lethenteron reissneri*
	Myxines	*Myxine glutinosa*
		*Eptatetrus bugeri*
**Chondrichthyans**	Holocephalans	*Callorhinchus milii*
	Elasmobranchs	*Scyliorhinus canicula*
		*Amblyraja radiata*
**Sarcopterygians**	Actinistians	*Latimeria chalumnae*
	Dipnoi	*Protopterus annectens*
		*Neoceratodus forsteri*
	Amphibians	*Microcaecilia unicolor*
		*Rhinatrema bivittatum*
		*Xenopus tropicalis*
		*Leptobrachium leishanense*
	Mammals	*Ornithorhynchus anatinus*
		*Vombatus ursinus*
		*Homo sapiens*
		*Mus musculus*
		*Balaenoptera musculus*
	Sauropsids	*Anolis carolinensis*
		*Chrysemys picta bellii*
		*Crocodylus porosus*
		*Gallus gallus*
		*Anas platyrhynchos*
		*Aptenodytes forsteri*
**Actinopterygian** **non-teleosts**	Polypterids	*Erpetoichthys calabaricus*
		*Polypterus senegalus*
	Holosteans	*Lepisosteus oculatus*
**Actinopterygian teleosts**	Elopomorphs	*Anguilla anguilla*
	Osteoglossomorphs	*Scleropages formosus*
	Clupeocephalans	Various species (see Table S1)

For details on teleost species, see Tables S1 and S2.

As previously reported by Himmel et al. (2020), all TRPS sequences are from non-vertebrate species. Our study supported the presence of TRPS sequences in protostome ecdysozoans (including nematodes such as *C. elegans* (ced-11) and arachnids among arthropods), in lophotrochozoans (mollusks: cephalopods, bivalves, gastropods), as well as in non-vertebrate deuterostome cephalochordates (amphioxus). Our study further supports that TRPS would have been lost repeatedly during metazoan evolution: in cnidarians, in some lophotrochozans (annelids), some ecdysozoans (in insects such as drosophila, among arthropods), in Ambulacraria and in Olfactores before the emergence of urochordates (see detailed evolutionary scenario in Figure S2a–c). Some of the sequences clustering in the TRPS clade in this tree had been previously annotated as TRPM in the NCBI database. This is the case for instance for sequences from mollusks (scallops TRPM1-like and TRPM-7 like, oyster TRPM2, octopus TRPM-like2), arthropod (mite TRPM3-like) as well as amphioxus TRPM7-like, that we now reassign as TRPS based on our phylogenetic analysis (see Table S1).

The TRPM sequences form two major clades, named TRPMα and TRPMβ according to Himmel et al. (2020), each of which includes non-vertebrate and vertebrate sequences (Fig. 2 and Figure S1a,b).

Gnathostome TRPM1, 3, 6, 7 sequences are closely related to each other in the metazoan TRPMα clade. Cyclostome TRPM sequences (from lampreys and hagfish) clustered basally to either gnathostome TRPM1 or TRPM7. In addition to vertebrate sequences, the α clade includes sequences from cnidaria (corals, sea anemone, and hydra), previously named “TRPM1 or TRPM1-like” in database wrongly referring to one of the vertebrate-specific TRPM subtypes. It also includes sequences from protostome ecdysozoans, such as the single drosophila TRPM, and three *C. elegans* TRPM (gtl-1, gtl-2, and gon-2). The TRPMα clade also contains sequences from protostome lophotrochozoans (annelids, mollusks) and non-vertebrate deuterostomes (echinoderms, hemichordates, cephalochordates, urochordates). All these sequences are referred to as “TRPM1, 3, 7 or TRPM1-like, 3-like, 7-like” in NCBI. In line with Himmel et al. (2020), we wish to point out that referring to one or the other of the vertebrate-specific TRPM subtypes is not relevant and is misleading, because the vertebrate subtypes arose in the vertebrate lineage, and therefore a phylogeny-based nomenclature “TRPMα” should be promoted in non-vertebrate metazoans.

Vertebrate TRPM2, 4, 5, 8 sequences are closely related to each other in the metazoan TRPMβ clade. Cyclostome sequences clustered at the base of the gnathostome TRPM2/8 and TRPM4/5 clades, respectively. The TRPMβ clade also includes sequences from cnidaria (corals, sea anemone and hydra), protostome lophotrochozoans (annelids, mollusks), and non-vertebrate deuterostomes (the same taxa as mentioned above), previously referred to as “TRPM2, TRPM2-like, or TRPM-like2” in NCBI. We noted that TRPMβ was missing in nematodes and arthropods (arachnid and insect) possibly due to its loss in their common ancestor (see Figure S2a,b). The broad species representation in α and β clades demonstrates the early origin of these two TRPM lineages in metazoan evolution. As for “TRPMα”, we support to name “TRPMβ” the sequences from non-vertebrate metazoans.

Our phylogenetic analysis performed on a large number of metazoan sequences revealed a new sister clade to the β clade, that we have named TRPMβlike. This clade, well supported by both ML and Bayesian analyses, includes sequences from protostome lophotrochozoans (annelid and mollusk) and non-vertebrate deuterostomes (multiple sequences from the hemichordate *Saccoglossus* and the cephalochordate *Amphioxus*). These sequences were previously referred to as “TRPM1-like, TRPM2-like, TRPM3-like, TRPM7-like, or TRPMlike-2” in NCBI highlighting the need for more accurate nomenclature as explained above. TRPMβlike may have been independently lost in ecdysozoans as well as twice in deuterostomes (in echinoderms and before the emergence of urochordates) (see Figure S2a–c).

Furthermore, the phylogenetic tree also showed an additional novel clade, sister to the TRPMα/β/βlike clades, that we have named TRPMγ. The TRPMγ clade, also well supported by both ML and Bayesian analyses, encompasses sequences from cnidaria (corals), protostome lophotrochozoans (annelid and mollusk), and ecdysozoans (arthropod arachnid and nematodes), and non-vertebrate deuterostomes (ambulacrarians: echinoderms and hemichordates), previously referred to as “TRPM, TRPM1, TRPM2-like, TRPM3, TRPM3-like, TRPM-like, TRPL-3” in database. This suggests that TRPMγ has an ancient origin in metazoans and that it would have been lost in insects (drosophila) as well as in the chordate ancestor (see Figure S2a–c).

The global phylogenies reveal that many gene duplications have occurred during the metazoan radiation, leading to the large diversity of the TRPM family in both non-vertebrates and vertebrates. The first gene duplication, which took place in a metazoan ancestor (or earlier), led to TRPS and TRPM. Further gene duplications of TRPM in the metazoan ancestor, before the emergence of cnidarians, resulted in the α, β, βlike, and γ TRPM genes (see Figure S2a). TRPMβlike and TRPMγ are not present in vertebrates, and were likely lost in the early evolution of the chordate lineage. Additional duplications of TRPMα and TRPMβ in vertebrates, led to four members in the α clade, TRPM1, 3, 6, 7, and four in the β clade, TRPM2, 4, 5, 8, in agreement with Himmel et al. (2020) (see Figure S2d).

### Investigation of vertebrate TRPM1, 3, 6, 7 subfamily (clade TRPMα)

#### Phylogeny of vertebrate TRPM1, 3, 6, 7 subfamily

In order to further investigate vertebrate TRPM, we analyzed the TRPM1, 3, 6, 7 subfamily (belonging to clade TRPMα) by complementary phylogenetic and syntenic approaches. The phylogenetic analysis focused on vertebrate TRPM sequences (Figure S3a,b) and included a larger number of vertebrate sequences than in the global phylogeny (Fig. 2), with particular emphasis on teleost sequences to assess the impact of the teleost-specific whole-genome duplication (3R). The phylogeny analysis thus included a total of 181 vertebrate TRPM1, 3, 6, 7 amino acid sequences, including 5 sequences from cyclostomes, 12 sequences from chondrichthyans, 98 from actinopterygians, and 66 sequences from sarcopterygians. The phylogenetic tree in Figure S3a was rooted with reedfish TRPM2 sequence (for sequence references see Table S1). In agreement with Fig. 2, vertebrate TRPM1, 3, 6, 7 sequences form two main clades, one containing the two sister clades TRPM1 and TRPM3 while the other consists of the two sister clades TRPM6 and TRPM7.

##### Phylogeny of TRPM1

TRPM1 is present in all gnathostomes investigated, with chondrichthyan TRPM1 sequences branching at the base of osteichthyan (actinopterygian and sarcopterygian) sequences, in agreement with the phylogeny of vertebrates. Among actinopterygians, a single TRPM1 sequence was present in representatives of cladistian (reedfish) and holostean (spotted gar) lineages, while two TRPM1 (named TRPM1a and TRPM1b) were retrieved in most teleosts investigated. The presence of teleost TRPM1a and b paralogs has been previously reported in some teleosts, such as zebrafish (NCBI) and notothenioids (York and Zakon 2022). The two TRPM1 paralogs in teleosts likely resulted from the teleost WGD (3R) event, as supported by the synteny analyses (see below). A species-specific loss of TRPM1a would have occurred in a silurid, catfish, *Ictalurus punctatus* (see Figure S2f). Concerning salmonids, our study revealed the presence of three TRPM1 in *Salmo* (Atlantic salmon, *Salmo salar*) and *Oncorhynchus* (rainbow trout, *O. mykiss*). Two full sequences, that had been previously reported, clustered with the northern pike (*Esox lucius*) TRPM1a and TRPM1b, respectively; a third sequence that we manually reconstructed from several partial sequences (see Table S1) clustered with the pike TRPM1a.The tree suggests that the salmonid TRPM1a additional paralog resulted from the salmonid-specific WGD (4R) which was supported by the synteny analysis; in contrast, one of the 4R-duplicated TRPM1b paralog would have been lost. Among sarcopterygians, the TRPM1 sequence of representatives of basal groups (actinistian coelacanth, dipnoi African, and Australian lungfish) clustered at the base of the tetrapod clade, in agreement with the species phylogeny. We manually reconstructed a TRPM sequence in the sea lamprey, that was previously annotated TRPM3-like in NCBI, but that branched, together with the myxine TRPM1-like sequence, at the base of the gnathostome TRPM1 sequences. We propose to rename these cyclostome sequences TRPM1, in agreement with Himmel et al. (2020).

##### Phylogeny of TRPM3

TRPM3 sequences were retrieved in all gnathostomes investigated, again with the chondrichthyan sequences as a sister clade to the osteichthyan sequences. Different from TRPM1, a single sequence of TRPM3 was retrieved in all teleosts investigated including representatives of basal groups, elopomorphs (eel) and osteoglossomorphs (bonytongue). This suggests an early loss after 3R of one of the duplicated paralogs (see Figure S2f,g). This hypothesis was supported by the synteny analysis. Two TRPM3 paralogs were found in salmonids (Atlantic salmon and rainbow trout) clustering with the single pike TRPM3, suggesting that they arose from salmonid 4R as also supported by the synteny analysis. It is noteworthy that all TRPM3 sequences showed short branches in the phylogeny tree, reflecting a strong conservation of TRPM3 sequences across gnathostome radiation. We did not find any cyclostome sequence clustering with the TRPM3 clade, suggesting the loss of TRPM3 in this lineage, in agreement with Himmel et al. (2020).

##### Phylogeny of TRPM6

TRPM6 is present in all gnathostomes investigated, with chondrichthyan sequences basal to the osteichthyan sequences. In sarcopterygians, TRPM6 was found in most species investigated, with the exception of a protopterid (the West African lungfish), and two amphibians (a gymnophiona, the two-lined cecilian, and an anuran, the Leishan spiny toad), while it was present in *Xenopus tropicalis* (see Figure S2e). As for TRPM3, a single TRPM6 gene was retrieved in all teleost species investigated, suggesting an early loss of one 3R-paralog. This was supported by the synteny analysis. The phylogeny analysis allowed us to rename some previously annotated sequences, such as one of the cod “TRPM7” which is in fact a TRPM6 (see Table S1). In salmonids (Atlantic salmon and rainbow trout) two TRPM6 are present, and clustered with the single pike TRPM6, in agreement with their likely origin from the salmonid 4R (see synteny analysis). This allowed us to rename one of these two salmonid TRPM6 paralogs, which had been previously annotated as “TRPM7” (see Table S1). Notably, the phylogenic tree revealed longer branches for TRPM6 sequences in teleosts as compared to other actinopterygians and to chondrichthyans and sarcopterygians. This may suggest some functional divergence of TRPM6 during the teleost radiation. As for TRPM3, we did not find any cyclostome sequence clustering with the TRPM6 clade, suggesting the loss of TRPM6 in this lineage, also in agreement with Himmel et al. (2020) (see Figure S2d).

##### Phylogeny of TRPM7

BLAST searches revealed that TRPM7 was present in all gnathostomes investigated, with chondrichthyan sequences basal to osteichthyan sequences. Two TRPM7 paralogs are present in representatives of different groups of amphibians (in a gymnophiona, *Microcaecilia unicolor*, and in anurans *Xenopus tropicalis and Leishan spiny toad*), indicating an amphibian-specific local gene duplication of TRPM7 (see Figure S2e), which was further investigated by synteny analysis. One of the duplicated amphibian TRPM7 paralogs showed remarkably long branches in anurans, indicating greater evolutionary divergence as compared to the “classical” TRPM7. A third TRPM7 paralog was retrieved in another gymnophiona, *Rhinatrema bivittatum,* reflecting an additional species-specific duplication of TRPM7. Among teleosts, two TRPM7 paralogs were retrieved in representatives of basal groups, elopomorphs (eel) and osteoglossomorphs (bonytongue) that may result from teleost 3R. In contrast, only a single TRPM7 gene could be found in most other teleosts studied, suggesting that one of the 3R-paralog would have been lost in the clupeocephalan lineage (see Figure S2f,g). This was supported by the synteny analysis. However, two TRPM7 paralogs were retrieved in some clupeocephalan species such as in the pike and in the seabream. These paralogs clustered together in the phylogeny tree for each species, respectively, suggesting species-specific duplications. Among salmonids, two TRPM7 paralogs are present in Atlantic salmon, while only one was retrieved in rainbow trout. The two Atlantic salmon paralogs may result from salmonid 4R. These hypotheses were tested by the synteny analyses. Cyclostome (lamprey and myxine) sequences, non-annotated or previously annotated TRPM7 or TRPM7-like, clustered at the base of the gnathostome TRPM7 clade.

#### Conserved synteny of the TRPM1, 7 genomic region

The chromosomal locations of the TRPM1 and TRPM7 genes are shown in Figure S4a for a broad range of vertebrates: three sarcopterygians (human, duck, and western clawed frog), one chondrichthyan (spotted catshark), two non-teleost actinopterygians (cladistian reedfish and holostean spotted gar), ten teleosts (European eel, Asian bonytongue, Atlantic herring, zebrafish, Atlantic cod, fugu, medaka, turbot, Northern pike, and Atlantic salmon), and a cyclostome, the sea lamprey (see Table S2 for references and location of TRPM and neighboring genes). The genomic region of the reedfish was used as a template, and eight neighboring genes were investigated. Previously unidentified TRPM1 and TRPM7 neighboring genes in some vertebrate genome databases were retrieved using the TBLASTN algorithm of the Ensembl Genome Browser website (see Table S2). For teleost synteny an additional neighboring gene was investigated. A strong conservation of this syntenic genomic region was observed throughout all gnathostomes, supporting the orthology of TRPM1 and TRPM7, respectively, across chondrichthyans, sarcopterygians, and actinopterygians, as well as cyclostomes for TRPM7 (the location of lamprey TRPM1 on an isolated short scaffold does not provide any synteny information). TRPM1 and TRPM7 are located in the same genomic region in all gnathostomes investigated, in line with the hypothesis that they originated from a local duplication of an ancestral pre-vertebrate TRPMα gene (see Figure S2d). This duplication would have given the ancestor of TRPM1/3 on the one hand and on the other hand the ancestor of TRPM6/7. The subsequent duplication of TRPM1/3 into TRPM1 and TRPM3, as well as of TRPM6/7 into TRPM6 and TRPM7 coincides with whole genome duplication (see Figure S2d) as deduced from the paralogon analyses (see below). Duplicated TRPM7 paralogs were present in amphibians and located in tandem as shown for *Xenopus tropicalis* (Figure S4a), supporting a local duplication of TRPM7 in the amphibian lineage, in agreement with the phylogenetic analysis.

Some of the neighboring genes are duplicated in teleosts. In contrast, the non-teleost actinopterygians, reedfish, and spotted gar, possess only a single copy of these genes, supporting the hypothesis that the TRPM1 and TRPM7 genomic region has been duplicated in the teleost ancestor as a result of 3R. In the Atlantic salmon, four copies of some neighboring genes have been identified, reflecting the additional duplication of the genomic region, as a result of the salmonid-specific 4R. In teleosts, the duplicated TRPM1 genes, named “a and b” according to Zfin nomenclature for teleost 3R-ohnologs, have been retained in most species. The two Asian bonytongue TRPM1a and b paralogs are located in tandem on the same chromosome (chromosome 11) (Figure S4a). Further studies could assess whether this tandem location results from a recombination between bonytongue chromosomes 11 and 7. The salmonid lineage has inherited the two 3R-TRPM1a and b ohnologs, which were further duplicated in 4R. The Atlantic salmon retained three of the four ohnologs, which we named TRPM1aα, TRPM1aβ, and TRPM1bα, according to the “α/β” nomenclature for salmonid 4R-ohnologs (Mungpakdee et al. 2008; Robertson et al. 2017). Differently from 3R ohnologs TRPM1a and b which have been conserved in most teleosts, duplicated 3R ohnologs for TRPM7a and b have been retained only in basal teleosts, elopomorphs (eel), and osteoglossomorphs (bonytongue). The single TRPM7 ohnolog conserved in other teleosts, corresponding to TRPM7a, was positioned in each species on the same paralogon (Figure S4a). This suggests that the loss of TRPM7b may have occurred once, in a clupeocephalan ancestor, after the emergence of elopomorphs and osteoglossomorphs (Figure S2f,g). Two TRPM7a genes are present in tandem in the genome of the pike supporting their origin by a species-specific local gene duplication, as also suggested by the phylogeny analysis. Two TRPM7a genes were retrieved in the seabream, also clustering together, in agreement with another species-specific duplication, but in that case only one of the paralogs was retained in the “classical” paralogon (chromosome 6), while the other one was translocated to another chromosome (chromosome 4) (data not shown). In the Atlantic salmon, synteny analysis supports that the two TRPM7a ohnologs resulted from the salmonid 4R, so that they were named TRPM7aα and TRPM7aβ.The presence of TRPM1 and TRPM7 in the lamprey has been reported by Himmel et al. (2020). The current status of the lamprey genome locates TRPM7 in this syntenic region, but TRPM1 is on an isolated scaffold.

#### Conserved synteny of the TRPM3, 6 genomic region

The genomic region of the reedfish was used as a template. TRPM3 and TRPM6 are located in the same genomic region in all gnathostomes (see Figure S4b), in agreement with the hypothesis of the ancient local duplication of their common ancestral pre-vertebrate TRPMα gene (see Figure S2b). Seven neighboring genes were investigated (see Table S2 for references and location of TRPM and neighboring genes). A strong conservation of this syntenic region was observed throughout all gnathostomes investigated (see Figure S4b), supporting the orthology between TRPM3 and TRPM6, respectively, across gnathostomes. In the lamprey, no TRPM was retrieved in this genomic region. Some of these neighboring genes have duplicates on a separate chromosome in teleosts, in agreement with the duplication of this genomic region via the teleost 3R. However, TRPM3 and TRPM6, as well as some neighboring genes have lost their respective 3R duplicate in all teleosts studied, including basal representative such as the eel, suggesting that loss of the duplicate happened before the teleost radiation. In Atlantic salmon some neighboring genes have three ohnologs, in agreement with the 4R genome doubling in salmonids. The single TRPM3 and TRPM6 3R-ohnologs were duplicated in salmonids via the 4R and we named the conserved 4R-ohnologs TRPM3α, TRPM3β, TRPM6α, and TRPM6β.

#### Paralogon for vertebrate TRPM1, 3, 6, 7 genes

**Figure 3:**
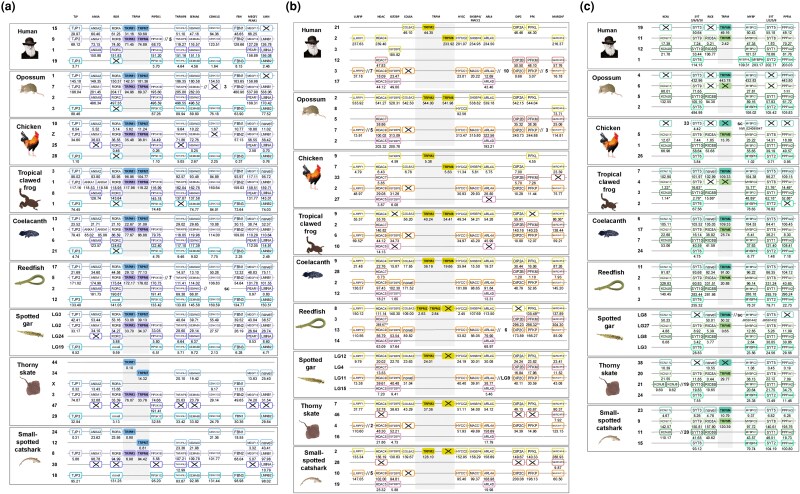
Paralogons for vertebrate TRPM genes with neighbors. a) Paralogon for vertebrate TRPM1, 3, 6, 7 genes with neighbors. Gnathostome chromosomal regions harboring the genes of the TRPMα subfamily: the TRPM genes 1 and 7 are in a chromosomal region with several neighboring genes that have related genes also on the chromosome where TRPM genes 3 and 6 are located, as well as two related chromosomal regions that lack TRPM genes. Sequence-based phylogenetic analyses (Figure S5a) show that all of these gene families underwent duplications in time period of the tetraploidization events 1R and 2R. Thus, these chromosomal regions are in agreement with quadruplication of an ancestral vertebrate chromosome. The species shown are human, opossum, chicken, tropical clawed frog, reedfish, spotted gar, thorny skate, and small-spotted catshark. The number below each gene shows the position on the chromosome. The order of the genes along the chromosomes has been re-shuffled to highlight the similarities. See Table S3 for TRPM and neighboring gene sequence accession numbers. b) Paralogon for vertebrate TRPM2 and TRPM8 genes with neighbors. Gnathostome chromosomal regions harboring the TRPMβ subfamily genes TRPM2 and TRPM8. These TRPM genes are in chromosomal regions with several neighboring genes that have related genes also in other chromosomal regions. Sequence-based phylogenetic analyses (Figure S5b) show that all of these gene families underwent duplications in time period of the tetraploidization events 1R and 2R. Thus, these chromosomal regions are in agreement with quadruplication of an ancestral vertebrate chromosome. However, chromosomal rearrangements have disrupted some of the chromosomes and translocated blocks of genes. Several of the gene duplicates have been lost. Nevertheless, the ancestral constellations of genes deduced from these species reveals a chromosomal quartet that arose in the early gnathostome tetraploidizations. The species shown are as in Fig. 3a. The number below each gene shows the position on the chromosome. The order of the genes along the chromosomes has been re-shuffled to highlight the similarities. See Table S3 for TRPM and neighboring gene sequence accession numbers. c). Paralogon for vertebrate TRPM4 and TRPM5 genes with neighbors. Gnathostome chromosomal regions harboring the TRPMβ subfamily genes, TRPM4 and TRPM5. These TRPM genes are in chromosomal regions with several neighboring genes that have related genes also in other chromosomal regions. Sequence-based phylogenetic analyses (Figure S5c) show that all of these gene families underwent duplications in time period of the tetraploidization events 1R and 2R. Thus, these chromosomal regions are in agreement with quadruplication of an ancestral vertebrate chromosome. The species shown are as in Fig. 3a. The number below each gene shows the position on the chromosome. The order of the genes along the chromosomes has been re-shuffled to highlight the similarities. See Table S3 for TRPM and neighboring gene sequence accession numbers.

The chromosomal region with the TRM1-TRPM7 gene pair and the region with TRPM3-TRPM6 gene pair share representatives from several syntenic gene families. All of these neighboring gene families also have members on either or both of two additional chromosomes, see Fig. 3a (see Table S3 for references and location of TRPM and neighboring genes). The syntenic gene families display sequence-based phylogenies (Figure S5a) and have species distribution (Fig. 3a) that are consistent with duplications in the same time period as the genome doublings (1R and 2R) in the early evolution of vertebrates and gnathostomes. Likewise, TRPM1 and TRPM3 arose from their common ancestor around this time, as did TRPM6 and TRPM7. Four of the neighboring gene families are full quartets in several of the species investigated, albeit each one is missing one member in some species, namely ROR, TNFAIP, SEMA6, and LMN (Fig. 3a). Six syntenic gene families have three members, together comprising a quartet of related chromosomal regions, namely TJP, ANXA, PIP5K1, CSNK1G, FBN, and MEGF/PEAR1. In some species, blocks of genes have been translocated to other chromosomes. Four species display chromosome quartets with no translocations at all, namely chicken, *Xenopus tropicalis*, *Latimeria chalumnae*, and spotted gar. Thus, the TRPM1, 3, 6, 7 genes are located in the same paralogon. Additional TRPM1, 3, 6, 7 ohnologs would have been expected from the quartet of chromosomes as the TRPM1/7 and TRPM3/6 chromosomes appear to have arisen in 1R based upon the chromosome phylogeny reported in (Marlétaz et al. 2024). This implies four gene losses after 2R, namely the 2R duplicates for each of the four TRPM genes.

### Investigation of vertebrate TRPM2, 4, 5, 8 subfamily (clade TRPMβ)

#### Phylogeny of vertebrate TRPM2, 4, 5, 8 subfamily

We analyzed the TRPM2, 4, 5, 8 subfamily (belonging to clade TRPMβ) by complementary phylogenetical and syntenic approaches, in order to focus on vertebrate TRPM sequences and to assess the impact of the teleost-specific whole-genome duplication (3R). The phylogeny analyses included a total of 174 vertebrate TRPM2, 4, 5, 8 amino acid sequences, including 6 sequences from cyclostomes, 8 sequences from chondrichthyans, 92 from actinopterygians, and 68 sequences from sarcopterygians. The phylogenetic tree in Figure S3b was rooted with the reedfish TRPM1 sequence (for sequence references see Table S1). In agreement with Fig. 2, vertebrate TRPM2, 4, 5, 8 sequences clustered in two main clades, with a sequence from lamprey at the base of each clade. One of the clades included TRPM2 and TRPM8 sequences, and the other one TRPM4 and TRPM5 sequences.

##### Phylogeny of TRPM2 and TRPM8

TRPM2 is present in all groups of gnathostomes investigated (Figure S3b). TRPM8 is found only in sarcopterygians, including basal representatives, the actinistian coelacanth and the dipnoi Australian and African lungfish. TRPM8 is duplicated in *Xenopus tropicalis*. We note that one of the copies has a higher evolutionary rate. The sarcopterygian TRPM8 sequences clustered as a sister clade to the gnathostome TRPM2 sequences. This suggests that the duplication that led to TRPM2 and TRPM8 may have occurred early in the vertebrate lineage and that TRPM8 would have been retained only in sarcopterygians, but was lost in chondrichthyans and actinopterygians, as proposed by Himmel et al. (2020). Two TRPM2 sequences were found in a sarcopterygian, a gymnophiona, *Rhinatrema bivittatum* (while no TRPM2 was found in another gymnophiona, *Microcaecilia unicolor*) (see Figure S2e). Some non-teleost actinopterygians also possess two TRPM2 sequences, such as cladistian reedfish *Erpetoichthys calabaricus* and *Polypterus senegalus*, with the phylogeny indicating a cladistian lineage specific duplication. Two TRPM2 were also found in some teleosts, Astyanax, stickleback, and pike, clustering together in the phylogenetic tree for each species; this suggests that they arose from independent gene duplications and not from the teleost-specific 3R. One of the teleost 3R-ohnologs would have been lost shortly after 3R. Altogether, this suggests that TRPM2 species-specific gene duplication occurred repeatedly during vertebrate radiation, in some amphibians, in cladistians and some teleosts (see Figure S2e–g). A single TRPM2 sequence was retrieved in salmonids suggesting the loss of 4R-duplicated ohnologs. The origin of duplicated TRPM2 paralogs in teleosts was further analyzed by synteny (see below). Sequences from cyclostomes, lampreys (*Petromyzon marinus*, *Lethenteron reissneri)*, and myxine (*Myxine glutinosa*) previously annotated as TRPM2 or not annotated in NCBI, cluster at the base of the gnathostome TRPM2 and TRPM8 clades, as also observed in the global phylogeny (Fig. 2). This supports the presence of TRPM from the β clade in cyclostomes, differently from previous report by Himmel et al. (2020). We propose to name these sequences TRPM2/8. This suggests that the local duplication that led to TRPM2 and TRPM8 may have arisen in the gnathostome ancestor after it had diverged from the cyclostome lineage.

##### Phylogeny of TRPM4

TRPM4 sequences are present in chondrichthyan holocephalan (elephant shark) and elasmobranch (spotted catshark) but missing in another elasmobranch (thorny skate) (Figure S3b). In sarcopterygians, TRPM4 was found in basally radiating representatives (the coelacanth and two lungfishes), in amphibians, in mammals, and some sauropsids (squamates, chelonian, crocodilian) but was lacking in birds suggesting a specific loss of TRPM4 in this group (Figure S2e). Two TRPM4 paralogs were retrieved in a turtle, *Chelonia mydas*, clustering together in the phylogeny, suggesting a species-specific duplication. TRPM4 is present in basal actinopterygians (cladistian reedfish and holostean spotted gar) and teleosts. Most teleosts possess two TRPM4 paralogs located on separate chromosomes, which cluster into two clades TRPM4a and TRPM4b, in agreement with their possible origin via teleost 3R. However species-specific independent losses of TRPM4a were observed in some species, such as in fugu, turbot, and tilapia (see Figure S2g). In contrast, some teleosts possess additional TRPM4 paralogs located adjacently on the same chromosome, which cluster in a species-specific manner in the phylogenetic tree. This is the case for three TRPM4a in the pike, and two TRPM4b in the herring, three TRPM4b in the zebrafish, two TRPM4b in the cod (Figures S2f,g and S3b). This suggests that independent species-specific gene duplications of TRPM4a and of TRPM4b occurred repeatedly in the teleost lineage. Three TRPM4a are also present in salmonids, Atlantic salmon and rainbow trout. Synteny analysis is described below.

##### Phylogeny of TRPM5

TRPM5 was found in all gnathostomes investigated (Figure S3b), except in the anuran amphibians such as the Leishan spiny toad and *Xenopus tropicalis*. Using the coelacanth TRPM5 sequence we further searched by BLAST for TRPM5 sequence in amphibians, and found TRPM5 orthologous sequences in gymnophiona, *Microcaecilia unicolor and Rhinatrema bivittatum*, as shown by the phylogeny analysis. This suggests that the loss of TRPM5 gene specifically occurred in an anuran ancestor after the separation from gymnophiona (see Figure S2e). A single TRPM5 gene was found in all teleosts investigated, as in non-teleost actinopterygians, the reedfish and the spotted gar. This suggests that the loss of the other TRPM5 3R-ohnolog occurred shortly after 3R, in an ancestral teleost, which is supported by the synteny analysis. Two TRPM5 genes were retrieved in salmonids (Atlantic salmon and rainbow trout), likely resulting from 4R as supported by the synteny analysis. Sequences from cyclostomes, lampreys (*Petromyzon marinus*, *Lethenteron reissneri)* and myxine (*Myxine glutinosa*) previously annotated as TRPM5, TRPM4like or not annotated in NCBI, cluster at the base of the gnathostome TRPM4 and TRPM5 clades, as also observed in the global phylogeny (Fig. 2). This further supports the presence of TRPM from the β clade in cyclostomes, in contrast to the previous report by Himmel et al. (2020). We propose to name these sequences TRPM4/5. This suggests that the duplication that led to TRPM4 and TRPM5 may have arisen in the gnathostome ancestor after it had diverged from the cyclostome lineage, in agreement with the paralogon study that assigned it to the gnathostome 2R.

#### Conserved synteny of the TRPM2, 8 genomic region

The genomic region of the reedfish TRPM2 was used as a template, and nine neighboring genes were investigated (Figure S4c) (see Table S2 for references and location of TRPM and neighboring genes). The two copies of TRPM2 in the reedfish are syntenic which supports a cladistian-specific local gene duplication, in agreement with the phylogenetic analysis. The duplicated TRPM8 genes in *Xenopus tropicalis* are located in tandem, indicating local gene duplication which supports the phylogeny. TRPM8, which is present only in sarcopterygians, is syntenic with TRPM2 in *Xenopus tropicalis*, while TRPM2, together with one of the neighboring genes, has been translocated to another chromosome in amniotes, as shown in bird (duck) and mammal (human) (Figure S4c). The synteny of TRPM2 and TRPM8 in amphibian is in agreement with a possible origin from a local gene duplication of an ancestral TRPM2/8 gene. The TRPM8 gene is missing in chondrichthyans and actinopterygians. Some of the neighboring genes were found to have duplicates on other chromosomes in teleosts, in agreement with the 3R duplication of this genomic region. A single 3R TRPM2 ohnolog has been conserved in all teleosts, suggesting an early loss of the other ohnolog after the 3R. The two TRPM2 genes present in pike are located on the same chromosome in tandem position, which supports origin from species-specific local gene duplication, in agreement with the phylogeny analysis (Figure S3b). Some of the neighboring genes were further duplicated in salmon, as four ohnologs or three ohnologs on separate chromosomes, in agreement with 4R. Despite 4R, a single TRPM2 was found in salmonids, indicating loss of one of the duplicated 4R-TRPM2 ohnologs. In the current lamprey genome assembly, the gene annotated TRPM2 is located in a genomic region encompassing two syntenic neighboring genes (Figure S4c), supporting the orthology with gnathostome TRPM2/8, in agreement with the phylogeny analysis (Figure S3b).

#### Conserved synteny of the TRPM4 genomic region

The genomic region of the reedfish was used as template. Eight neighboring genes were investigated (Figure S4d) (see Table S2 for references and location of TRPM and neighboring genes). This synteny was largely conserved across gnathostomes, supporting the orthology of TRPM4. Some neighboring genes were retrieved in the current lamprey genome assembly, but no TRPM4 ortholog. Some neighboring genes were found in duplicates on separate chromosomes in teleosts, in agreement with the 3R duplication of this genomic region. Both 3R ohnologs of TRPM4, named TRPM4a and TRPM4b, were conserved in most teleosts. Repeated independent losses of TRPM4a may have occurred in some species, such as fugu and turbot. In contrast, multiple paralogs of TRPM4b (herring, zebrafish, and cod) or of TRPM4a (pike) are present in tandem position, supporting their likely origin from species-specific local gene duplication as suggested by the phylogeny analysis. In the herring, a TRPM4b duplicate is located on another chromosome, suggesting in this species a translocation after local duplication. In the Atlantic salmon, one of the neighboring genes is present as three ohnologs on separate chromosomes, in agreement with the 4R-duplication of this genomic region. Four TRPM4 paralogs are found in the Atlantic salmon: three TRPM4aα, located in tandem in the synteny analysis showing that they arose by local gene duplications as in the pike; and one TRPM4bα, while the other potential 4R-ohnologs would have been lost.

#### Conserved synteny of the TRPM5 genomic region

TRPM5 genomic region of the reedfish was used as the template with seven neighboring genes (Figure S4e) (see Table S2 for references and location of TRPM and neighboring genes). The synteny of the genomic region is conserved across gnathostomes supporting the orthology of TRPM5. In the lamprey, the TRPM annotated as TRPM5 in NCBI is located in a region including three of these neighboring genes. Some of the neighboring genes in teleosts have ohnologs on separate chromosomes in agreement with the 3R. However, TRPM5 was retained as a single ohnolog in teleosts including basal representatives (eel and bonytongue) supporting the early loss of the other 3R ohnolog after the 3R. This genomic region was further duplicated by 4R in salmonids, as shown by the presence of four ohnologs and three ohnologs of some neighboring genes in Atlantic salmon. The two 4R ohnologs of TRPM5 are retained in Atlantic salmon.

#### Paralogon for vertebrate TRPM2 and TRPM8 genes

The TRPM2 and TRPM8 genes are located on the same chromosome in opossum and *Xenopus tropicalis*, but are on separate chromosomes in human and chicken (Fig. 3b) (see Table S3 for references and location of TRPM and neighboring genes). Although the phylogeny of the genes suggests origin by duplication of a common ancestral gene before the radiation of gnathostomes (Fig. 2), only the sarcopterygian representatives display the TRPM8 gene. Several syntenic genes were found to belong to families with members on three other chromosome blocks that radiated at the origin of gnathostomes, and their species distribution is consistent with this (see the phylogeny of the neighboring genes Figure S5b). Thus, this region seems to belong to a four-membered paralogon (Fig. 3b). However, only one neighboring family is a full quartet, namely HDAC. Five other families have three members, ie IGF2BP, ARL4, DIP2, PFK, and MARCH4, that together are consistent with an ancestral quartet. The quartet suggests that additional TRPM2/TRPM8 ohnologs could have been expected after 1R and 2R, but apparently have been lost.

#### Paralogon for vertebrate TRPM4 and TRPM5 genes

The TRPM4 and TRPM5 genes are located on two separate chromosomes where each one has members from a limited set of adjacent gene families (Fig. 3c) (see Table S3 for references and location of TRPM and neighboring genes). Taking together the information from the eight sampled species, there are four syntenic families that are full quartets, namely SYT3,6,9,10, MYBP, SYT1,2,5,8, and PPFIA. Among these, MYBP is missing only one member in one species. The other three are missing a few more. In addition, two families are trios, KCNJ8,11,11L, and RIC8. All of the neighboring gene families have sequence-based phylogenies (Figure S5c) and species distribution in line with duplications in the early evolution of gnathostomes. Comparison of the gnathostome chromosomes with the recent lamprey genome assembly (Marlétaz et al. 2024) suggests that TRPM4 and TRPM5 resulted from the gnathostome 2R event and the 1R duplicate would have been lost. However, the information for these chromosomes is rather incomplete in Marlétaz et al. (2024), in that only 4 out of the 6 expected lamprey chromosomes were identified, only 1 out 4 is considered in *Xenopus tropicalis,* and one chromosome is missing in chicken and spotted gar. Indeed, most of this part of the TRPM4 chromosome in chicken is missing in the genome assembly, including TRPM4 itself which appears to have been lost in the common ancestor of birds. Also, some of the TRPM4 neighbors in spotted gar are on scaffolds. However, we did find TRPM4 syntenic regions in both *Xenopus tropicalis* and spotted gar as shown in Fig. 3c. By combining this information with new information in the NCBI database, including the new spotted gar genome assembly with chromosome assignments (rather than linkage groups) as well as the chicken genome assembly GRCg7w, additional pieces fall into place, albeit some information is missing in these datasets too, but nevertheless confirming the existence of a quartet of ohnolog genomic regions with the TRPM4 and TRPM5 chromosomes as closest relatives.

### Evolution of structural domains of TRPM/TRPS channels

#### Melastatin domain

The aminoterminal cytoplasmic melastatin region was found to be present in all members of the TRPM and TRPS families. Furthermore, this region contains all four melastatin domains in all of the members, ie MHR1/2, MHR3, and MHR4 (Fig. 1). The melastatin region thereby constitutes a consistent characteristic feature of the TRPM and TRPS families. It appears to have a complex history and the MHR1/2 part of the melastatin region has been described in bacteria as a so called SLOG domain (Burroughs et al. 2015), also identified in archaea (Mayaka et al. 2019). However, the TRP ion channel domain has not been found in bacteria (Burroughs et al. 2015; Himmel and Cox 2020). Thus, the fusion of the SLOG region with the TRP ion channel domain probably happened in a rather early eukaryote genome (Burroughs et al. 2015), most likely prior to the origin of multicellularity. Also MHR3 and MHR4 with the ankyrin repeats identified in (Burroughs et al. 2015) probably joined before the metazoan radiation.

#### Aminoterminal Cysteine-rich domain

The most aminoterminal region has been described as Cys-rich in human TRPM (Fig. 1) due to its several cysteine residues (Burroughs et al. 2015). In human TRPM, apart from TRPM5, the aminoterminal region preceding the conserved amino acid motif KHT consists of 46 to 128 residues with 3 to 7 cysteines. Across a panel of gnathostomes, the range is 3 to 9 cysteines with strong conservation of the core set within each TRPM subtype (data not shown). In amniotes, TRPM5 has a much shorter aminoterminal domain. This region has only two Cys in sauropsids such as turtle and chicken, as well as in some mammals such as wombat and mouse, while it is even shorter in human TRPM5 with no Cys at all. The region is longer for TRPM5 in non-amniote gnathostomes and has several Cys residues, up to seven in spotted gar.

We could also identify this Cys-rich segment in non-vertebrate species, such as for cnidarian TRPMα, TRPMβ, and TRPMγ or for the single *Drosophila melanogaster* TRPM (TRPMα). In some cases, no Cys are present in the aminoterminal region such as in TRPMβlike from a mollusk (*Pecten maximus*) or from amphioxus, while Cys is present in TRPMβlike from an annelid (*Lamellibrachia satsuma*). This indicates that the loss of the Cys-rich domain occurred not only in some amniote TRPM5, but also in TRPM from other lineages. There is no Cys-rich region in any of the TRPS analyzed. This suggests that the Cys-rich domain was added to the ancestral TRPM gene after the duplication that led to TRPM and TRPS (Figure S2a). The conservation of this Cys-rich domain in most TRPM, from cnidarians to vertebrates, supports significant functional roles. However, to the best of our knowledge, the exact roles of the Cys residues in the aminoterminal are still unexplored.

#### Ion channel domain

The ion channel domain (Fig. 1) is generally highly conserved across the entire TRP superfamily as well as across species for each of the vertebrate TRPM1 to 8 subtypes. Some variation is observed for the pore loop between transmembrane regions S5 and S6 for some of the subtypes, but all vertebrate sequences display two cysteines presumed to form a disulfide loop. These two cysteines are important for channel function as demonstrated by the consequences of Cys mutation for human TRPM2 (Mei et al. 2006) and mouse TRPM8 (Dragoni et al. 2006). The stretch between the two cysteines is in the range 9 to 17 residues, except that TRPM6 is clearly shorter with only five. Non-vertebrate bilaterians have these two cysteines in TRPMα, β, and γ from various lineages except in cnidarian sequences (data not shown). We also observed two cysteines in TRPS sequences. This suggests that the role of the disulfide loop of the pore region is an ancestral characteristic of the TRPS/TRPM channels. Their potential loss in cnidarian TRPM would deserve investigation of the consequences on the channel function.

#### NUDT9H domain

The cytoplasmic region in the carboxyterminal part of the ion channel domain displays more organizational variation than the aminoterminal region. A NUDT9 homology domain, belonging to the Nudix family, has been found in some members of the TRPM family. The most carboxyterminal region of TRPM2 in vertebrates has a stretch of some 300 amino acids with approximately 50% sequence identity to the protein NUDT9, hence this region was named NUDT9H where H stands for homology domain. NUDT9 is a mitochondrial protein encoded in the nucleus that belongs to a large and ancient protein family whose members share a Nudix hydrolase domain of approximately 150 amino acids acting as an ADPR pyrophosphatase (Carreras-Puigvert et al. 2017; Srouji et al. 2017). This domain is named Nudix because it is present in hydrolases that cleave nucleoside diphosphates linked to “any moiety” (denoted as X). ADPR binding to NUDT9H was early reported to have some catalytic activity and open the TRPM2 channel (Perraud et al. 2001). However, the protein was later found to lack catalytic activity (Iordanov et al. 2016) due to mutations which would have occurred before the gnathostome radiation (Iordanov et al. 2019). Mere binding of ADPR to NUDT9H seems to be sufficient for channel opening (Tóth et al. 2015). More recently cADPR (cyclic ADPR) binding to NUDT9H has been shown to activate TRPM2 (Yu et al. 2019) and the three-dimensional structure has been determined (Wang et al. 2018). Among the vertebrate TRPM members, NUDT9H is only present in TRPM2 (Fig. 1). The NUDT9H domain is also present in the closest relative of the TRPM family, TRPS, indicating that it was most likely present in the TRPM/TRPS ancestor as proposed by Himmel et al. (2020) and supported by our study (see Figure S2a). Some other authors previously suggested that the NUDT9H domain was an ancestral feature of TRPM and was lost independently in the ancestor of the vertebrate TRPM1/3/6/7 subfamily and in the ancestor of vertebrate TRPM4/5/8 (Mederos and Schnitzler et al. 2008). As our phylogenetic analysis clearly shows that gnathostome TRPM2 and 8 are most closely related to each other, independent NUDT9H losses would have happened in TRPM8 and the ancestor of TRPM4/5 (see Figure S2d). In cyclostomes, a TRPM2/8 gene was identified that branches off prior to the divergence of TRPM2 and TRPM8 in gnathostomes, meaning that the duplication took place in the gnathostome ancestor (Figure S3b). The cyclostome TRPM2/8 lacks NUDT9H, indicating a loss of this domain. Apart from vertebrates, we further investigated the presence of the NUDT9H domain in metazoan TRPMα, β, βlike, and γ sequences. This led to the conclusion that the NUDT9H domain has been lost in total at least six times independently: in bilaterian ancestor of TRPMα (NUDT9H being still present in cnidarian TRPMα), in metazoan ancestors of TRPMβlike and of TRPMγ, as well as three times in the TRPMβ lineage, namely both in the ancestor of TRPM4/M5 and in the cyclostome TRPM2/8, and in the gnathostome TRPM8 (see Figure S2a–d). In fact, it has only been retained in TRPS, cnidarian TRPMα, non-vertebrate TRPMβ, and gnathostome TRPM2.

The NUDT9H domain that remains today in gnathostome TRPM2 acts more as sensor by binding certain metabolites without catalysis (Sander et al. 2022). Iordanov and coworkers demonstrated that human TRPM2 is activated by ADPR and contains a NUDT9H domain homologous to ADPR phosphohydrolases, but is catalytically inactive due to mutations in the conserved Nudix box sequence (Iordanov et al. 2019). In contrast, the NUDT9H domain of the cnidarian *Nematostella vectensis* “TRPM2” (TRPMβ in our phylogeny-based nomenclature) and of the “TRPM2” of the choanoflagellate *Salpingoeca rosetta,* possess an ADRPase enzymatic activity (Iordanov et al. 2019). The hydrolysis of the activating ligand would represent a downregulating mechanism of the channel gating. By comparing the TRPM NUDT9H sequences from choanoflagellates, and representatives of various metazoans including placozoan, cnidarian (anemone), annelid, echinoderm, cephalochordate, urochordate, and vertebrates (chondrichthyan, teleost, amphibian, birds, and human) Iordanov et al. (2019) further showed the conservation of amino acids critical for ADPR hydrolysis in TRPM from unicellular flagellates and from non-vertebrate metazoans, and their loss in gnathostome TRPM2. The authors (Iordanov et al. 2019) also revealed that amino acid substitutions in the pore sequence were responsible for the intrinsic mechanism of inactivation of gnathostome TRPM2. They proposed that catalytic activity and pore stability were lost simultaneously in gnathostome TRPM2 channels. In the present study we analyzed a large set of non-vertebrate metazoan TRPMβ sequences, including cnidarians (hydra, anemone, corals), mollusks, echinoderms, hemichordates, cephalochordates, urochordates, and vertebrate TRPM2 sequences including cyclostomes, chondrichthyan, basal sarcopterygians (lungfish and coelacanth), and various tetrapods.

Our sequence comparison further supported the conservation of key amino-acids for ADPRase activity in non-vertebrate TRPM and their loss in gnathostome TRPM2 (see Figure S6a). In cyclostomes (lamprey and hagfish), the partial sequences of TRPM2/8 available in current genome databases did not allow us to examine the corresponding sequence. Concerning the sequence responsible for pore stability or inactivation, our analysis supported the findings by Iordanov et al. (2019) of conservation of key amino acid responsible for intrinsic inactivation in non-vertebrate TRPMβ, and their mutation in gnathostome TRPM2 (see Figure S6b). Furthermore, we also observed key amino acid substitutions in the corresponding sequence in cyclostomes (see Figure S6b), allowing us to trace back to the vertebrate ancestor the presence of the TRPM2 intrinsic inactivation mechanism. This further supports Iordanov's hypothesis that the inactivation of TRPM would be performed by ligand hydrolysis in non-vertebrates, while by pore intrinsic inactivation in vertebrates.

Since the NUDT9H domain is also present in TRPS, the sister clade of TRPM, we examined the amino acids required for ADPR hydrolysis and found that they were not conserved in any of the species investigated (mollusks: *Octopus vulgaris, Octopus bimaculoides, Crassostrea gigas, Pecten maximus, Pomacea canaliculate, Aplysia californica,* nematod *C. elegans,* cephalochordates *Branchiostoma floridae, Branchiostoma belcheri*) (see Figure S6a). This suggests that, differently from TRPMβ, the NUDT9H catalytic activity of TRPS was lost early in a metazoan ancestor. We also examined the pore sequence and found substitutions of key amino acids involved in TRPM pore stability (see Figure S6b), suggesting a potential intrinsic inactivation mechanism for TRPS as for vertebrate TRPM2. These data suggest that NUDT9H catalysis activity and pore stability were lost at least twice, namely in an ancestral metazoan TRPS and in an ancestral vertebrate TRPM2.

#### TRPM6,7 kinase domain

TRPM6 and 7 have been found to have an atypical serine/threonine kinase domain at the carboxy terminus (Nadler et al. 2001; Runnels et al. 2001) (Fig. 1) related to the family of alpha kinases, named so because of their ability to phosphorylate Ser/Thr residues in alpha helices. The TRPM kinase domain is capable of both autophosphorylation and phosphorylation of other cytoplasmic proteins (Clark et al. 2008). The kinase domains of TRPM6 and TRPM7 are cleaved and transported to the nucleus where they phosphorylate histones and influence gene expression (Desai et al. 2012; Krapivinsky et al. 2014, 2017). The channel and kinase functions appear to be independent.

**Figure 4:**
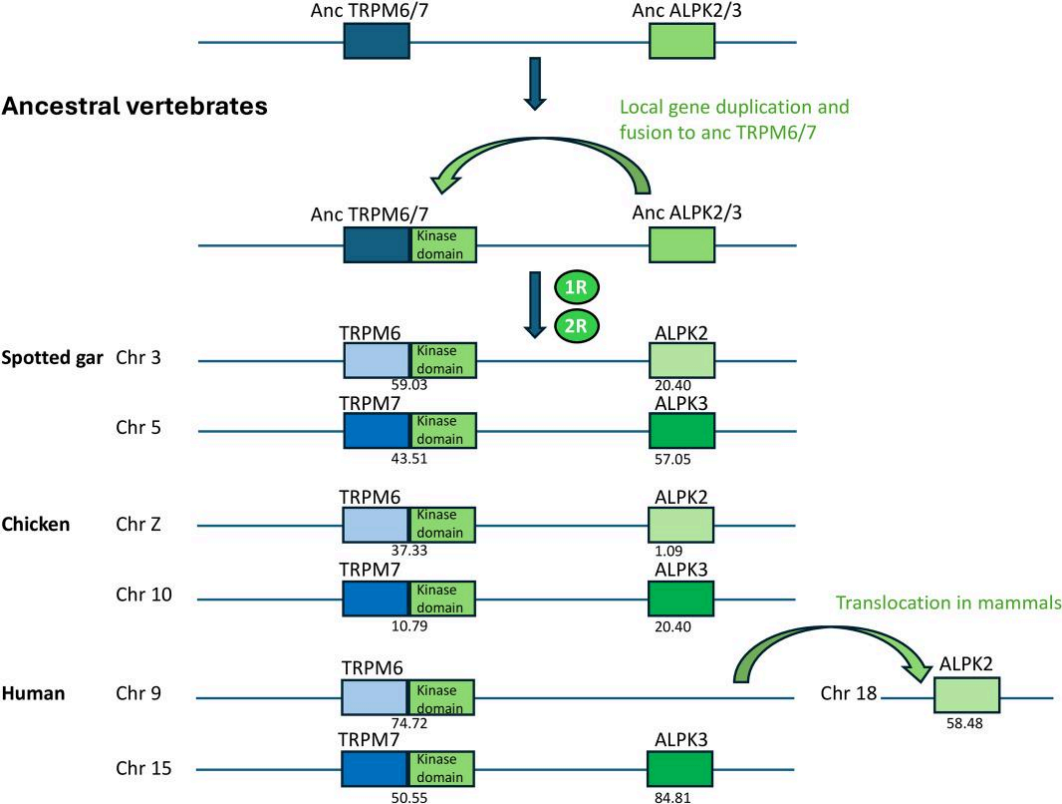
Proposed evolutionary scenario for the addition of a kinase domain to TRPM6/7. Vertebrate TRPM6 and TRPM7 are the only TRPM members with a kinase domain. The kinase domain of the ancestor of TRPM6 and TRPM7 most likely came from a local gene duplication of an ancestral kinase ALPK2/ALPK3 gene (see also Figure S7), followed by the fusion to the ancestral TRPM6/7 located on the same chromosome. Duplications via early vertebrate genome doublings (1R/2R) and gene losses led in gnathostomes to TRPM6 located on the same chromosome as ALPK2 and to TRPM7 on the same chromosome as ALPK3. A translocation of ALPK2 to another chromosome occurred later in the mammalian lineage.

The C-terminal kinase domain is specific to TRPM6 and 7 (Fig. 1). Our phylogenetic analysis of a large set of TRPM and TRPS metazoan sequences further supports that the addition of the kinase domain is a unique event that occurred in the vertebrate TRPM ancestor of TRPM6 and 7. The kinase domain of TRPM6 and TRPM7 is related to the three vertebrate alpha protein kinases ALPK1, 2, and 3 (Middelbeek et al. 2010). A phylogenetic analysis of these domains shows that TRPM6 and TRPM7 kinase domains are most similar to one another (Figure S7), supporting that the kinase domain was added to their common ancestor. Furthermore, they are most closely related to ALPK2 and ALPK3 which in turn are more similar to each other (Figure S7), suggesting that the kinase domain of the TRPM6/7 ancestor was a duplicate of the ALPK2/3 ancestor (Fig. 4). Interestingly, TRPM7 and ALPK3 are both on the same chromosome in human (chromosome 15) as well as in all other bony vertebrates that we have investigated. Although the genes are presently far apart on their chromosome, it is likely that the kinase domain of TRPM6/7 was a gene duplicate of the ALPK2/3 ancestor on the same chromosome (Fig. 4). Subsequently, the chromosome was duplicated in the two vertebrate and gnathostome 1R and 2R events. Indeed, ALPK2 is on the same chromosome as TRPM6 in chicken and spotted gar, but seems to have been translocated in mammals, as observed in platypus, opossum, and human (Fig. 4). This evolutionary scenario is supported by the exon-intron organization of the kinase domains. TRPM6 and 7 have three introns in the gene region encoding the kinase domain and these are located at the same positions as for ALPK2 and ALPK3. Furthermore, these three introns are in the same reading frame in these four sequences. Thus, the kinase domain was likely added to the TRPM6/7 ancestor as a gene duplication of the ALPK2/3 ancestor, not a retro-transcript. The more distantly related ALPK1 on the other hand has different intron positions. We also observed that the kinase domain has been conserved in all duplicated TRPM6 and TRPM7 paralogs, whether they were issued from whole genome duplication (3R or 4R) or from lineage or species-specific gene duplication.

### Micro-RNA genes in TRPM1 and TRPM3 introns

The micro-RNA gene mir-211 present in an intron of human TRPM1 (Lee et al. 2016), was found in all gnathostome TRPM1 genes that we investigated in detail, including chondrichthyans, actinopterygians, and sarcopterygians. Likewise, the related gene mir-204 present in the corresponding intron of human TRPM3 gene (Lee et al. 2016), the closest paralog of TRPM1, was also retrieved in all gnathostome TRPM3 genes.

**Figure 5:**
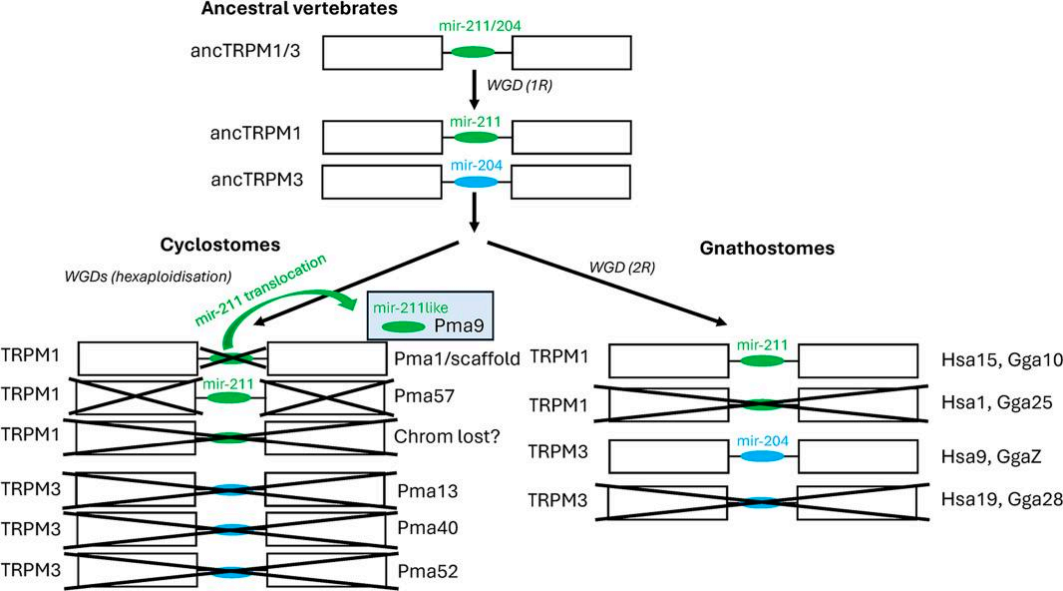
Proposed evolutionary scenario for the presence of mir-211/204 in an intron of TRPM1/3. Vertebrate TRPM1 and TRPM3 are the only TRPM members with a mir sequence in an intronic region. An ancestral mir-211/204 would have been present in the intron of an ancestral TRPM1/3. Duplication via the first vertebrate genome doubling (1R) would have led to TRPM1 with mir-211 in an intron and TRPM3 with mir-204 in an intron (see also Figure S8). As mir-204 sequences are found only in gnathostomes and not in cyclostomes, an alternative hypothesis (not shown) would be that the mutations leading from mir-211 to mir-204 would have occurred in gnathostomes after the second genome doubling (2R). In gnathostomes, the 2R generated no surviving duplicates. In cyclostomes, despite the hexaploidization, multiple gene and domain losses led to a single TRPM1 with no associated mir, a separate mir-211 and a possible translocated additional mir-211like gene.

This reveals that the duplication of the ancestral mir-204/211 into mir-204 and mir-211 occurred in 1R in the vertebrate ancestor together with the duplication of TRPM1 and TRPM3 (Fig. 5), refuting previous assumption of the emergence of mir-211 in mammals (Barbato et al. 2017). Alignment and phylogeny analysis of vertebrate mir-204 and mir-211 clearly cluster 204 and 211 sequences in two separate clades (Figure S8a,b). No additional duplicates survived the 2R event, neither TRPM nor mir (Fig. 5). Among actinopterygians, in teleosts, 3R duplicated TRPM1 into TRPM1a and b, and both ohnologs retained their duplicated mir-211 (mir211a and mir211b). Only a single TRPM3 ohnolog was retained after 3R together with its mir-204 (mir-204a). Salmonid 4R generated up to three TRPM1 with three corresponding mir-211, and two TRPM3 with the two corresponding mir204 (Figure S8a,b). All these data support the conservation of mir-211 and 204 in the intronic sequences of TRPM1 and TRPM3 respectively, across gnathostome radiation.

It is well established that TRPM1 plays a role in vision (Shen et al. 2009) and TRPM3 is also expressed in the retina (Behrendt 2022). Strikingly, both miR-204 and miR-211 are also expressed in various regions of the eye (Barbato et al. 2017), suggesting a potential regulatory link between these miR and TRPM channels. In medaka, knockdown of mir-204 results in progressive alteration and death of photoreceptor cells, suggesting a direct function of miR-204 in differentiation, maintenance and function of these cells (Conte et al. 2015). In mice, mir-211 knockout leads to a progressive cone dystrophy accompanied by significant alterations in visual function (Barbato et al. 2017). Deletion of mir-204 and mir-211 in mouse and medaka, resulted in a deleterious cell clearance program associated with a progressive retinal degeneration, supporting a role of miR-204 and miR-211 in maintaining the retinal pigment epithelium function (Intartaglia et al. 2021; Du et al. 2024). These findings suggest that the functions of TRPM1 and TRPM3 in the retina may be closely linked to miR-204 and miR-211 regulatory pathways. Similar to TRPM channels, miRs have also been implicated in cancer (Mazar et al. 2010). Mazar et al. (2010) proposed that the tumor-suppressor activities of microphthalmia-associated transcription factor and/or TRPM1 may be partially mediated through miR-211, which negatively regulates the KCNMA1 transcript (a calcium ion-regulated potassium channel protein), known to be overexpressed in metastatic melanoma, prostate cancer, and glioma. In breast cancer, miR-204/211 promote cell proliferation, at least in breast cancer cell lines MCF-7 and MDA-MB-231, by downregulating tumor suppressor genes (Lee et al. 2016). In melanoma, evidence also suggests that miR-211 acts as a suppressor of cancer cell invasion (Levy et al. 2010). Like the pleiotropic effects of TRPM channels, miRs exhibit diverse biological functions. Huang et al. (2010) identified miR-204 and miR-211 as negative regulators of osteoblast differentiation in mesenchymal progenitor cells and bone marrow stromal cell. Additionally, Liu et al. (2021) highlighted the role of miR-204 in cardiovascular and renal diseases. In zebrafish larvae, miR-204-3-3p (which corresponds to mir-211b according to our phylogeny analyses of mir-204 and mir-211 across vertebrates) responds to light and regulates the expression of cryptochrome genes (Wang et al. 2025). Despite growing evidence, the complex interactions between miRs and TRPM channels remain largely unexplored.

By BLAST we also identified mir-204/211-homologous sequences in cyclostome genomes: in two lampreys (*Petromyzon marinus* and *Lethenteron reissneri*) and two hagfishes (*Myxine glutinosa* and *Eptatretus burgeri*). These sequences were more closely related to gnathostome mir-211 than mir-204, according to our alignment, and cyclostome mir sequences clustered with gnathostome mir-211 in our phylogeny analysis (Figure S8a,b). Based on these results, we recommend to rename cyclostome mir as 211 instead of mir-204, which was the name previously given by Heimberg et al. (2010) and the miR database. As mir-204 and mir-211 appear to have arisen from their common ancestor in 1R, this suggests that the mir-204 ohnolog has been lost in cyclostomes (Fig. 5). No information is available on the putative function of TRPM1 nor mir-211 in cyclostomes. In agreement with Heimberg et al. (2010) we did not retrieve by blast any mir-204/211 sequence in non-vertebrate genomes, including urochordates, cephalochordates, and protostomes. Altogether, our data suggest that mir-211/204 ancestor has an ancient origin in early vertebrates before the emergence of cyclostomes.

Remarkably, in cyclostomes, mir-211 is not located inside a TRPM gene, but is located on a different chromosome/scaffold/contig than TRPM1, as observed in the lampreys, *P. marinus* and *L. reissneri*, and the hagfishes, *M. glutinosa* and *E. burgeri* (Fig. 5). In cyclostomes, current genomic data suggest that a genome hexaploidization event followed after 1R (Marlétaz et al. 2024; Yu et al. 2024). Notably, the chromosome carrying mir-211 belongs to the *P. marinus* TRPM1 hexaparalogon, supported by eight neighboring genes that have family members on the four chromosomes in this paralogon in gnathostomes (not shown). To assess the presence or absence of mir inside an intronic region of TRPM in cyclostomes, we further performed a local Blast of mir-211 on a genomic region encompassing TRPM1 in lampreys and hagfishes. We could not retrieve any mir sequence in the TRPM1 genomic region. However, while blasting *P. marinus* mir-211 on *P. marinus* genome on NCBI, we retrieved an additional homologous small non-annotated sequence located on *P. marinus* chromosome 9, a chromosome that does not belong to the TRPM1 hexaparalogon. Our alignment revealed that this sequence shared typical feature of mir-211 (Figure S8a), allowing us to suggest that it may have arisen from a translocation of one of the putative mir-211 paralogs resulting from the cyclostome hexaploidization. We have named it mir-211like. Like the full-length mir-211, this small sequence encompasses the seed region suggested to bind the 3' UTR of cryptochrome in zebrafish (Wang et al. 2025).

The other two TRPM members of the α clade, TRPM6 and TRPM7, have not been reported to contain any micro-RNA genes, which is supported by our study. The evolutionary scenario of TRPM1/3 and mir-204/211 suggests that an ancestral mir-211/204 was inserted in the intron of the ancestor of TRPM1 and TRPM3 in a vertebrate ancestor (Fig. 5). Subsequently, the first whole-genome duplication (WGD) event in early vertebrates (1R) likely led to the duplication of the chromosomal region containing the ancestral TRPM1/3-mir gene, resulting in ancestral TRPM1/mir-211 and ancestral TRPM3/mir-204. The second round of gnathostome whole-genome duplication (2R), followed by gene losses, would have then resulted in the current configuration: one TRPM1/mir-211 and one TRPM3/mir-204 (Fig. 5). In cyclostomes, despite the hexaploidization, a single TRPM1 and no TRPM3 was retrieved, indicating multiple gene losses. Furthermore, exons and intron losses, as well as intron translocation would have led to the presence of TRPM1 without mir-211 and isolated mir-211 and mir-211like (Fig. 5).

### Overview of the evolution of the TRPM/TRPS family

**Figure 6:**
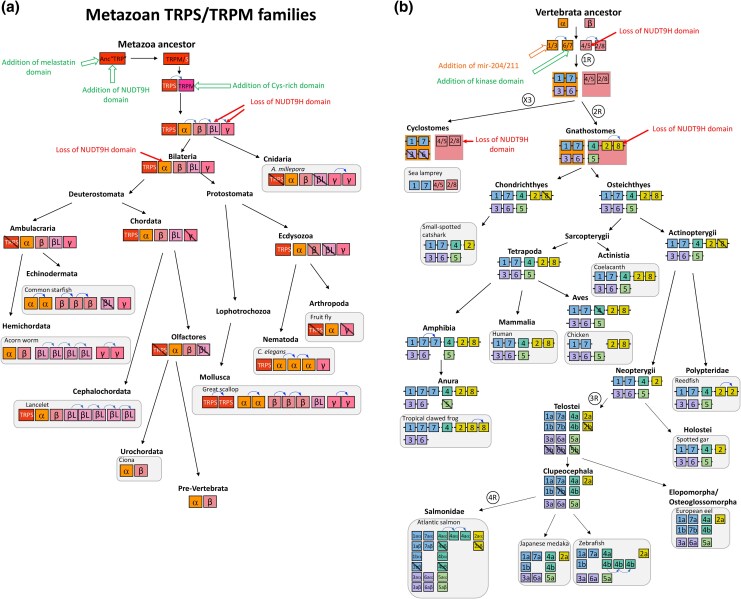
Proposed evolutionary scenario for the TRPS/TRPM family. a) Evolutionary scenario of TRPM and TRPS in non-vertebrate metazoans. Addition of the typical melastatin domain to an ancestral TRP led to the common ancestor of both TRPM and TRPS. NUDT9H domain was also added. This gene duplicated (green arrow) into TRPM and TRPS. A cysteine-rich domain was added to the TRPM ancestor gene. TRPS remained as a single gene throughout metazoans, with rare exceptions, such as a specific gene duplication in mollusks, or independent gene losses in Cnidaria, Insecta, Ambulacraria, and Olfactores. In contrast, serial gene duplications before the split of Bilateria and Cnidaria led to four TRPM types (α, β, βlike, and γ). The NUDT9H domain was lost in TRPMβlike and γ, as well as in bilaterian TRPMα. Additional gene duplications expanded the repertoire of TRPM in a species-specific manner, as illustrated with eight TRPM in mollusks and hemichordates, and seven in cephalochordates. Conversely, specific gene losses of various TRPM types led for instance to a single TRPM (α) in Drosophila, and only two TRPM (α and β) in urochordates and pre-vertebrates. The loss of TRPMγ in Chordata and of TRPS and TRPMβlike in Olfactores led to the inheritance of only TRPMα and TRPMβ by vertebrate ancestors. For detailed evolutionary scenario see also Figure S2a–c. b). Evolutionary scenario of TRPM in vertebrates. All the TRPM types present in extant vertebrates originated from the metazoan TRPMα and β types. Ancestral TRPM 1/3 and 6/7 genes resulted from a local gene duplication (blue arrow) of TRPMα. An ancestral mir-211/204 was inserted in an intron of the ancestral TRPM1/3, and a kinase domain was added to the carboxyterminal ancestral TRPM6/7. Ancestral TRPM 4/5 and 2/8 genes resulted from a local gene duplication of TRPMβ. Vertebrate whole-genome duplication 1R, followed by gnathostome 2R and local duplication of TRPM2/8 as well as gene losses (not shown) resulted in the TRPM 1 to 8 present in gnathostomes and conserved in mammals including human. In cyclostomes, hexaploidization that followed 1R and massive gene losses led to four TRPM (TRPM1, 7, 2/8, and 4/5). TRPM8 is present only in sarcopterygians, and the sequence-based phylogeny suggests that it was lost in chondrichthyans and actinopterygians. The other TRPM (1 to 7) have been retained throughout gnathostome radiation, with a few exceptions such as the loss of TRPM4 in birds and of TRPM5 in anuran amphibians. Some species-specific local gene duplications occurred, such as for TRPM2 in Polypteridae, TRPM7 in amphibians and TRPM8 in *Xenopus tropicalis*. The teleost-specific whole genome duplication (3R), followed by ohnolog retention or losses, further diversified the TRPM repertoire, along with species-specific gene duplication such as for TRPM4b in zebrafish. Additional whole genome duplication in salmonids (4R) followed by gene losses or specific local duplication led to a total of 16 TRPM members in Atlantic salmon. For detailed evolutionary scenario see also Figure S2d–g.

**Figure 7:**
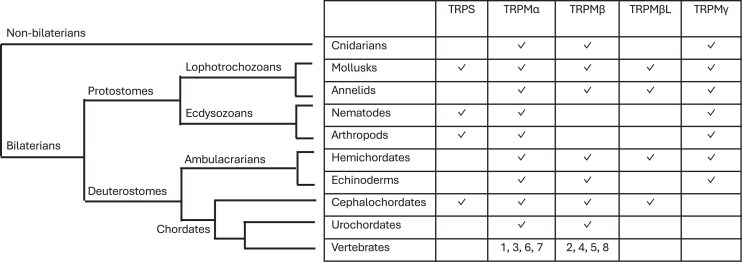
TRPS and TRPM types and taxa. Summary of the main TRPS and TRPM types evidenced in the present study with their presence or absence in the main metazoan taxa.

The identification of TRPM/TRPS family members across metazoans, followed by sequence comparison, phylogeny and synteny analyses, allows us to present a comprehensive evolutionary scenario, shown in detail in Figure S2 and summarized in Fig. 6a,b. The presence/absence of TRPS and TRPM types in the various metazoan taxa is shown in Fig. 7. In the large TRP superfamily, TRPS is the closest relative of TRPM. Both TRPS and TRPM share the presence of the typical melastatin region and would have arisen by duplication of a TRPS/TRPM ancestral gene, as proposed by Himmel et al. (2020) (Fig. 6a). A NUDT9H domain, belonging to the Nudix family, would also have been part of this ancestor. NUDT9H has been retained in TRPS but independently lost in various TRPM types during metazoan evolution. For those TRPM duplications that were not part of chromosome/genome duplications, it could be that the NUDT9H exons were lost because they were not included in the duplicated unit, as this domain is at the carboxyterminal end of the protein, ie the 3' and of the gene. For instance, TRPM8 may never have had a NUDT9H domain in case this part was not duplicated from the TRPM2/8 gene. As compared to TRPS, the addition of a cysteine-rich domain specifically occurred in the ancestor of the TRPM lineage. This domain is present in all metazoan TRPM types, except in some amniote TRPM5.

TRPS appears to have been lost in a cnidarian ancestor but was conserved in various bilaterian lineages. Among protostomes, it is present in some ecdysozoans (arachnids and nematodes) but was lost in insects (drosophila); in lophotrochozoans it is present in mollusks, while it is missing in annelids. In deuterostomes it is conserved in cephalochordates and seems to have been lost in Ambulacraria and in Olfactores (common ancestor of urochordates and vertebrates). (Figs. 6a and 7). Altogether, TRPS is present as a single gene in metazoans with the exception of a species-specific duplication in some mollusks.

In contrast, TRPM underwent early gene duplications that led to four clades named TRPMα, β, βlike, and γ in the present study (Fig. 6a). According to our global phylogeny, these duplications would have happened in three steps in ancestral metazoans: a first gene duplication of the ancestral TRPM led to TRPMγ and ancestral TRPMα/β/βlike, a second duplication to TRPMα and ancestral TRPMβ, and a third step splitting ancestral TRPMβ into TRPMβ and TRPMβlike. The TRPMα and TRPMβ clades were previously reported and named by Himmel et al. (2020). Our present study that includes a large number of metazoan species thus adds two additional clades that we have named TRPMβlike and TRPMγ.

TRPMα is present in all metazoans from cnidarians to vertebrates. TRPMβ is also retained in all metazoans, with the exception of ecdysozoans. The additional TRPMβlike identified in this study has been conserved in bilaterians including some protostome lophotrochozoans and some deuterostomes (hemichordates and cephalochordates), but was lost in cnidarians, in ecdysozoans, and in Olfactores. The other additional TRPM, TRPMγ, also identified in the present study, was found in some cnidarians, some protostome lophotrochozans and some ecdysozoans, as well in deuterostome ambulacrarians (hemichordates and echinoderms), but was lost in the chordate ancestor (Fig. 7). It is worth noting that multiple species-specific duplications of TRPMα, TRPMβ, TRPMβlike and TRPMγ have occurred in non-vertebrate lineages, leading to the expansion of the TRPM family in some species, such as up to eight TRPM in mollusks and in hemichordates. TRPMβlike was lost before the emergence of urochordates, and TRPMγ before the emergence of chordates, so that none of them were inherited by the vertebrate lineage. All TRPM subtypes present in vertebrates have arisen from TRPMα and β (Fig. 6a).

In ancestral vertebrates, local gene-specific duplications of TRPMα would have first led to two genes located on the same chromosome, an ancestral TRPM1/3 gene and an ancestral TRPM6/7 gene (Fig. 6b). A mir-211/204 gene was inserted in an intron of the ancestral TRPM1/3. A kinase domain was added to the TRPM6/7 ancestral gene. The early vertebrate whole genome duplication 1R led to the four vertebrate α clade members: TRPM1 and 3 with their respective duplicated mir-211 and 204, and TRPM6 and 7 with their kinase domain. Despite the subsequent 2R, no additional TRPMα ohnologs are present in gnathostomes due to gene losses. Similarly, the genome hexaploidization in early cyclostomes, did not increase the number of α clade TRPM paralogs, and even led to the retention of only TRPM1 and 7. Noticeably, only mir-211 seems to have been kept in cyclostomes, and is no longer located in TRPM1 intron.

In the TRPMβ subfamily, a gene-specific duplication would have first led to an ancestral TRPM4/5 gene and an ancestral TRPM2/8 gene, where upon the ancestral TRPM4/5 gene lost the NUDT9H domain. Due to gene losses, 1R had no impact on the number of TRPMβ ohnologs, while duplication of the ancestral TRPM4/5 via genome doubling 2R in gnathostomes led to TRPM4 and TRPM5. For TRPM2/8, no additional duplicates survived the 2R genome doubling, but instead the gene underwent a local gene duplication, resulting in the TRPM2 and TRPM8 genes on the same chromosome, presumably after the second genome duplication. In cyclostomes, massive gene losses after hexaploidization led to the conservation of single ohnologs of TRPM4/5 and TRPM2/8, respectively. The NUDT9H domain was lost in gnathostome TRPM8 as well as in cyclostome TRPM2/8.

All TRPM 1 to 8 types are retained in mammals, including humans. TRPM1 and TRPM3 are retained in all gnathostomes investigated with no evidence of any lineage- or species-specific gene loss nor duplication. TRPM7 is also present in all gnathostomes, but duplicated in the amphibian lineage, and with even a further duplication in gymnophiona. TRPM2 is also conserved in all gnathostomes with species-specific duplications in some amphibians (gymnophiona), and some actinopterygians (cladistei). TRPM6 is retained in all gnathostome lineages, with possible species-specific loss in some amphibians. Thus five of the eight TRPM types in gnathostomes have been retained in all major lineages, namely TRPM1, 2, 3, 6, and 7, presumably reflecting crucial functional roles for each of these types. TRPM6 and 7 knock-outs are lethal in mouse as mentioned in our introduction. This makes the possible loss of TRPM6 in some amphibian species surprising; further studies should assess whether it has indeed been lost or has not been covered by those genome assemblies.

TRPM4 was not retrieved in birds in agreement with previous studies (Saito and Shingai 2006). Our investigation, which included species from other sauropsid lineages, showed the presence of TRPM4 in squamates, crocodilians, and chelonians, with even a possible duplication in some species (turtles), allowing us to rather precisely determine the timing of the TRPM4 loss, namely in a bird ancestor, after the separation from the other sauropsid lineages. As mentioned in the introduction, TRPM4, like TRPM5, allows influx of Na + or outflux of K+, and is involved in heart function in mammals but its knock-out is not lethal.

TRPM5 is retained in most gnathostomes but was not found in anuran amphibians in agreement with previous studies (Saito and Shingai 2006). Our investigation of representatives from other amphibian lineages retrieved TRPM5 sequences in gymnophiona species, indicating that the loss of TRPM5 was specific to the anuran lineage. TRPM5 is indispensable in mammals for the three tastes that activate G protein-coupled receptors (GPCR), namely sweet, umami, and bitter, and has this role also in chicken (Yoshida et al. 2018). Taste receptors for all three of these were found to be pseudogenized in three penguin species (Zhao et al. 2015). TRPM5 has been previously found to have reduced activity at low temperatures (Talavera et al. 2005) such as those that prevail in the natural feeding habitats of penguins whose taste buds will have a working temperature near 0 °C. Therefore, the authors of the penguin study suggested that the three GPCR tastes will be useless and hence their genes have accumulated inactivating mutations (Zhao et al. 2015). The TRPM5 gene itself has other functions than taste signal transduction in penguins, preventing it from adapting functionally to low temperatures (Zhao et al. 2015). In contrast, the TRPM5 gene of whales seems to have been pseudogenized already in the common ancestor of toothed and baleen whales 36 to 53 Mya (Feng et al. 2014). This helps explain why whales too have lost all three GPCR tastes. This raises the question of how the loss of TRPM5 can be compensated for or tolerated in whales with regard to all other functional roles of the protein in other species.

TRPM8 is retained in all sarcopterygians, and duplicated in *Xenopus tropicalis*. TRPM8 was identified as the first cold sensor in mammals (Bautista et al. 2007; Dhaka et al. 2007). Our phylogenetic analysis supports origin as a local duplicate of TRPM2/8 before the gnathostome radiation, but that it was lost in actinopterygians and chondrichthyans and thus only retained in sarcopterygians (unless the evolutionary rate in the latter group makes the phylogenetic analysis uncertain and the duplication happened early in the sarcopterygian lineage). Regardless of the time point for the origin of TRPM8, the gene lost its exons encoding the NUDT9H domain. The role of TRPM8 in cold sensation has been described not only in homeotherms (mammals and birds) but also in poikilotherms (in some amphibians) (Myers et al. 2009). The expression of TRPM8 in *Xenopus laevis* melanophores was shown to mediate both skin color and locomotor performance responses to cold temperature (Malik et al. 2023). TRPM8 is not only present in tetrapods but also in basal sarcopterygians. However, comparative studies by Lu et al. (2022, 2023), reported that TRPM8 was not cold-sensitive in dipnoi (lungfish) and that mutations of the MHR1-3 domain would have conferred cold thermosensitivity to TRPM8 in tetrapods. As TRPM8 has been considered the main sensor for cold temperature, this raises the question whether basal sarcopterygians, actinopterygians, and chondrichthyans have a different mechanism to sense cold temperatures. A recent study of Antarctic cryonotothenioid fishes concluded that TRPM4 (which is also member of the TRPMβ clade) has been under intense selection, which together with its expression in the trigeminal ganglion suggests a role in the cold adaptation of these fishes (York and Zakon 2022). Recent studies have described cold sensitivity in mouse for one of the subunits of the glutamate kainate-type ion channels, GluK2 (gene name GRIK2) (Cai et al. 2024), which was found to be expressed in dorsal root ganglion neurons. This may represent another pathway for low temperature sensor in the lineages lacking TRPM8.

Teleost specific whole genome doubling (3R) would have theoretically led to the duplication of all TRPM1 to 7 present in the actinopterygian lineage. Accordingly, two TRPM1a and b ohnologs are present in most teleost species. TRPM4a and b ohnologs were also retained in most teleost species, with some species-specific cases of additional duplication of the “a” or of the “b” 3R-ohnologs, or of loss of the “a” ohnolog. In contrast, the presence of a single 3R-ohnolog in all teleost species for TRPM3, TRPM5, and TRPM6 suggests that one ohnolog was likely lost shortly after the 3R. This is also the case for TRPM2, while some species-specific duplications have occurred for the remaining TRPM2 ohnolog. Both 3R-duplicated TRPM7 ohnologs were retained only in basal teleosts (elopomorphs and osteoglossomorphs), which thus possess a total of ten TRPM (Fig. 6). One of the TRPM7 3R-ohnolog was lost in a clupeocephalan ancestor, leading to a single gene in most teleost species. In some clupeocephalans, a second TRPM7 paralog gene arose from lineage-specific duplication of the conserved 3R ohnolog. Salmonid-specific additional whole genome doubling (4R) together with some gene-specific duplications led to up to sixteen TRPM genes as observed in *Salmo salar*.

In conclusion, our study provides a comprehensive evolutionary scenario of TRPS/TRPM genes, protein domains as well as intron-associated mir, during the metazoan radiation. It proposes a phylogenetically based classification of TRPM types across metazoans, and provides necessary tools for predicting and naming TRPM types and paralogs according to lineages and species. These data open new research avenues for functional studies of the multiple TRPM members and their potential involvement in sensing and responding to environmental changes.

## Materials and methods

### Gene searches for TRPM and TRPS sequences

BLAST searches in NCBI (https://www.ncbi.nlm.nih.gov/) or Ensembl (Ensembl release 103, https://www.ensembl.org/index.html) genome databases were performed to retrieve sequences of TRPM and of TRPS from representative species of various metazoan groups, and to search for potential paralogs including non-annotated genes. Genomes of species representing various metazoan taxa were investigated: cnidarians, protostomes, non-chordate deuterostomes, non-vertebrate chordates, and vertebrates (Table 1). Additional sequences were used such as previously identified TRPS sequences from golden apple snail, *Pomacea canaliculata,* house dust mite, *Dermatophagoides pteronyssinus,* filarial eye roundworm, *Loa loa,* tardigrade, *Ramazzottius varieornatus*, and common octopus, *Octopus vulgaris* (Himmel et al. 2020). Sequence references are provided in Table S1.

### Phylogenetic analysis of the TRPM/TRPS sequences

Phylogenetic analyses were performed to construct a global metazoan TRPM—TRPS tree and two detailed vertebrate TRPM trees. Amino acid sequences were first aligned using Clustal Omega (Sievers and Higgins 2018) with SeaView 5.0.1 software (http://doua.prabi.fr/software/seaview), and then manually adjusted (see Fasta file for the sequence alignments). The JTT (Jones, Taylor and Thornton) protein substitution matrix of the resulting alignment was determined using ProTest software (Abascal et al. 2005). The phylogenetic trees were constructed based on the sequence alignments, using the RAXML program (Randomized Axelerated Maximum Likelihood; (Stamatakis 2014) with 1,000 bootstrap replicates. Bayesian phylogenetic analyses were performed using BEAST v2.7.8 (Drummond et al. 2012) through the CIPRES Science Gateway. Markov chain Monte Carlo simulations were run for 10 million generations, sampling every 1,000 generations. The first 10% of sampled trees were discarded as burn-in. A maximum clade credibility tree was generated using TreeAnnotator and subsequently visualized using Figtree 1.4.4 (http://tree.bio.ed.ac.uk/). The alignment files can be provided by the corresponding authors upon request.

### Synteny analyses of the TRPM family in vertebrates

Synteny analyses of each TRPM genomic region (TRPM1/TRPM7, TRPM2/TRPM8, TRPM3/TRPM6, TRPM4, and TRPM5) was performed for vertebrate species at key phylogenetic positions: a cyclostome (sea lamprey), a chondrichthyan (spotted catshark), sarcopterygian tetrapods (western clawed frog, duck, and human), and actinopterygians (reedfish, spotted gar, and several teleosts). The TRPM genomic region of the reedfish was used as template. Neighboring genes were first identified using Genomicus PhyloView of Genomicus v100.01, then by Blast analysis in ENSEMBL and NCBI to search for potential paralogs of the neighboring genes, or to identify non-annotated neighboring genes. Due to the absence of the spotted catshark and European eel genomes in Ensembl and Genomicus databases, neighboring genomic regions of the TRPM were retrieved by BLAST in the respective genomes in NCBI. BLAST analyses in ENSEMBL and NCBI were performed. TRPM neighboring gene references and locations are provided in Table S2.

### Paralogon analyses of the TRPM family in vertebrates

Paralogon analysis was done with each gnathostome TRPM genomic region ie TRPM1/TRPM7, TRPM2/TRPM8, TRPM3/TRPM6, TRPM4, and TRPM5. The spotted gar TRPM genes were used as starting point and lists were compiled with genes within 10 Mb upstream and downstream of each TRPM gene. The lists were downloaded using the Biomart function in Ensembl version 80 (reedfish was not included in Biomart when this project was initiated). For TRPM4 and TRPM5, neighboring genes were also used that had previously been compiled for a syntenic gene family (Ocampo Daza and Larhammar 2018). As TRPM8 is present only in sarcopterygians, we used the human TRPM8 gene as starting point. From the gene lists, gene families with at least two members were selected. Additional members were searched with BLAST. For the neighboring gene families, phylogenetic trees in the ENSEMBL database release 103 (https://www.ensembl.org/index.html) and Panther (Thomas et al. 2022) were inspected and those that appeared to have expanded around the same time period as the basal vertebrate genome duplications, phylogenetic trees were calculated. Amino acid sequences of gene families syntenic with vertebrate TRPM genes were aligned using the MUSCLE algorithm with default settings in Jalview (Waterhouse et al. 2009). The alignments were inspected and manually curated if necessary. The curated alignment was used to create a maximum-likelihood phylogeny using IQ-TREE (Nguyen et al. 2015; Kalyaanamoorthy et al. 2017) with the following settings: -m TEST -bb 1000 -alrt 1000 -wbtl -wsr. Node support was calculated using the SH-aLRT branch test and non-parametric UltraFast Bootstrap method (Hoang et al. 2018). For each family, one or more outgroups were included to root the tree. The resulting trees were displayed in ITOL (Interactive tree of life) (Letunic and Bork 2021, PMID 33885785) available at https://itol.embl.de. References and locations of TRPM paralogon neighboring genes families are provided in Table S3.

### Micro RNA search and phylogeny analysis

Sequences for human microRNA 204 (hsa-mir-204, accession number MI0000284, 110 nucleotides) and 211 (hsa-mir-211, accession number MI0000287, 110 nucleotides) were retrieved from miRbase (https://mirbase.org), Kozomara et al. (2019) and used for blast in NCBI and Ensembl. BLAST searches were performed in Ensembl database on genomes from ecdysozoans (*Drosophila melanogaster* and *C. elegans*), urochordates (*Ciona intestinalis* and *savignyi*), cyclostomes (hagfish, *Eptatretus burgeri* and lamprey, *Petromyzon marinus*), chondrichthyan (elephant shark), sarcopterygians (coelacanth, human, opossum, platypus, wombat, mice), non-teleost actinopterygians (reedfish and spotted gar), and various teleosts (Asian bonytongue, Atlantic herring, zebrafish, Mexican tetra, Atlantic salmon, Atlantic cod, Japanese medaka, seabream, Nile tilapia, stickleback, turbot, fugu). BLAST searches were performed in the NCBI database in genomes from cephalochordates (*Branchiostoma belcheri* and *floridae*), cyclostomes (hagfish *Myxine glutinosa* and lamprey *Lethenteron reissneri*), and tetrapods (chicken and *Xenopus tropicalis*). In most cases the mir sequences obtained from the BLAST search were too short, so the corresponding genomes were further explored to extend the sequences. To assess the lack of mir sequence in TRPM1 intron in cyclostomes, we performed local BLAST with BioEdit software version 7.7.1 (Hall 1999) and BLAST + software version 2.17.0 from NCBI. Mir-204 and mir-211 nucleotide sequences were aligned using Clustal Omega with SeaView 5.0.1 software and the phylogeny tree was constructed using the RAXML program with 1,000 bootstrap replicates, and visualized using Figtree 1.4.4.

## Supplementary Material

msag098_Supplementary_Data

## Data Availability

The sequence alignments underlying the phylogenetic analyses in this article will be shared on request to the corresponding authors.
