# Supplementary material for: Evolution in metazoans of the TRPM channel family involves multiple gains and losses of genes and domains
Source: Mol Biol Evol. 2026 Apr 11;43(5):msag098. doi: 10.1093/molbev/msag098 (PMC13143021; doi:10.1093/molbev/msag098)

Supplementary Figure S1A. Global phylogeny of metazoan protein sequences of TRPM and TRPS inferred using the ML method. The phylogenetic tree displays all species included in Figure 2 with their name and current gene references in databases. See Supplementary Table S1 for detailed information

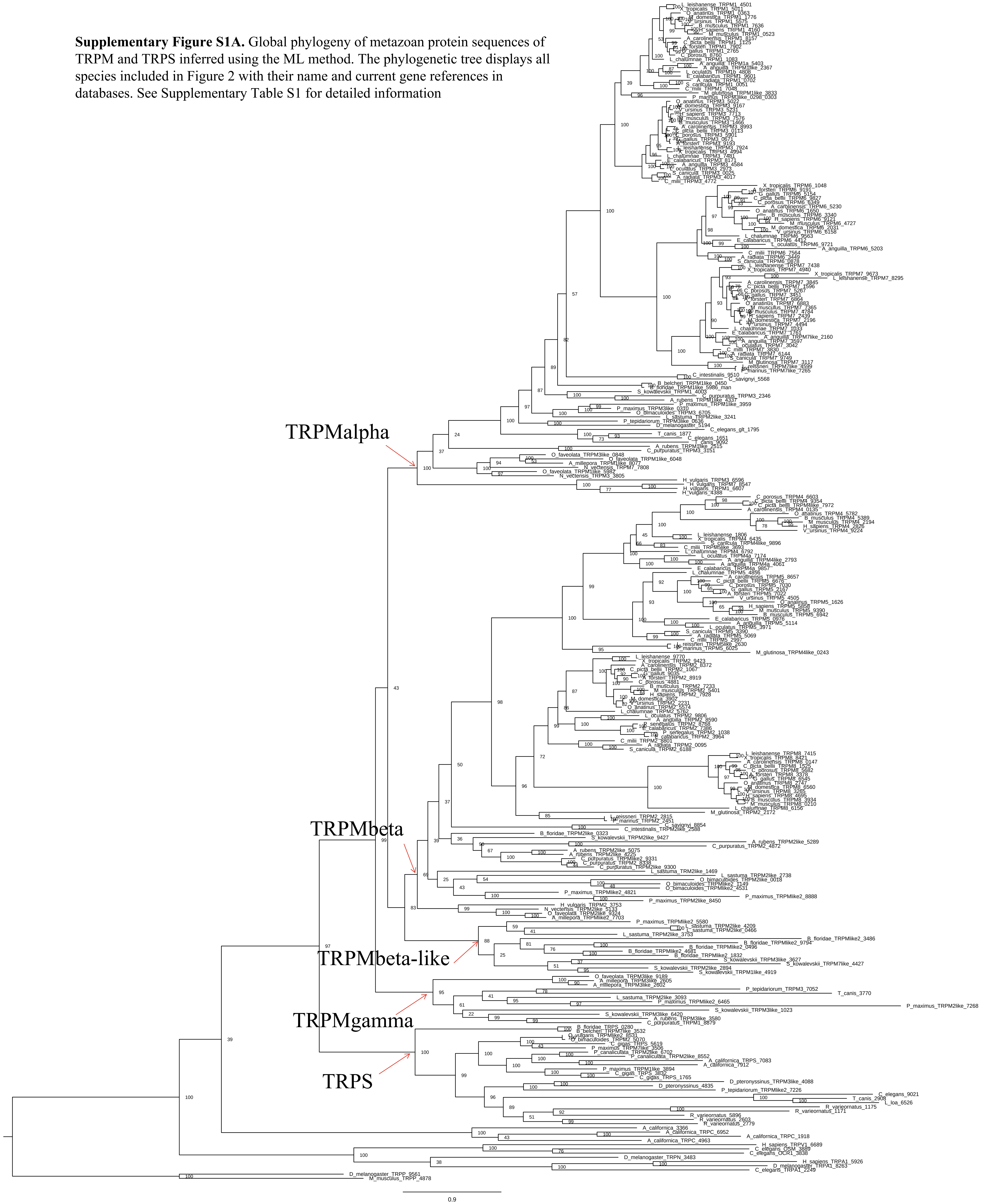

Supplementary Figure S1B. Global phylogeny of metazoan TRPM and TRPS protein sequences inferred using the Bayesian method. The phylogenetic tree displays all species included in Figure 2 with their name and current gene references in databases. See Supplementary Table S1 for detailed information.

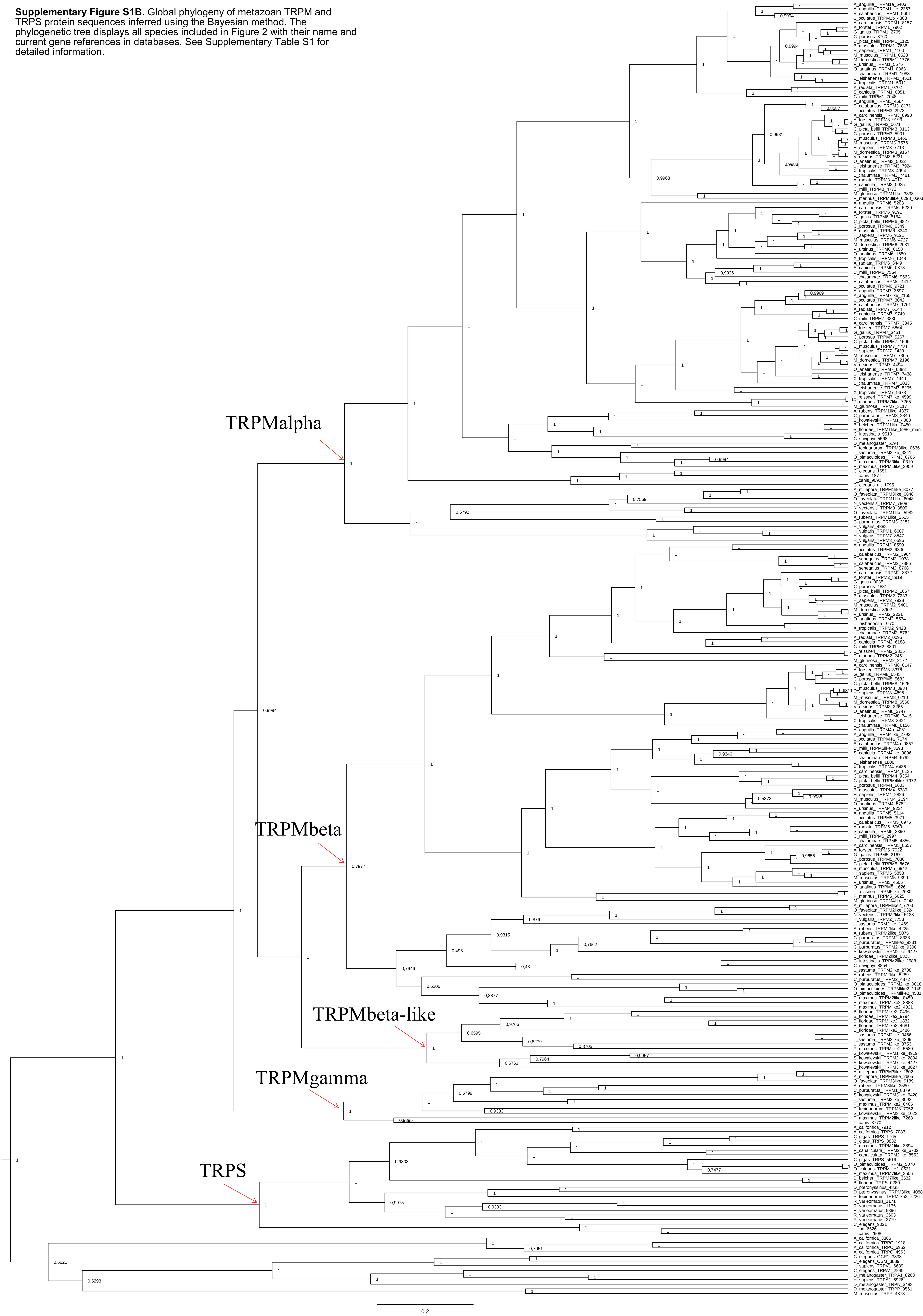

Supplementary Figure S2A. Detailed evolutionary scenario of TRPM and TRPS in early metazoans and cnidarians

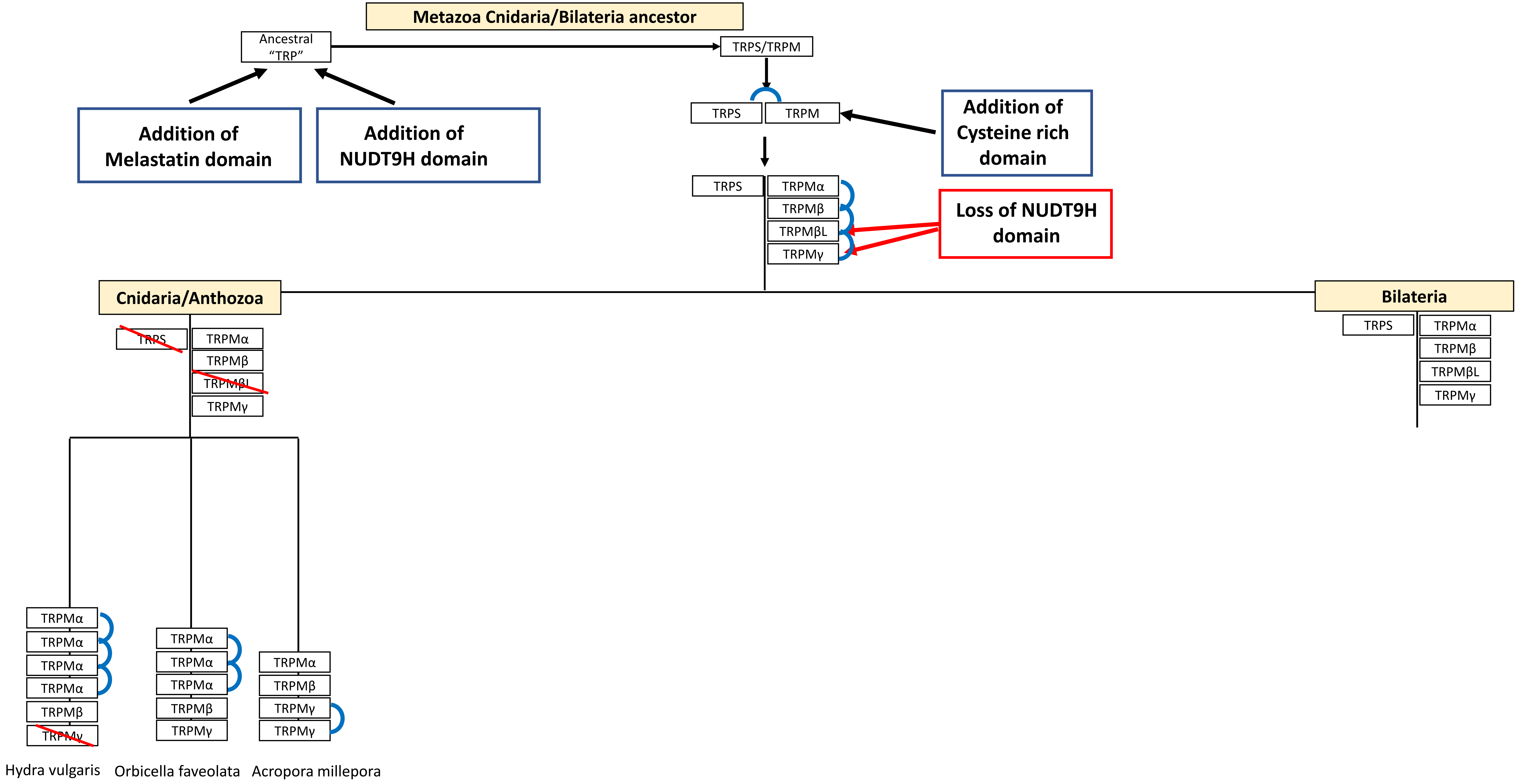

Supplementary Figure S2B. Detailed evolutionary scenario of TRPM and TRPS in bilaterian protostomes

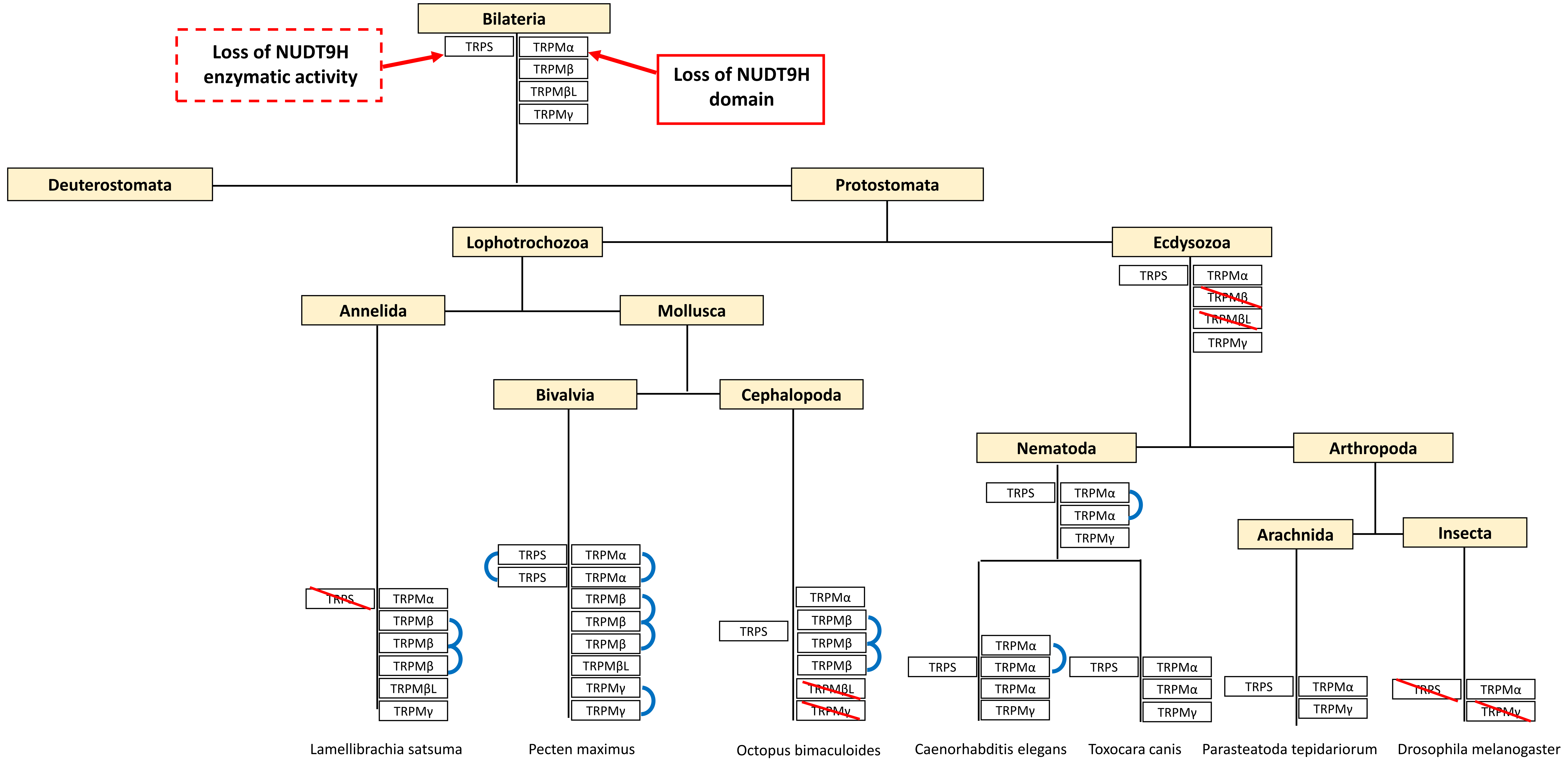

Supplementary Figure S2C. Detailed evolutionary scenario of TRPM and TRPS in non-vertebrate bilaterian deuterostomes

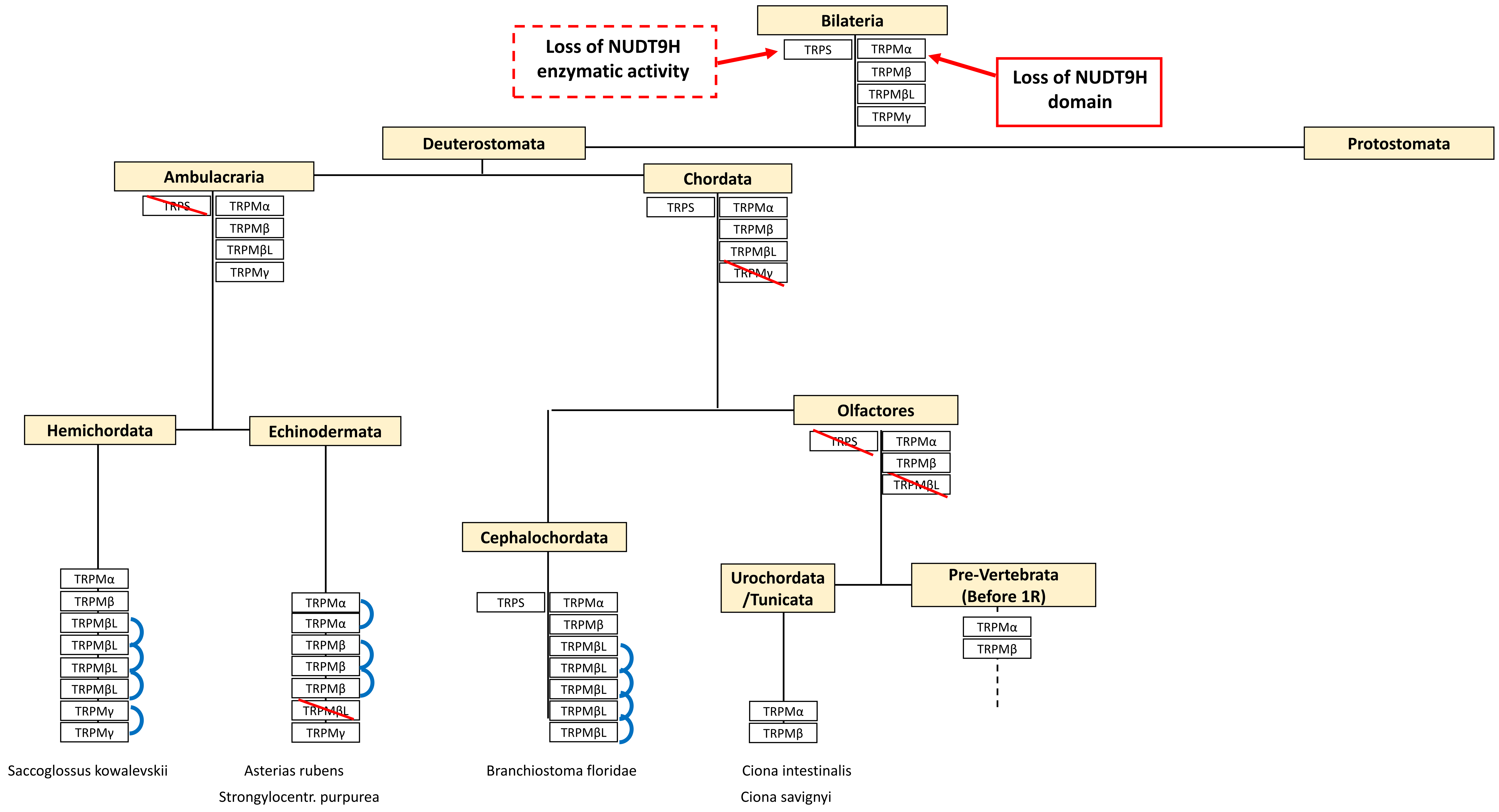

Supplementary Figure S2D. Detailed evolutionary scenario of TRPM in cyclostomes and chondrichthyans

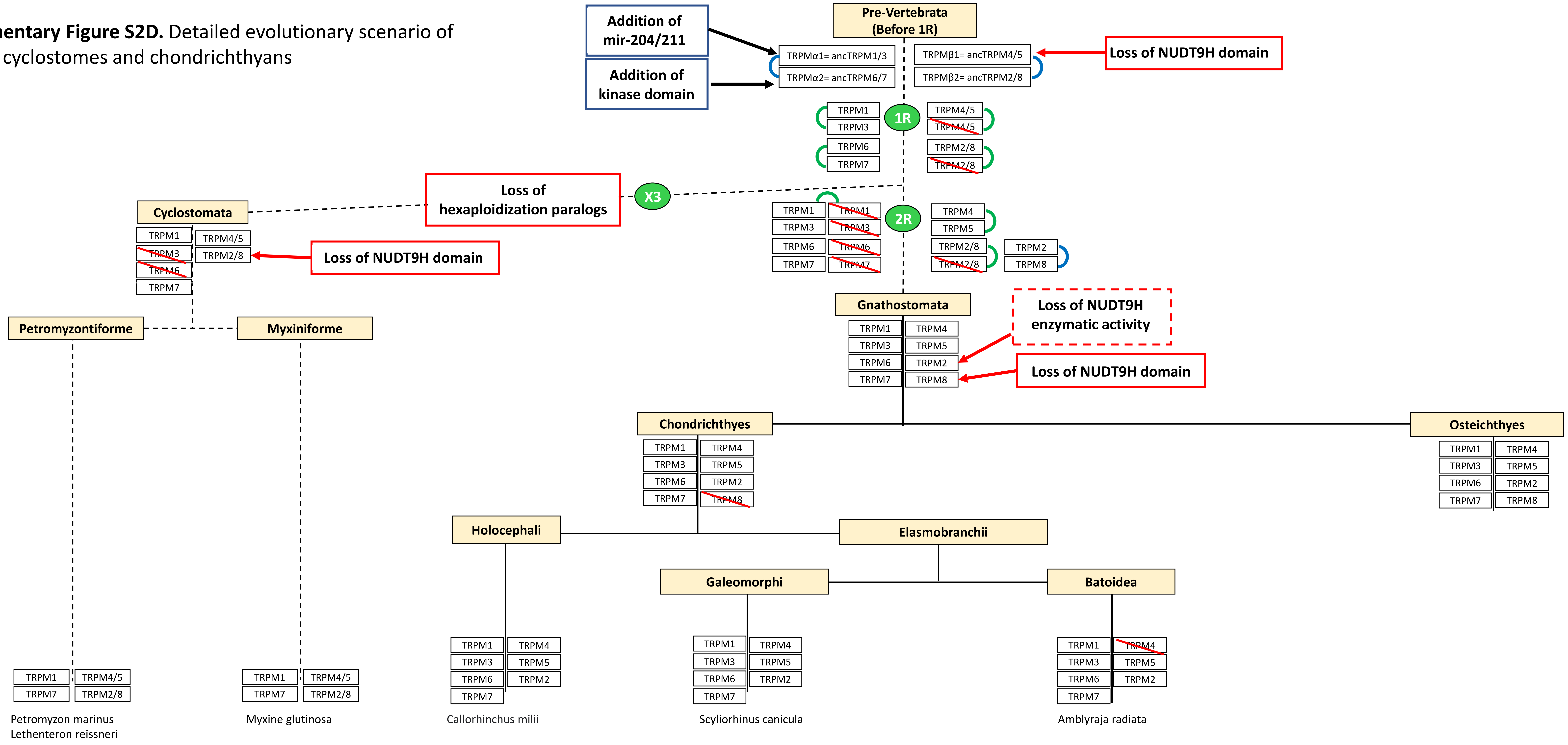

Supplementary Figure S2E. Detailed evolutionary scenario of TRPM in sarcopterygians

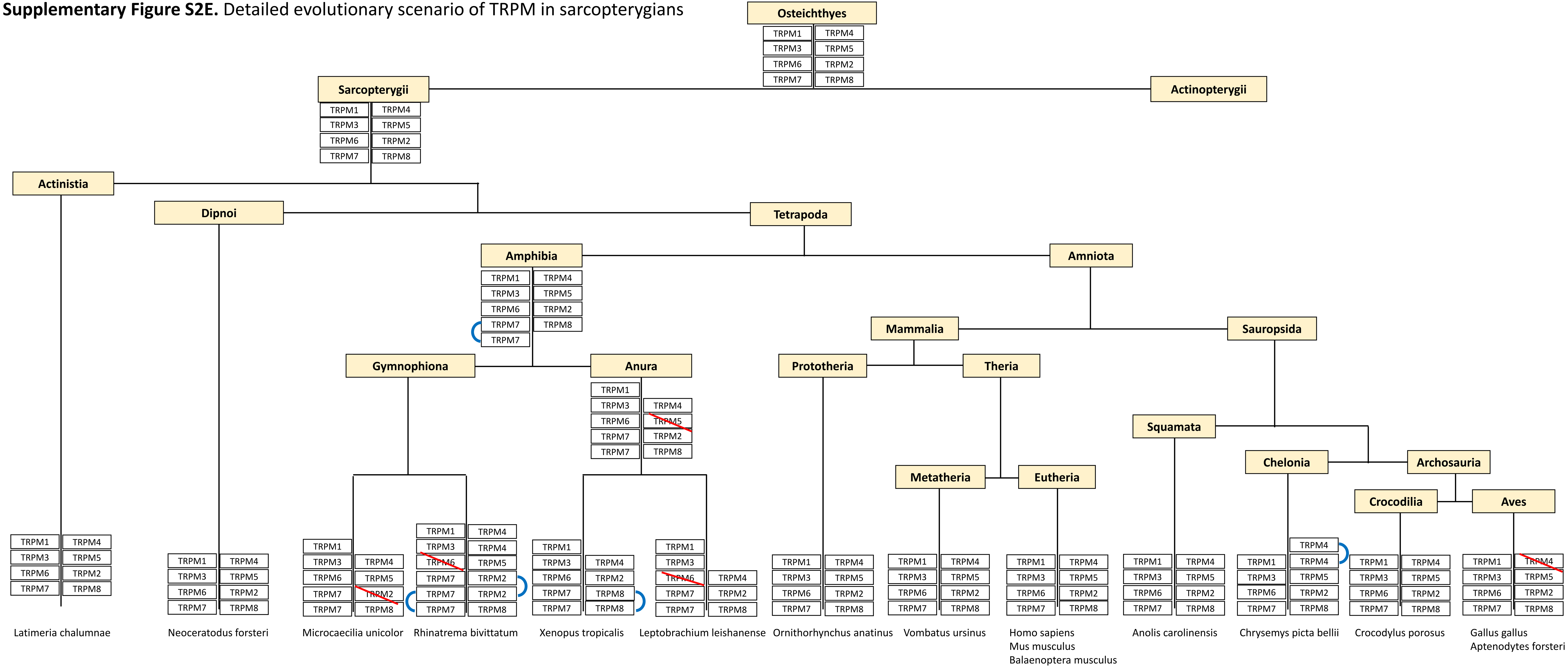

Supplementary Figure S2F. Detailed evolutionary scenario in actinopterygians (non-teleosts and basal teleosts)

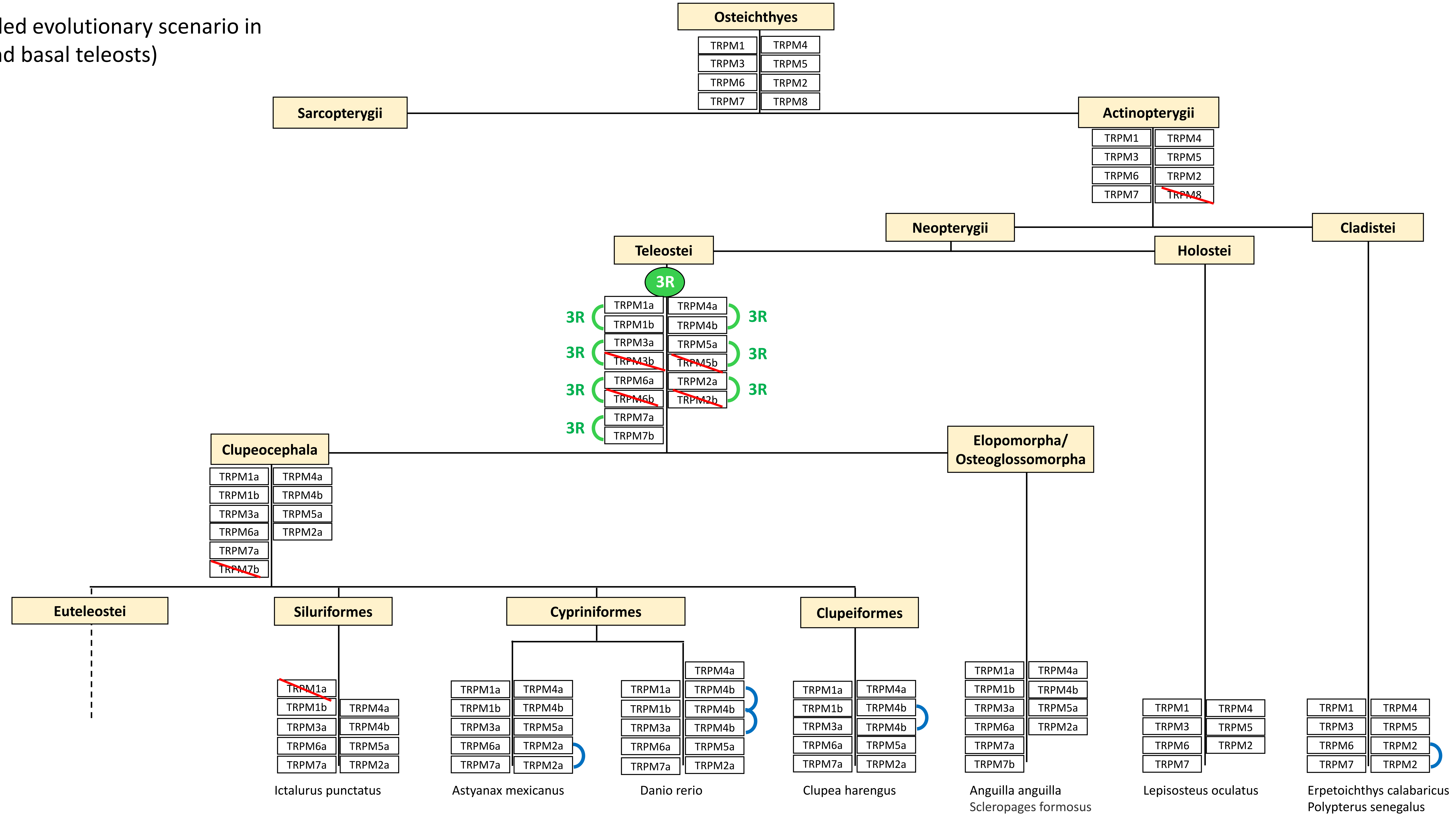

Supplementary Figure S2G. Detailed evolutionary scenario in actinopterygian euteleosts

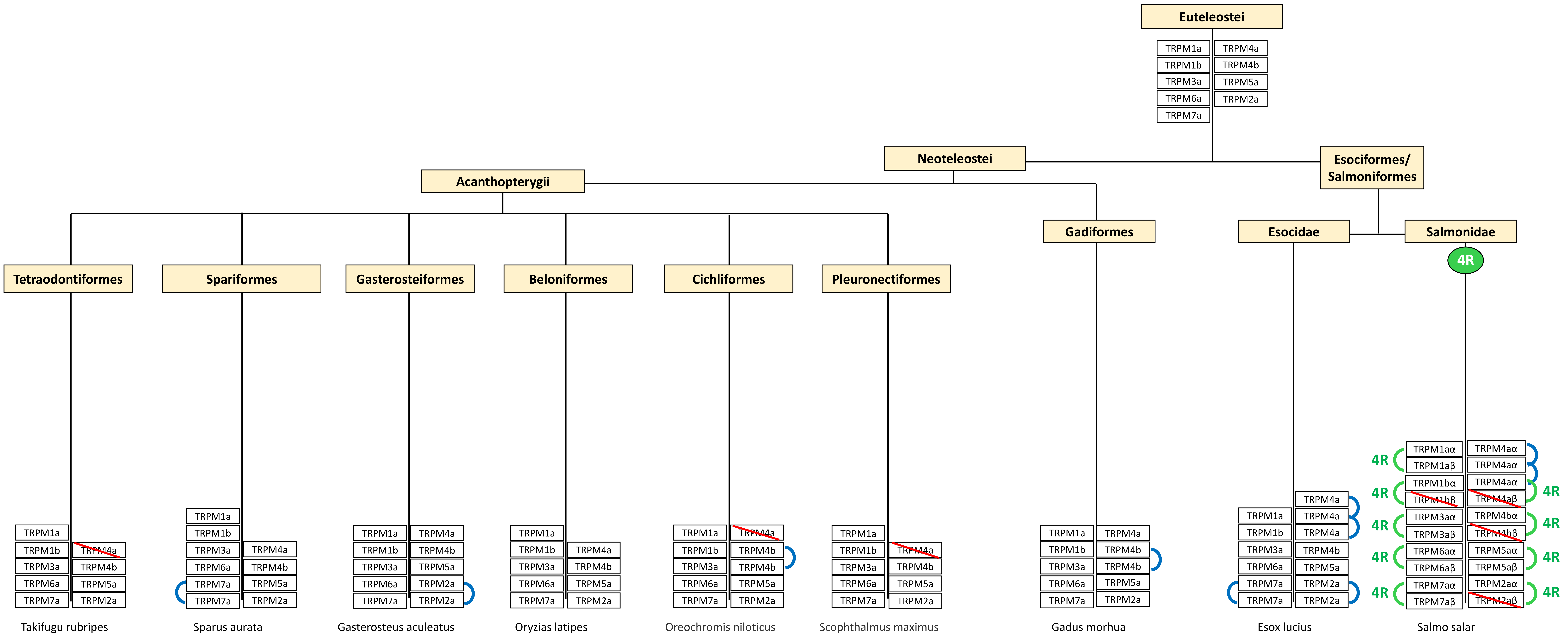

Figure S3A. Phylogenetic relationships of vertebrate TRPM1,3,6,7 sequences (TRPMα). Tree topology inferred with the phylogenetic maximum likelihood method from an alignment of 178 amino acid sequences from vertebrates (cyclostomes, chondrichthyans, actinopterygians and sarcopterygians), with the reedfish *Erpetoichthys calabaricus* TRPM2 sequence used to root the tree. Boostrap values over 1000 replicates (%) are indicated. This phylogenetic analysis clusters vertebrate TRPM1,3,6,7 sequences into two main clades (TRPM1,3 and TRPM6,7). Following the teleost-specific whole genome duplication (3R), duplicated TRPM1a and b ohnologs are found in many species, duplicated TRPM7a and b ohnologs in early-diverging teleost lineages, and only a single paralog of TRPM3 and TRPM6 in all species investigated. See Supplementary Table S1 for sequence accession numbers.

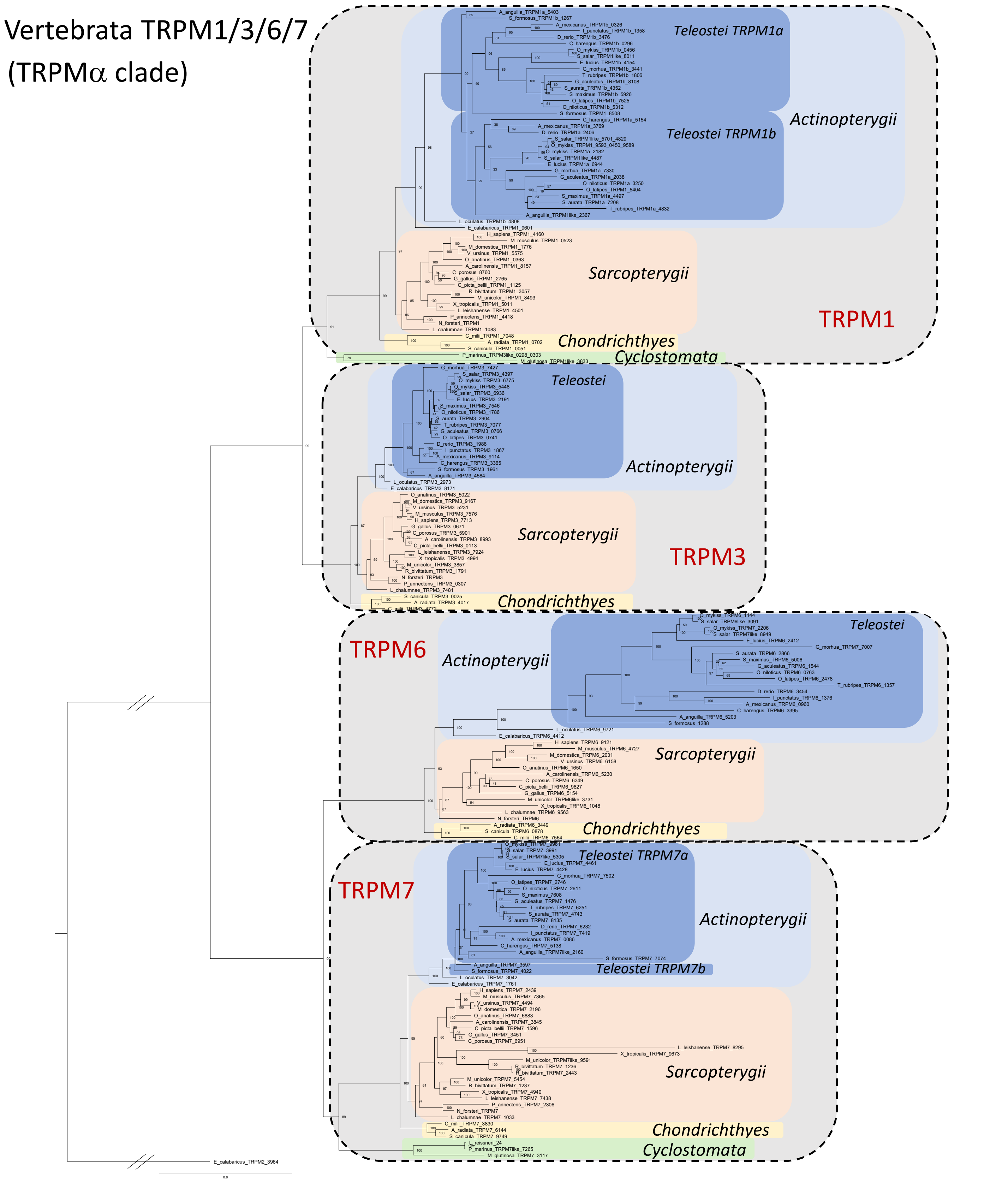

Figure S3B. Phylogenetic relationships of vertebrate TRPM2,4,5,8 sequences (TRPMβ).
Tree topology inferred with the phylogenetic maximum likelihood method from an alignment of 159 amino acid sequences of vertebrates (cyclostomes, chondrichthyans, actinopterygians and sarcopterygians), with the reedfish TRPM1 sequence used to root the tree. Boostrap values over 1000 replicates (%) are indicated. See Supplementary Table S1 for sequences accession numbers. This phylogenetic analysis clusters vertebrate TRPM2,4,5,8 (TRPMβ) sequences into two main clades (TRPM2,8 and TRPM4,5). TRPM8 was lost independently in chondrichthyans and actinopterygians, and conserved only in sarcopterygians. Following the teleost-specific whole genome duplication (3R), duplicated TRPM4a and b ohnologs are found in many species, while a single paralog of TRPM2 and TRPM5 is present.

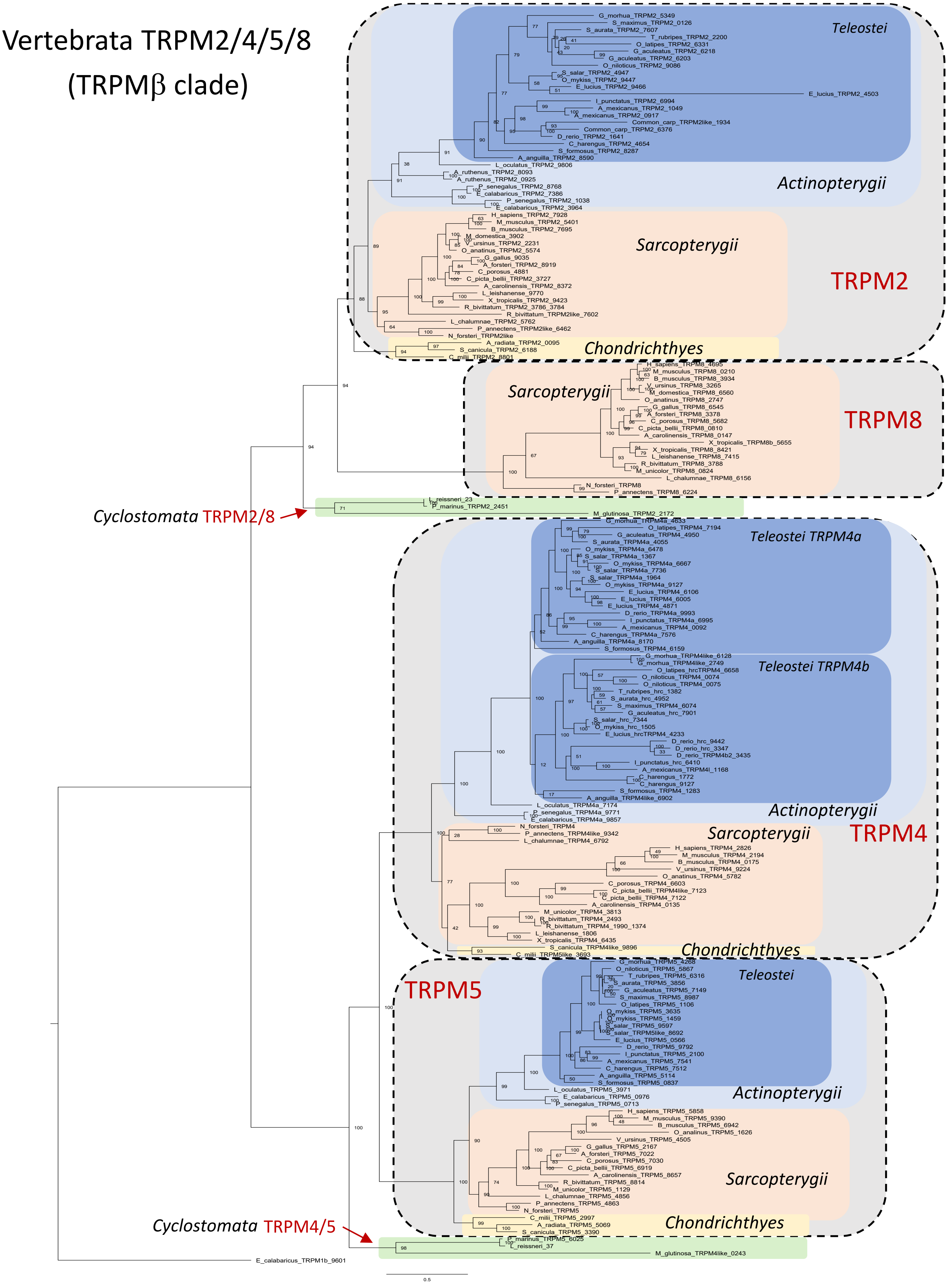

Supplementary Figure S4A. Conserved synteny between vertebrate TRPM1,7 genomic regions**. R**eedfish TRPM1,7 genomic region is used as template (black frame). Eight neighboring genes are displayed. Red frames highlight orthologous TRPM genes across vertebrate species. Blue arrows indicate TRPM-specific local gene duplications and black crosses genes that are missing. See Supplementary Table S2 for TRPM and neighboring gene sequence accession numbers.

TRPM1/TRPM7

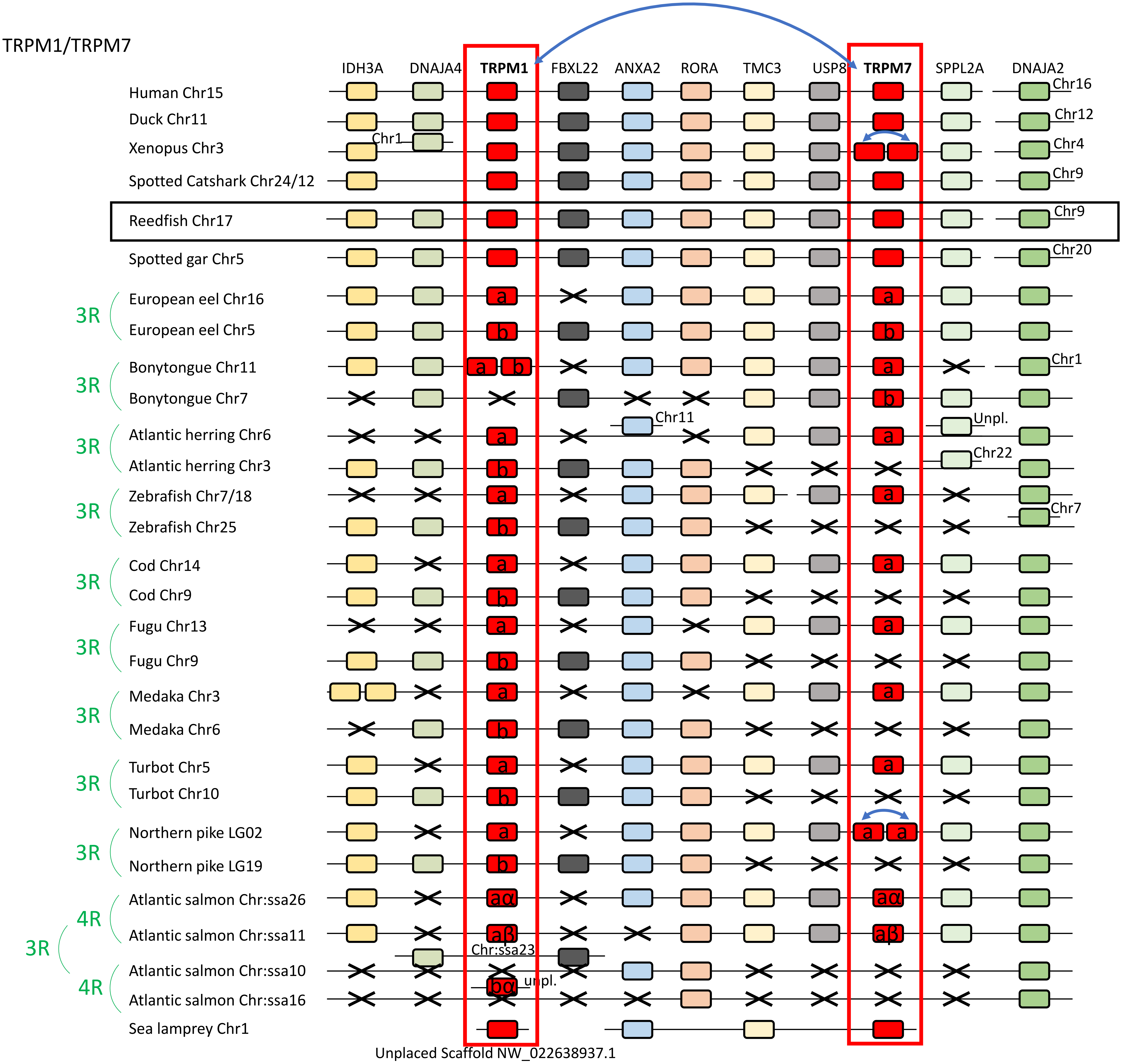

Supplementary Figure S4B. Conserved synteny between vertebrate TRPM3,6 genomic regions. Reedfish TRPM3,6 genomic region is used as template (black frame). Seven neighbouring genes are displayed. Red frames highlight orthologous TRPM genes across vertebrate species. Blue arrows indicate local gene duplications and black crosses genes that are missing. See Supplementary Table S2 for TRPM and neighboring gene sequence accession numbers.

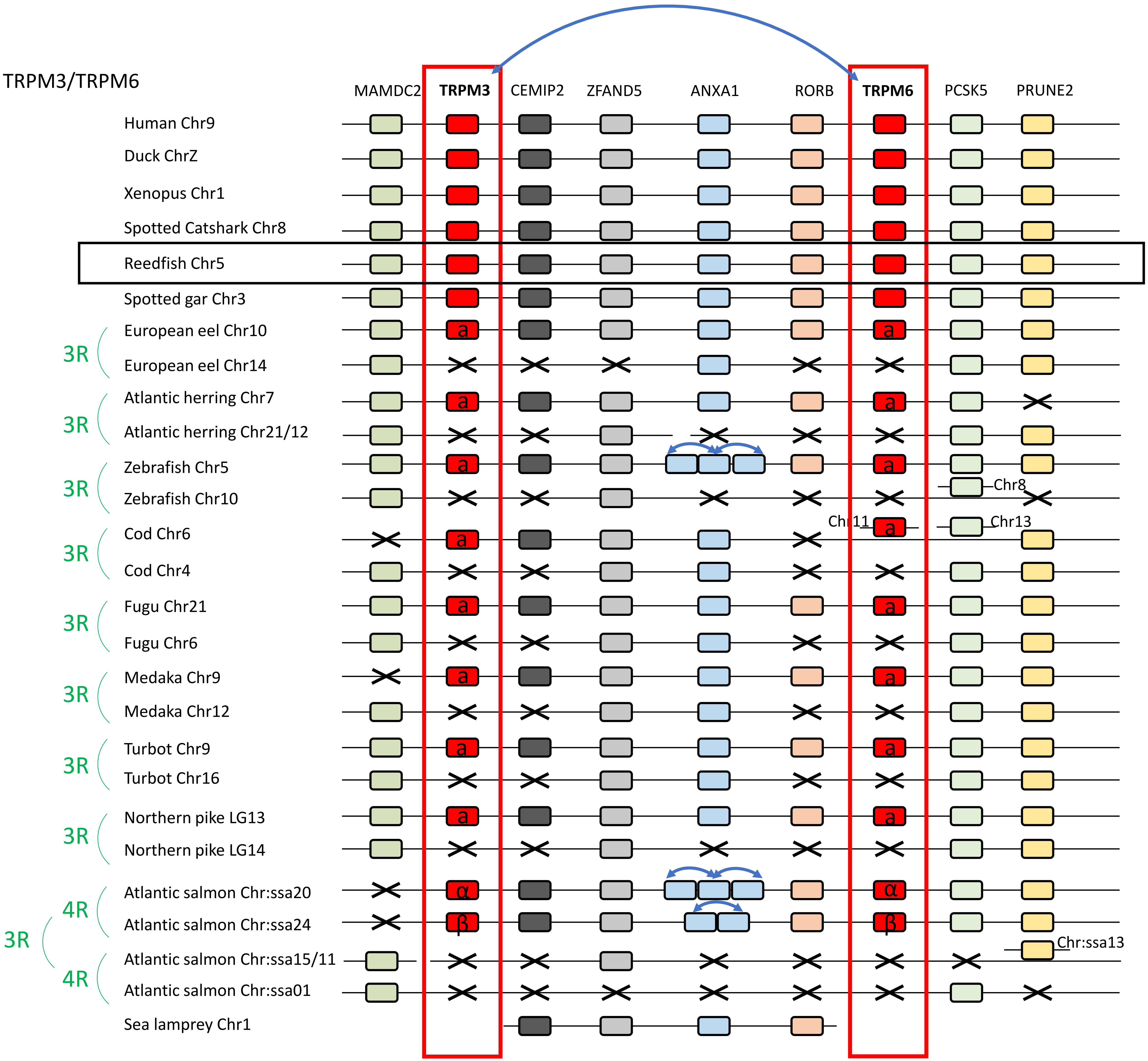

Supplementary Figure S4C. Conserved synteny between vertebrate TRPM2/8 genomic regions. Reedfish TRPM2 genomic region is used as template (black frame). Nine neighbouring genes are shown. Red frames highlight orthologous TRPM genes across vertebrate species. Blue arrows indicate local gene duplications and black crosses genes that are missing. See Supplementary Table S2 for TRPM and neighboring gene sequence accession numbers.

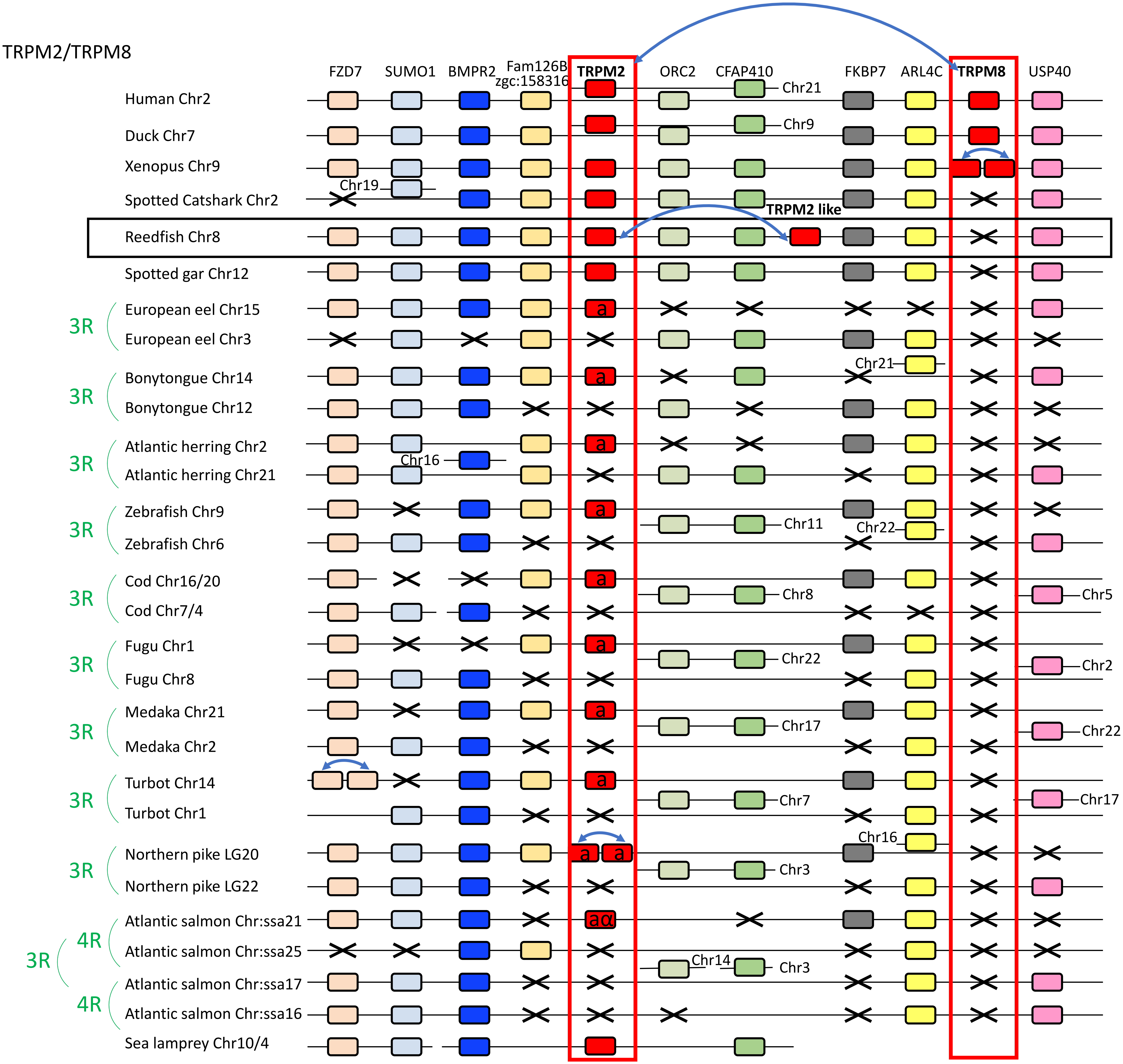

Supplementary Figure S4D. Conserved synteny between vertebrate TRPM4 genomic regions. Reedfish TRPM4 genomic region is used as template (black frame). Eight neighbouring genes are shown. Red frames highlight orthologous TRPM genes across vertebrates. Blue arrows indicate local gene duplications and black crosses genes that are missing. See Supplementary Table S2 for TRPM and neighboring gene sequence accession numbers.

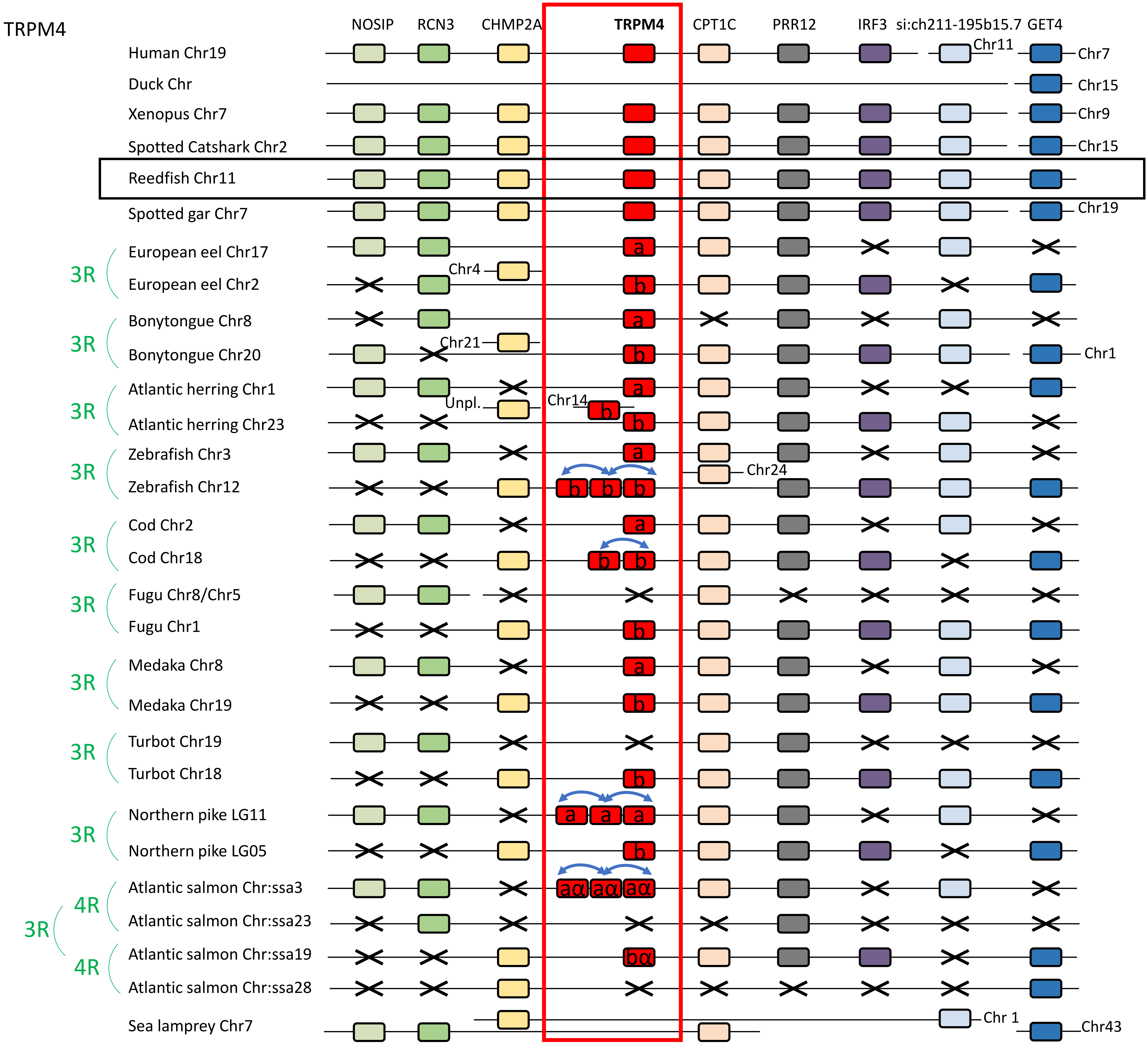

Supplementary Figure S4E. Conserved synteny between vertebrate TRPM5 genomic regions. Reedfish TRPM5 genomic region is used as template (black frame). Seven neighbouring genes are shown. Red frames highlight orthologous TRPM genes across vertebrate species. Black crosses indicate genes that are missing. See Supplementary Table S2 for TRPM and neighboring gene sequence accession numbers.

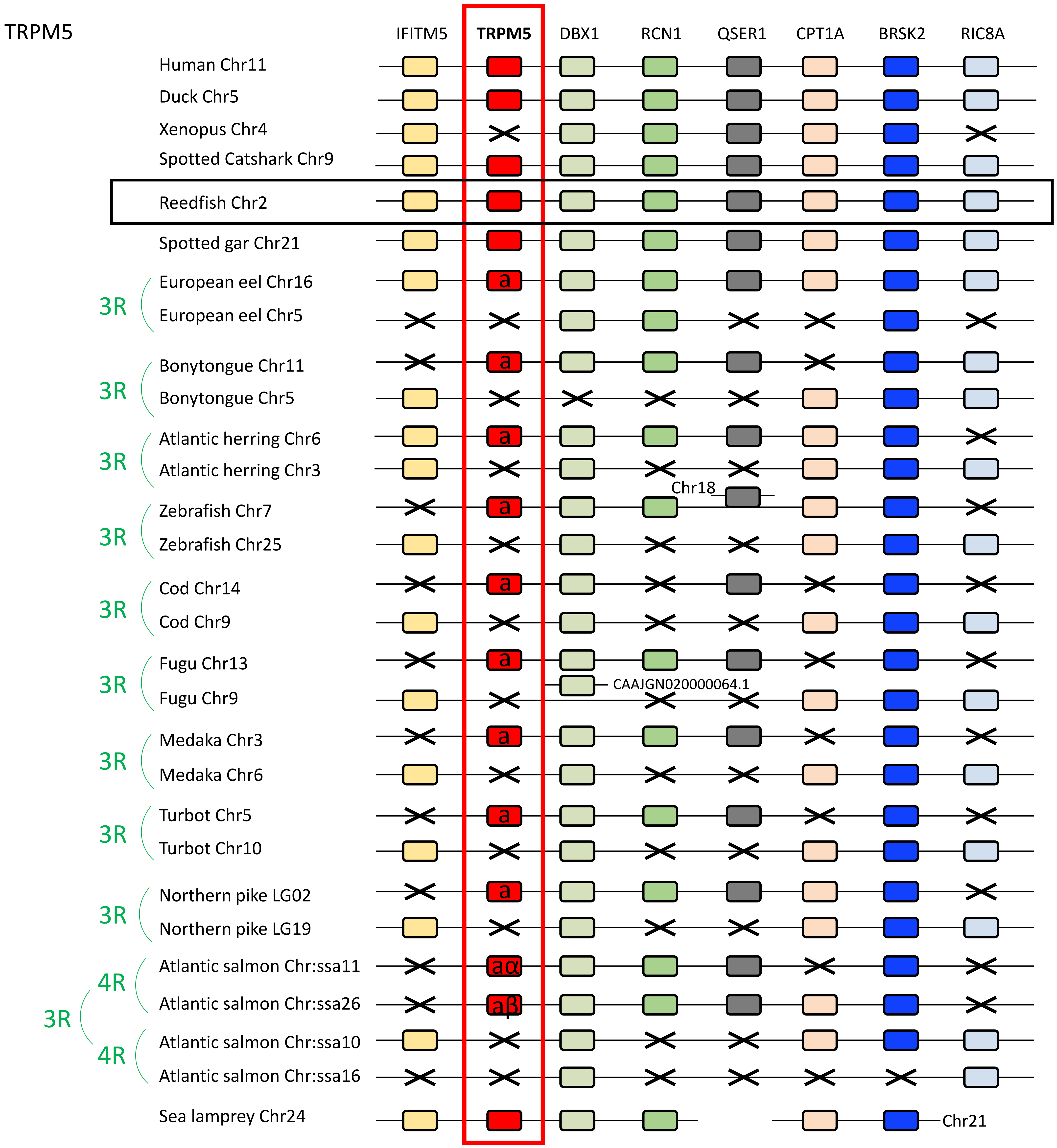

Supplementary Figure S5A

Phylogenetic trees of neighbouring gene families in paralogon with TRPM1,3,6,7 (see Fig. 3).

ANXA	Annexin A
CSNK1G	Casein kinase 1 gamma
FBN	Fibrillin
LMN	Lamin
MEGF/PEAR	Multiple EGF-like domains/Platelet endothelial aggregation receptor 1
PIP5K1	Phosphatidylinositol-4-phosphate 5-kinase type 1
ROR	RAR-related orphan receptor
SEMA6	Semaphorin 6
TJP	Tight junction protein ZO-1
TNFAIP8	Tumor necrosis factor alpha-induced protein 8

Supplementary Figure S5A (1/2). Phylogenetic analysis of neighbouring genes of vertebrate TRPM1, 3, 6, 7 paralogon.

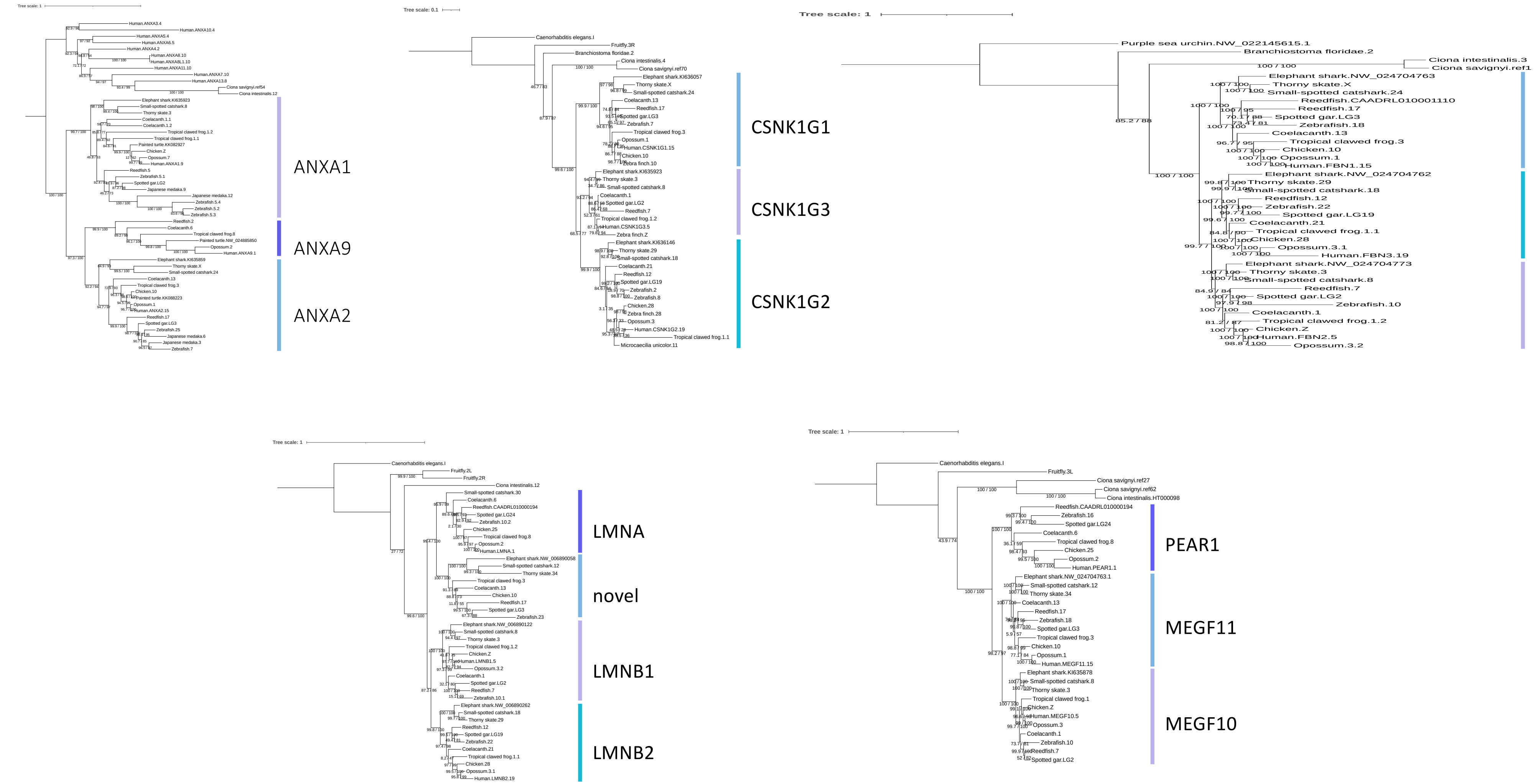

Supplementary Figure S5A (2/2). Phylogenetic analysis of neighbouring genes of vertebrate TRPM1, 3, 6, 7 paralogon.

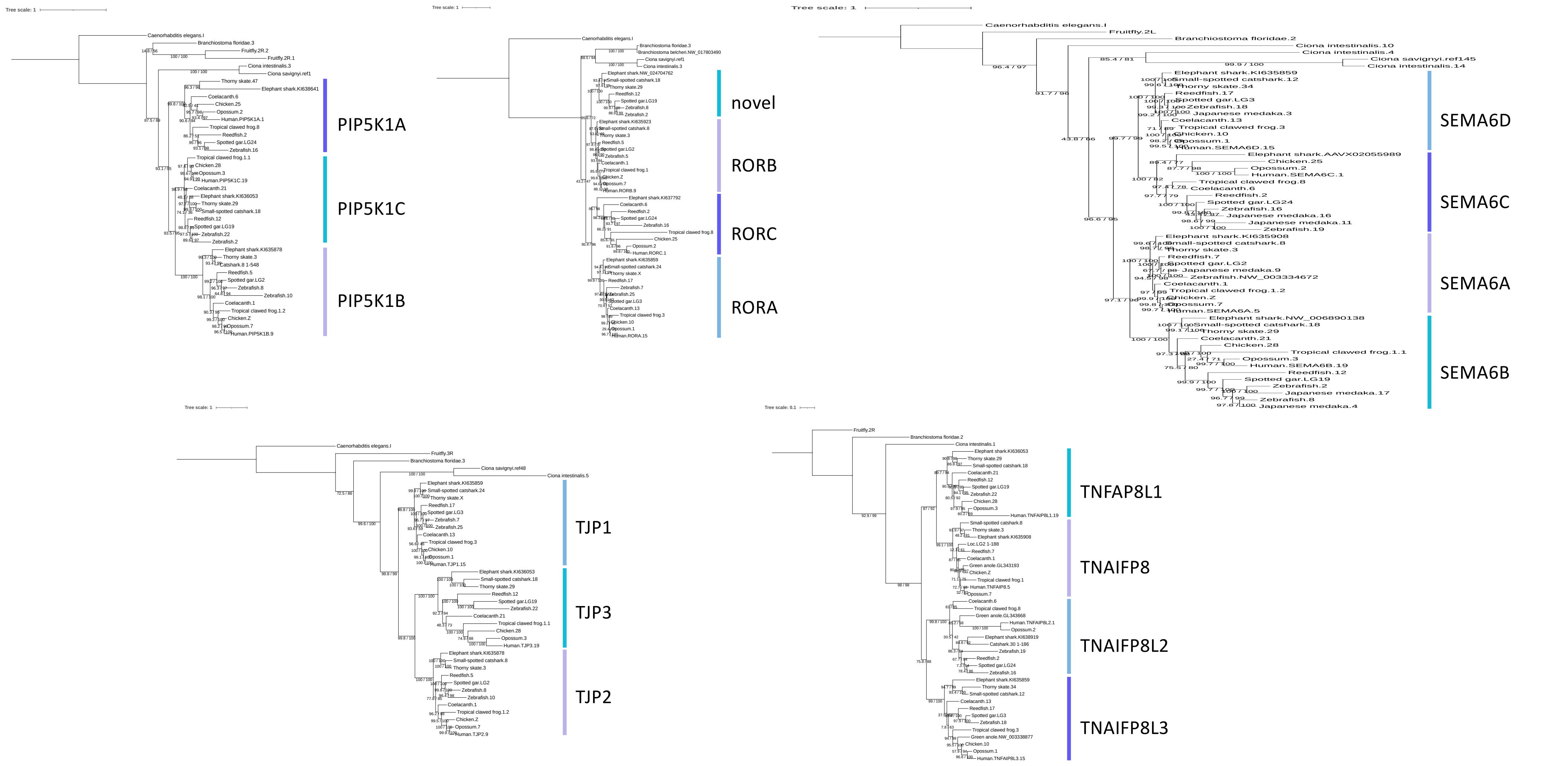

Supplementary Figure S5B

Phylogenetic trees of neighbouring gene families in paralogon with TRPM2,8 (see Fig. 4)

ARL4	ADP-ribosylation factor-like protein 4
COL6A	Collagen type VI alpha chain
DIP2	Disco-interacting protein 2
HDAC	Histone deacetylase
HYCC	Hyccin – Hypomyelination and congential cataract
IGF2BP	Insulin-like growth factor 2 mRNA-binding protein
LRRFIP	LRR-binding FLII-interacting protein
MARCHF	Membrane associated ring-CH-type finger
PFK	Phosphofructokinase
SH3BP4	SH3 domain binding protein 4

Supplementary Figure S5B (1/2). Phylogenetic analysis of neighbouring genes of vertebrate TRPM2,8 paralogon.

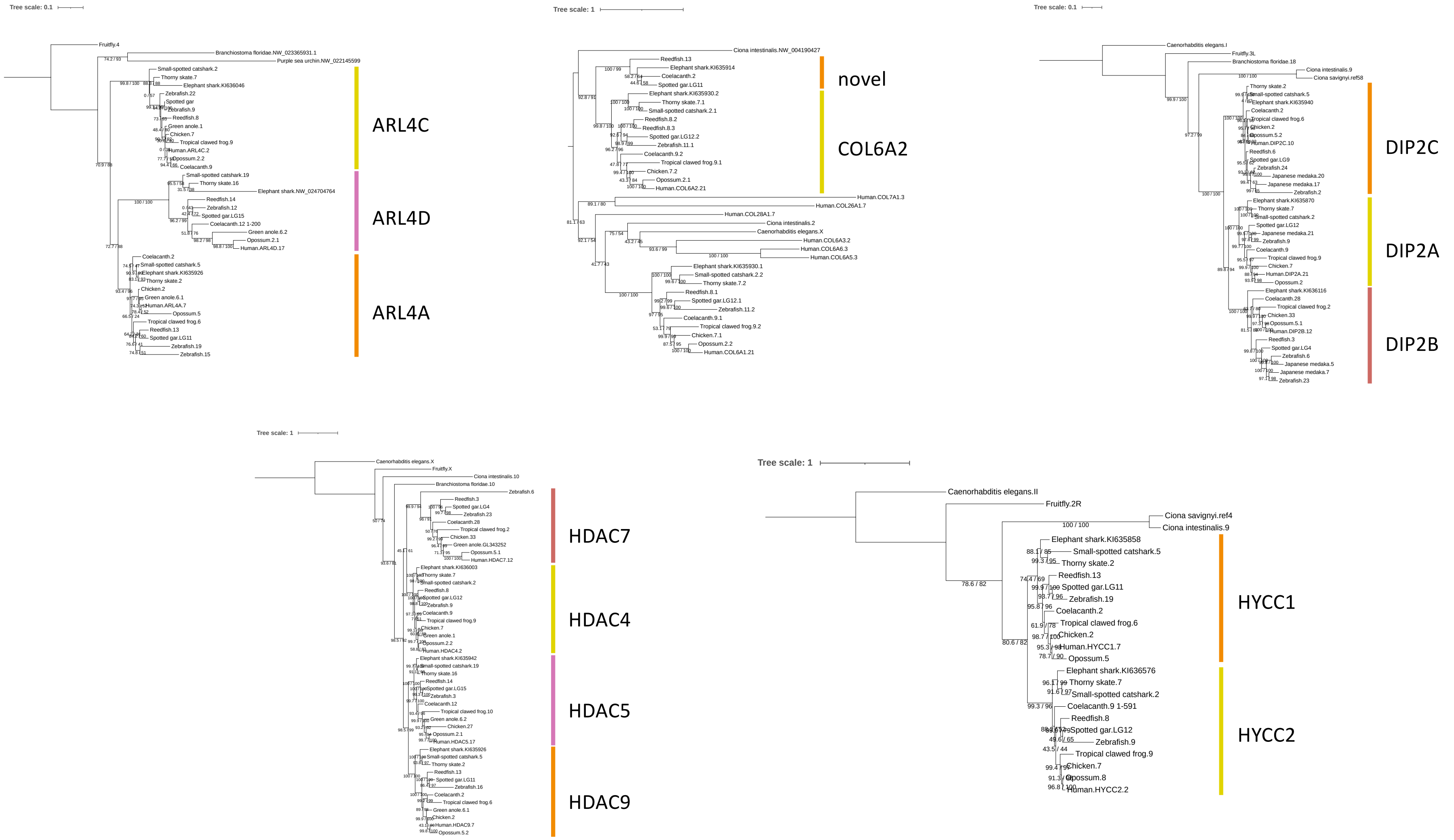

Supplementary Figure S5B (2/2). Phylogenetic analysis of neighbouring genes of vertebrate TRPM2,8 paralogon.

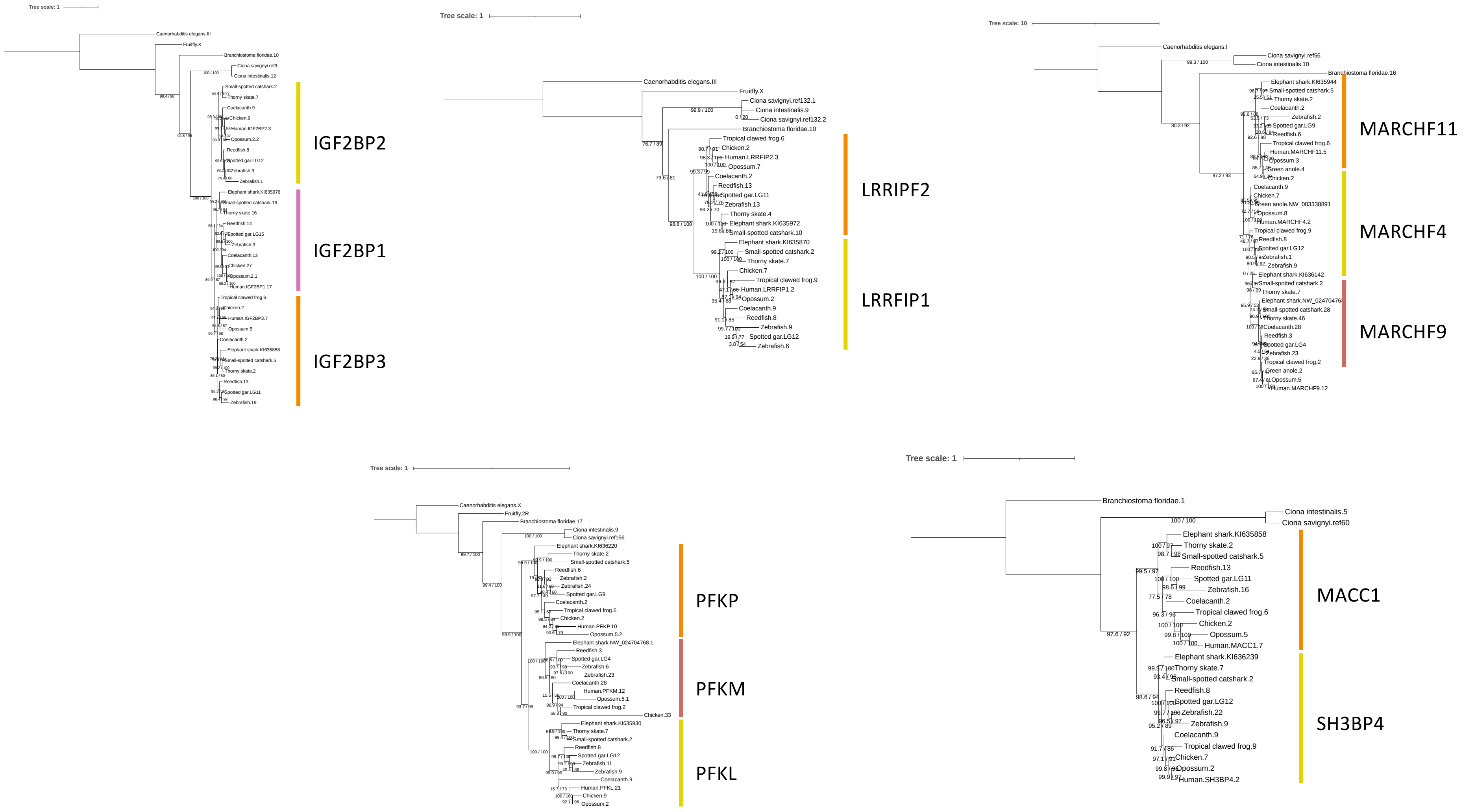

Supplementary Figure S5C

Phylogenetic trees of neighbouring gene families in paralogon with TRPM4,5 (see Fig. 5)

KCNJ8	Potassium channel inward rectifyer
MYBP	Myosin-binding protein
PPFIA	PTPRF interacting protein alpha
RIC8	Resistance to inhibitors of cholinesterase 8
SYT1	Synaptotagmin 1
SYT3	Synaptotagmin 3

Supplementary Figure S5C. Phylogenetic analysis of neighbouring genes of vertebrate TRPM4,5 paralogon.

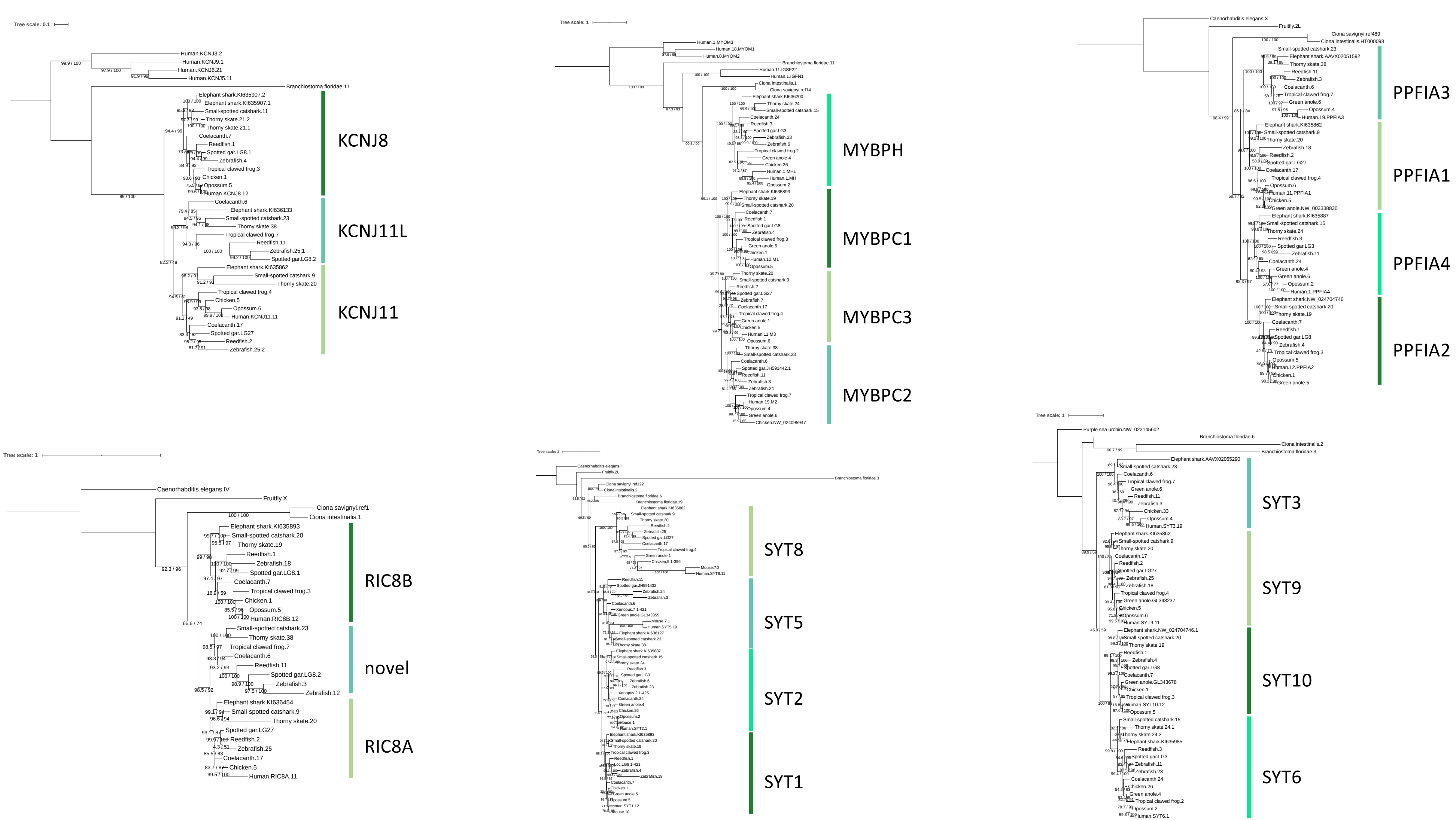

Supplementary Figure S6A. Alignment of NUDT9H sequences of TRPS and TRPM from various metazoans.

The sequence comparison highlights the conservation of key amino-acids (*) for catalytic ADPRase activity according to Iordanov et al, 2019 in non-vertebrate TRPMβ (white boxes) and their mutation leading to the loss of enzymatic activity in vertebrate TRPM2, as well as in non-vertebrate TRPS

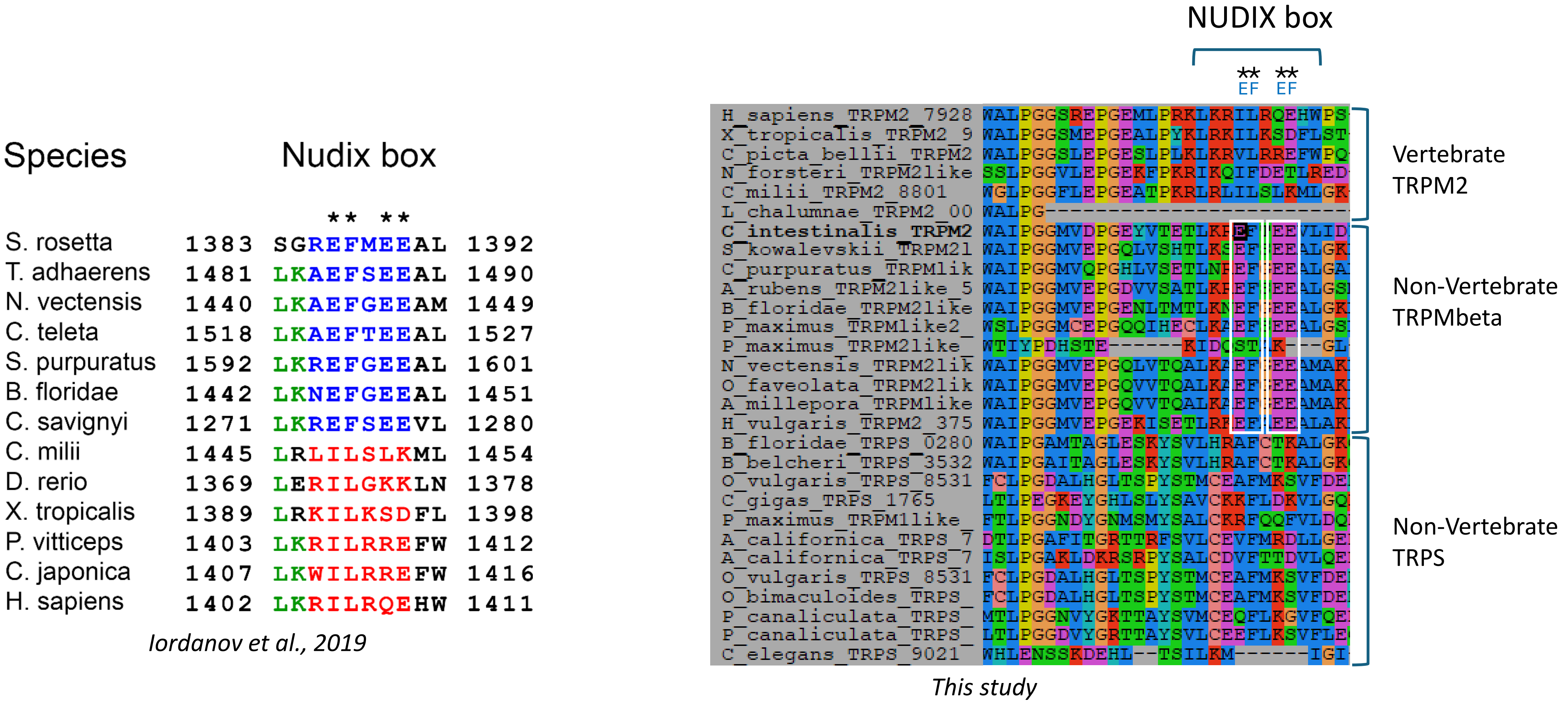

Supplementary Figure S6B. Alignment of pore filter sequences of TRPS and TRPM from various metazoans.

The sequence comparison highlights the conservation of key amino-acids for pore stability according to Iordanov et al, 2019) in non-vertebrate TRPMβ (white box) and their mutation leading to intrinsic activation in gnathostome TRPM2, cyclostome TRPM2/8, as well as in non-vertebrate TRPS

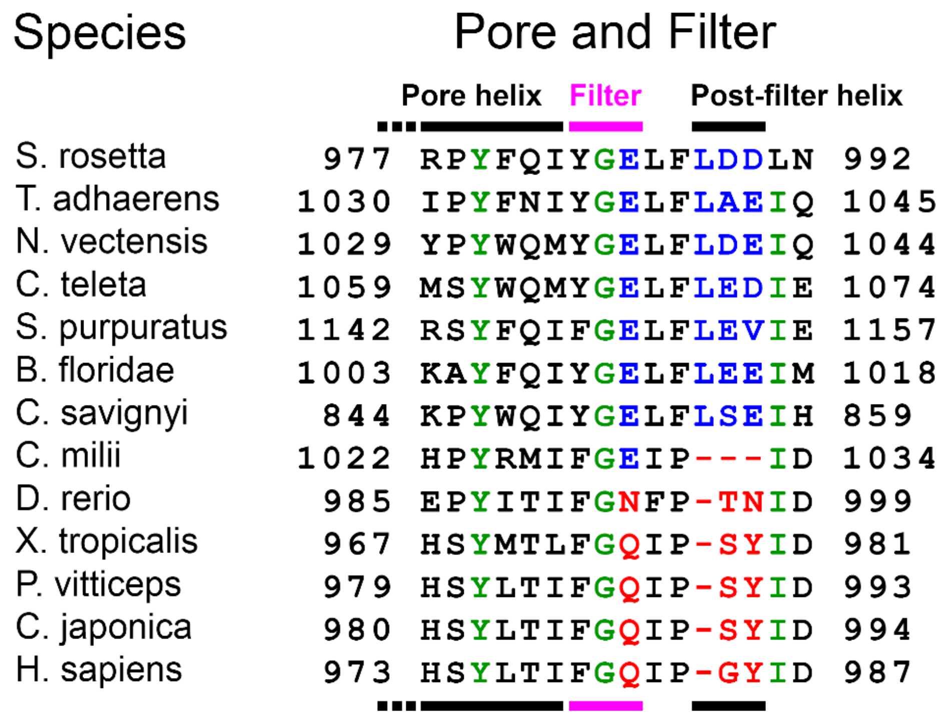

Iordanov et al., 2019

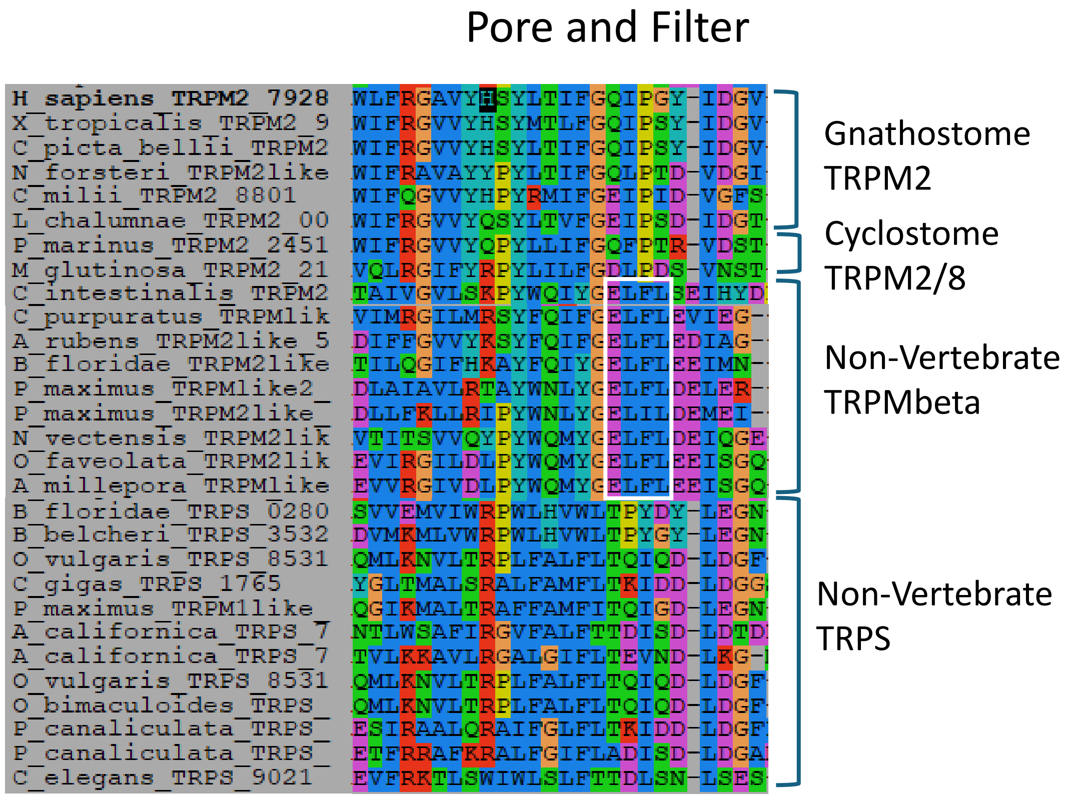

This study

Supplementary Figure S7. Phylogenetic tree of human and cnidarian alpha kinases, human and cnidarian elongation factor 2, kinase domain of vertebrate TRPM6 and TRPM7, and cnidarian kinases wrongly annotated TRPM6 or TRPM6-like in databases

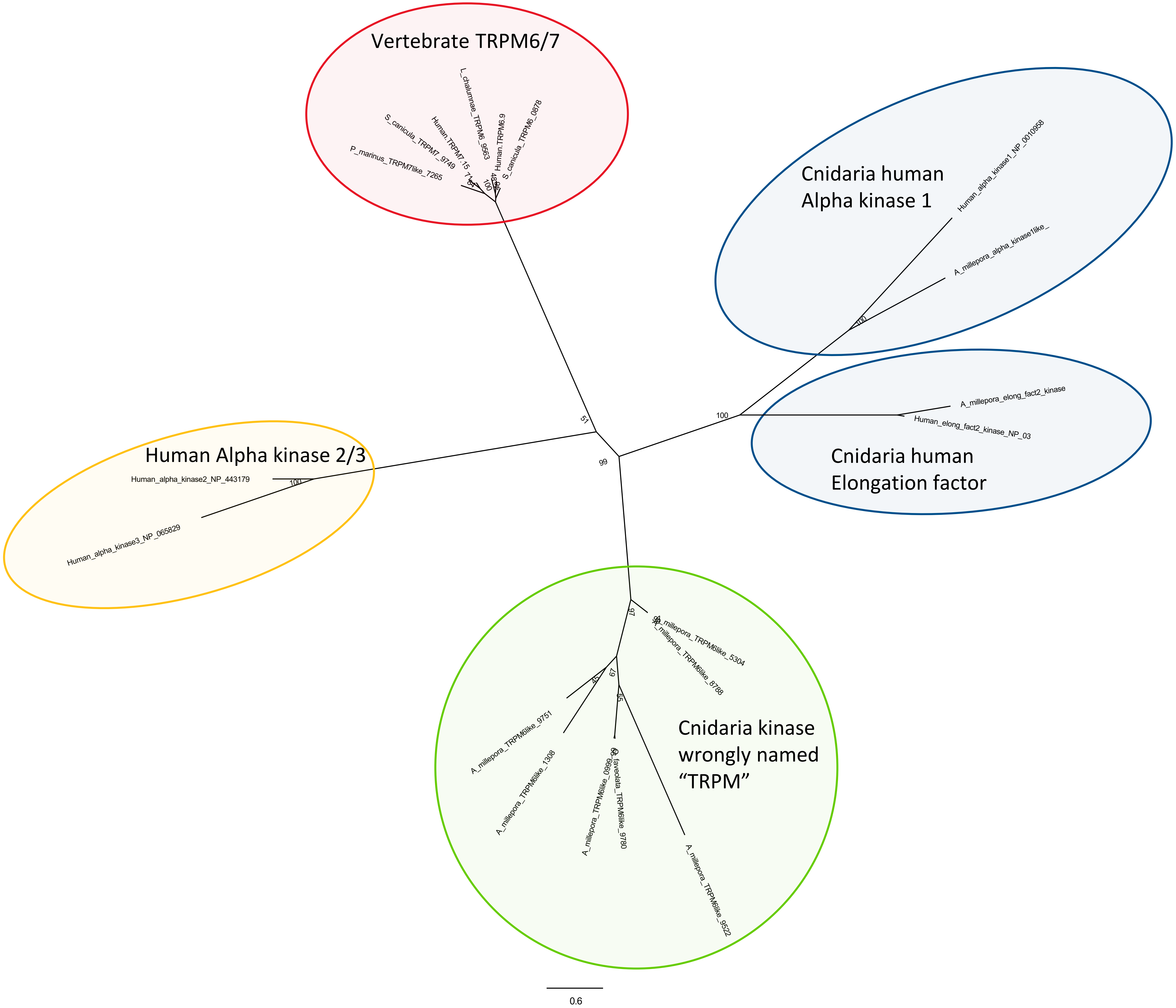

Supplementary Figure S8A. Alignment of vertebrate mir-211 and mir-204 sequences

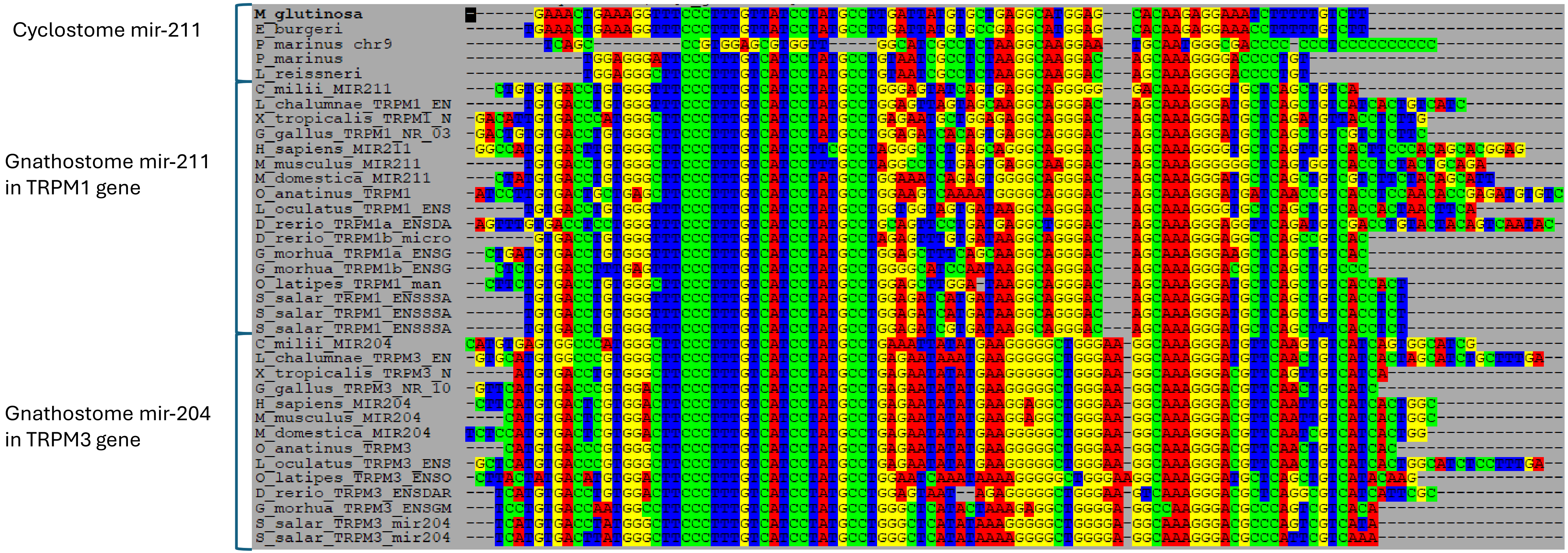

Supplementary Figure S8B. Phylogenetic tree of vertebrate mir-211 and mir-204 sequences

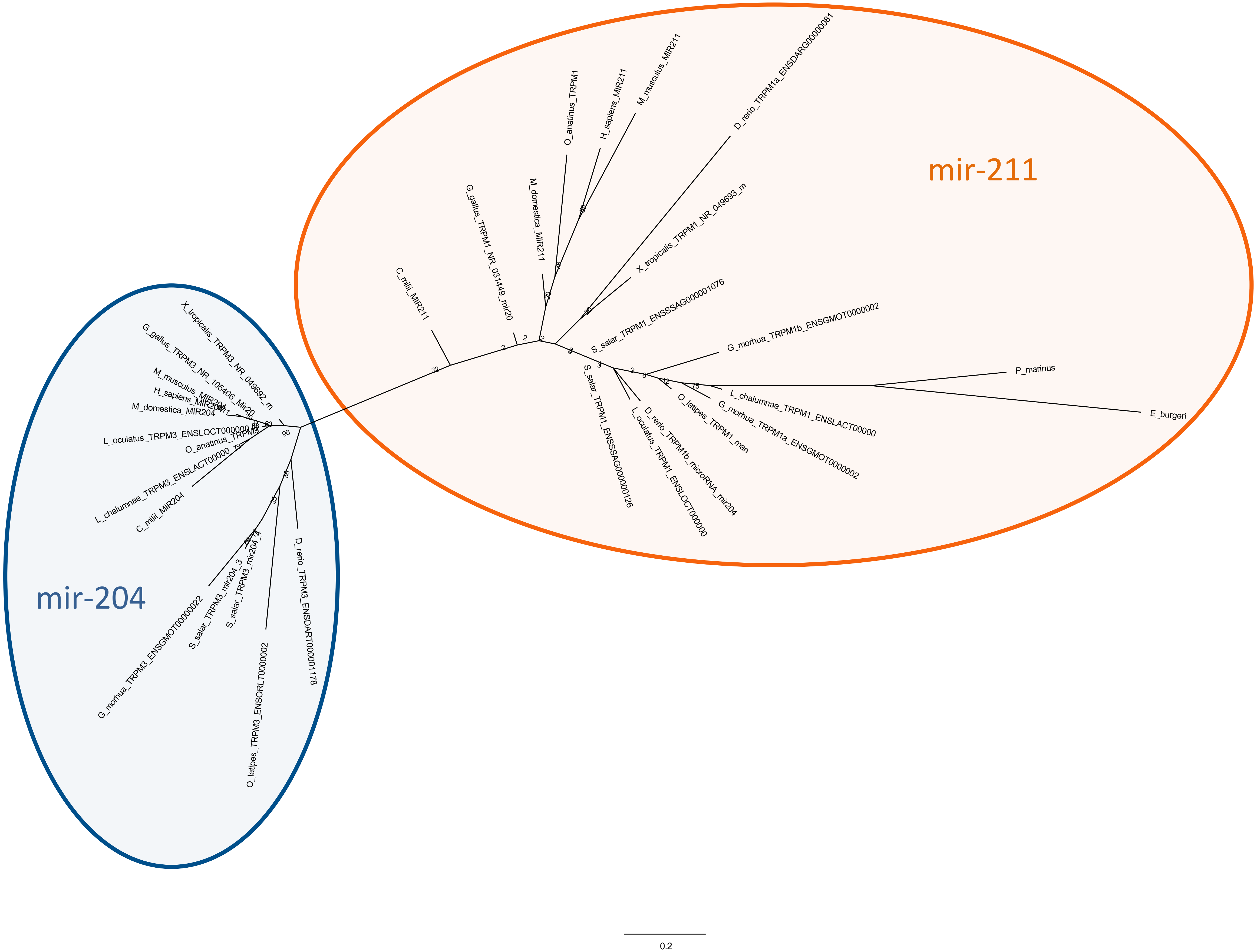

Supplement: msag098_Supplementary_Data [file msag098_supplementary_data.zip › Morini_Revised_Supplementary_Figures copy.pdf]
